# The SARS-CoV-2 main protease M^pro^ causes microvascular brain pathology by cleaving NEMO in brain endothelial cells

**DOI:** 10.1038/s41593-021-00926-1

**Published:** 2021-10-21

**Authors:** Jan Wenzel, Josephine Lampe, Helge Müller-Fielitz, Raphael Schuster, Marietta Zille, Kristin Müller, Markus Krohn, Jakob Körbelin, Linlin Zhang, Ümit Özorhan, Vanessa Neve, Julian U. G. Wagner, Denisa Bojkova, Mariana Shumliakivska, Yun Jiang, Anke Fähnrich, Fabian Ott, Valentin Sencio, Cyril Robil, Susanne Pfefferle, Florent Sauve, Caio Fernando Ferreira Coêlho, Jonas Franz, Frauke Spiecker, Beate Lembrich, Sonja Binder, Nina Feller, Peter König, Hauke Busch, Ludovic Collin, Roberto Villaseñor, Olaf Jöhren, Hermann C. Altmeppen, Manolis Pasparakis, Stefanie Dimmeler, Jindrich Cinatl, Klaus Püschel, Matija Zelic, Dimitry Ofengeim, Christine Stadelmann, François Trottein, Ruben Nogueiras, Rolf Hilgenfeld, Markus Glatzel, Vincent Prevot, Markus Schwaninger

**Affiliations:** 1 Institute for Experimental and Clinical Pharmacology and Toxicology, Center of Brain, Behavior and Metabolism (CBBM), University of Lübeck, Lübeck, Germany; 2 DZHK (German Research Centre for Cardiovascular Research), Hamburg-Lübeck-Kiel and Frankfurt, Germany; 3 Department of Oncology, Hematology & Bone Marrow Transplantation, University Medical Center Hamburg-Eppendorf, Hamburg, Germany; 4 Institute of Molecular Medicine, University of Lübeck, Lübeck, Germany; 5 German Center for Infection Research (DZIF), partner site Hamburg–Lübeck–Borstel–Riems, Lübeck, Germany; 6 Institute for Cardiovascular Regeneration, Cardiopulmonary Institute (CPI), University Frankfurt, Frankfurt, Germany; 7 Institute of Medical Virology, University Frankfurt, Frankfurt, Germany; 8 Institute of Experimental Dermatology, University of Lübeck, Lübeck, Germany; 9 Institute for Cardiogenetics, University of Lübeck, Lübeck, Germany; 10 Centre d’Infection et d’Immunité de Lille, Inserm U1019, CNRS UMR 9017, University of Lille, CHU Lille, Institut Pasteur de Lille, Lille, France; 11 Institute of Medical Microbiology, Virology and Hygiene, University Medical Center Hamburg-Eppendorf, Hamburg, Germany; 12 Univ. Lille, Inserm, CHU Lille, Laboratory of Development and Plasticity of the Neuroendocrine Brain, Lille Neuroscience & Cognition, UMR-S 1172, DISTALZ, EGID, Lille, France; 13 Institute of Neuropathology, University Medical Center, Göttingen, Germany; 14 Campus Institute for Dynamics of Biological Networks, University of Göttingen, Göttingen, Germany; 15 Max Planck Institute for Experimental Medicine, Göttingen, Germany; 16 Airway Research Center North, Member of the German Center for Lung Research (DZL), Lübeck, Germany; 17 Institute of Anatomy, University of Lübeck, Lübeck, Germany; 18 Roche Pharma Research and Early Development (pRED), Roche Innovation Center, Basel, Switzerland; 19 Institute of Neuropathology, University Medical Center Hamburg-Eppendorf, Hamburg, Germany; 20 Institute for Genetics, University of Cologne, Cologne, Germany; 21 Institute of Legal Medicine, University Medical Center Hamburg-Eppendorf, Hamburg, Germany; 22 Rare and Neurologic Diseases Research, Sanofi, Framingham, MA USA; 23 Department of Physiology, CIMUS, University of Santiago de Compostela-Instituto de Investigación Sanitaria, Santiago de Compostela, Spain

**Keywords:** Blood-brain barrier, Central nervous system infections

## Abstract

Coronavirus disease 2019 (COVID-19) can damage cerebral small vessels and cause neurological symptoms. Here we describe structural changes in cerebral small vessels of patients with COVID-19 and elucidate potential mechanisms underlying the vascular pathology. In brains of severe acute respiratory syndrome coronavirus 2 (SARS-CoV-2)-infected individuals and animal models, we found an increased number of empty basement membrane tubes, so-called string vessels representing remnants of lost capillaries. We obtained evidence that brain endothelial cells are infected and that the main protease of SARS-CoV-2 (M^pro^) cleaves NEMO, the essential modulator of nuclear factor-κB. By ablating NEMO, M^pro^ induces the death of human brain endothelial cells and the occurrence of string vessels in mice. Deletion of receptor-interacting protein kinase (RIPK) 3, a mediator of regulated cell death, blocks the vessel rarefaction and disruption of the blood–brain barrier due to NEMO ablation. Importantly, a pharmacological inhibitor of RIPK signaling prevented the M^pro^-induced microvascular pathology. Our data suggest RIPK as a potential therapeutic target to treat the neuropathology of COVID-19.

## Abstract (web summary)

A novel study led by scientists in Lübeck, Germany, shows that SARS-CoV-2-infected brain endothelial cells undergo cell death due to the cleavage of NEMO by the viral protease M^pro^, potentially causing cerebral COVID-19 and ‘long COVID’ symptoms.

## Main

In December 2019, the newly discovered SARS-CoV-2 virus emerged and was identified as the causative agent of COVID-19. Within a few months, COVID-19 developed into a pandemic with millions of people infected worldwide and a high death load. Symptoms typically originate from the respiratory tract; however, in many patients, other organ systems are involved, causing symptoms that are not secondary to respiratory failure or the severe systemic inflammation due to pneumonia. A considerable proportion of patients, up to 84% of those with severe COVID-19, show neurological signs and symptoms including anosmia, epileptic seizures, strokes, loss of consciousness and confusion^1–3^. Typically, COVID-19 can present with the clinical picture of encephalopathy^2^. Beyond 4 weeks after onset, the post-acute COVID-19 syndrome includes cognitive impairment and a range of psychiatric symptoms and may affect up to 76% of patients^4^. Although a direct infection of the brain remains a matter of debate, SARS-CoV-2 viral genomes were detected in the brain and cerebrospinal fluid (CSF) of some patients, supporting the notion that SARS-CoV-2 gains access to the brain^3,5,6^. Viral RNA has been found in blood and virus-like particles or viral proteins in brain endothelial cells^5,7–10^, suggesting that SARS-CoV-2 reaches the brain by a hematogenous route. In line with a vascular infection, endothelial cells in other organs have been identified as targets of SARS-CoV-2 infection^11,12^. In patients with COVID-19, magnetic resonance imaging detected lesions that are compatible with a cerebral small-vessel disease and with a disruption of the blood–brain barrier (BBB)^13–15^. Autopsy studies have confirmed this interpretation^15–19^. However, the microvascular pathology and the underlying mechanisms in COVID-19 are still unclear.

In brains of SARS-CoV-2-infected patients, as well as mouse and hamster models, we found an increase in empty vascular basement membrane tubes, so-called string vessels, reflecting microvascular pathology. The SARS-CoV-2 genome encodes two viral proteases that are responsible for processing the viral polyproteins into the individual components of the replication and transcription complexes. We found that one of them, SARS-CoV-2 M^pro^ (also called Nsp5 or 3CL^pro^)^20^, cleaves the host protein nuclear factor (NF)-κB essential modulator (NEMO). NEMO is involved in signaling cascades that regulate the transcription of numerous genes, including the antiviral type I interferons and other immune genes^21^. Beyond gene regulation, NEMO modulates cell survival and prevents apoptosis and necroptosis^22^. The ablation of NEMO in brain endothelial cells induced microvascular pathology in mice that was reminiscent of what we observed in brains of patients with COVID-19. Of note, the widespread death of endothelial cells, rarefaction of capillaries, disruption of the BBB and neuroinflammation due to NEMO ablation were prevented by deleting receptor-interacting protein kinase 3 (*Ripk3*), a protein kinase that is essential for regulated cell death. Importantly, a pharmacological inhibitor of RIPK signaling prevented the microvascular pathology induced by M^pro^. These data suggest a potential therapeutic option to interfere with the neurological consequences of COVID-19.

## Results

### Microvascular brain pathology in SARS-CoV-2 infection

**Fig. 1:**
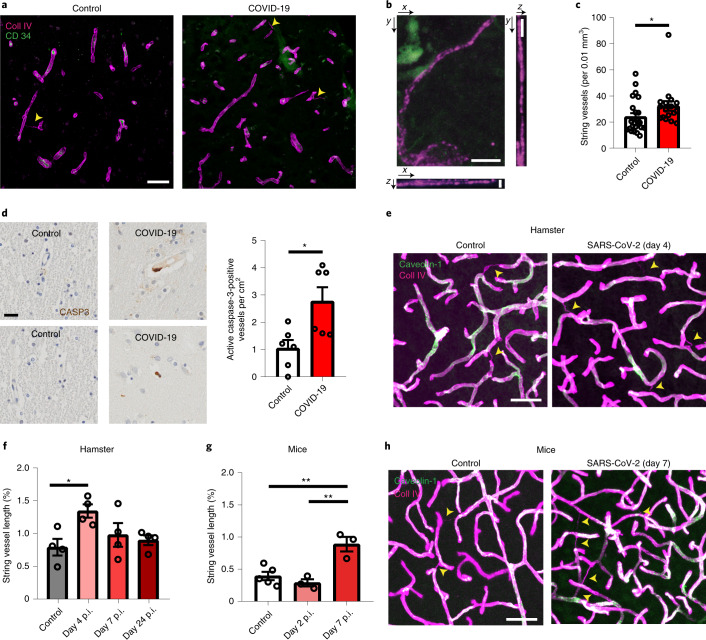
SARS-CoV-2 infection is associated with increased string vessels in the brain. **a**–**c**, In the brains of SARS-CoV-2-infected patients, empty basement membrane tubes, also known as string vessels (arrowheads), were increased in the frontal cortex. Sections were stained for the basement membrane marker collagen IV (coll IV) and the endothelial marker CD34. Representative images in **a** and **b** were obtained from the dataset in **c**. **a**, Scale bar, 50 μm. **b**, Magnified maximal projection of a *z*-stack of a string vessel with orthogonal views to exclude that these are partial sections of capillaries. Scale bars, 3 μm. **c**, Quantification of string vessels per image volume. *N* = 23 control patients, *N* = 17 COVID-19 patients. **d**, Immunostaining revealed a higher number of active caspase-3-positive vessels in cortical sections of SARS-CoV-2-infected patients (*N* = 6) than in controls (*N* = 6). Representative images and quantification are shown. Scale bar, 20 µm. **e**,**f**, SARS-CoV-2-infected hamsters developed an increased number of string vessels as shown by co-staining for coll IV and the endothelial marker caveolin-1. **e**, Representative images of coll IV and caveolin-1 in the cortex of hamsters 4 d post infection (p.i.) with SARS-CoV-2 and of uninfected hamsters. Scale bar, 50 µm. **f**, Quantification of string vessel lengths as a percentage of total vessel length in SARS-CoV-2-infected hamsters at 4, 7 and 24 d p.i. and in uninfected controls (*N* = 4 hamsters per group). **g**,**h**, SARS-CoV-2-infected K18-hACE2 mice developed an increase in string vessels as shown by co-staining for coll IV and caveolin-1. **g**, Quantification of string vessel lengths as a percentage of total vessel length in SARS-CoV-2-infected K18-hACE2 mice 2 d p.i. (*N* = 3 mice) and 7 d p.i. (*N* = 3 mice) and in uninfected controls (*N* = 5 mice). **h**, Representative images of coll IV and caveolin-1 in the cortex of K18-hACE2 mice 7 d p.i. and of uninfected K18-hACE2 animals. Scale bar, 50 µm. *P < 0.05, **P < 0.01. Means ± s.e.m. are shown. *N* denotes the number of patients or animals. Detailed information on the exact test statistics, sidedness and values is provided in Supplementary Table 5.

**Extended Data Fig. 1:**
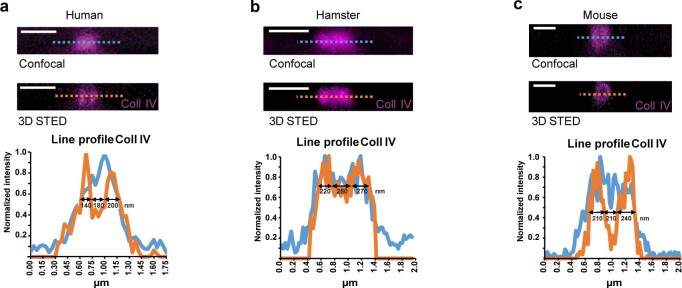
String vessels have a tube-like structure. **a, b, c**, Super-resolution imaging of human (**a**), hamster (**b**) and mouse string vessels (**c**). In confocal mode with the pinhole closed to 0.4 AU, the string vessels appeared as solid collagen tubes. However, STED microscopy demonstrated a tube-like structure of string vessels in humans, hamsters and mice. In all species, the apparent diameter of string vessels was about 500 – 1,000 nm with the collagen walls having a similar thickness. Images are representative for at least 3 experiments per species. Scale bar, 1 µm.

**Extended Data Fig. 2:**
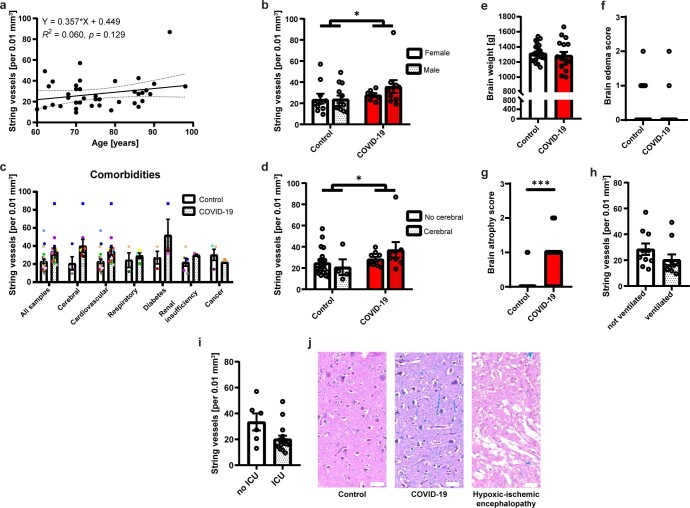
Characteristics of the COVID-19 and control patients. **a**, Age did not affect the number of string vessels per volume of the image. N = 40 patients. **b**, String vessels were increased in SARS-CoV-2-infected patients independent of sex. N = 10 female and 13 male control, N = 7 female and 10 male COVID-19 patients. **c**, Comorbidities were equally distributed across control and COVID-19 patients. For characteristics of the patients, refer to Fig. 1c and Supplementary Tables 1-2. N = 23 control, N = 17 COVID-19 patients. **d**, String vessels were increased in SARS-CoV-2-infected patients independent of cerebral comorbidities. N = 19 control patients without and 4 with cerebral comorbidity, N = 9 COVID-19 patients without and 8 with cerebral comorbidity. **e**, Brain weight was not different between COVID-19 and control patients. **f**, Brain edema score was not different between COVID-19 and control patients. N = 23 control, N = 17 COVID-19 patients. Medians are shown. **g**, Brain atrophy score was increased in COVID-19 compared to control patients. N = 23 control, N = 17 COVID-19 patients. Medians are shown. **h**, String vessels of control patients with or without ventilation are shown. N = 10 patients per group. **i**, String vessels of control patients who were treated in an intensive care unit (ICU) or not are shown. N = 6 no ICU, N = 16 ICU. **j**, None of the COVID-19 or control patients showed evidence for a hypoxic-ischemic encephalopathy, also known as ‘respirator brain’. Characteristic morphological features of eosinophilic neurons, laminar changes in the cerebral cortex or loss of neurons were not detectible. Representative images of a combined HE and Nissl staining of brain sections from a control and COVID-19 patient as well as a brain section from a patient with hypoxic-ischemic encephalopathy are depicted. Scale bar, 50 µm. Means ± s.e.m. are shown if not stated otherwise. * *p* < 0.05 *** *p* < 0.001. Detailed information about the exact test statistics, sidedness and values are provided in Supplementary Table 5.

In patients with COVID-19, magnetic resonance imaging and neuropathological studies have reported parenchymal lesions that are compatible with a small-vessel disease^13,15–18^. To search for the underlying microvascular pathology, we stained sections of the frontal cortex for the endothelial cell marker CD34 and the basement membrane component collagen IV. We noted an increase in thin collagen IV-positive strings lacking CD34 staining (Fig. 1a,b). These so-called string vessels were interpreted as remnants of capillaries after endothelial cells have died^23^. Super-resolution microscopy showed that string vessels are tube-like structures with a typical diameter of 0.5–1 µm (Extended Data Fig. 1). To test the association of string vessels with COVID-19, we investigated 17 SARS-CoV-2-infected patients and 23 control patients with similar age and sex distribution (Supplementary Table 1 and 2 and Extended Data Fig. 2). Counting revealed a significant increase in string vessels in patients with SARS-CoV-2 (Fig. 1c). The difference persisted when we stratified the two groups according to comorbidities or sex (Extended Data Fig. 2b–d). Because of pneumonia, more SARS-CoV-2-infected patients were ventilated than controls (Supplementary Table 2). However, neither ventilation nor intensive care unit (ICU) treatment as indicators of severe respiratory disease affected string vessels in control patients (Extended Data Fig. 2h,i), arguing against the possibility that string vessels could be caused by systemic hypoxia. In support, we did not find morphological signs of a global hypoxic–ischemic encephalopathy in any of the patients (Extended Data Fig. 2j). In line with the notion that string vessels are formed when endothelial cells die, we observed cells that stained for the apoptosis marker active caspase-3 in brain microvessels. These cells were rare in brain sections of the frontal cortex but significantly more frequent in SARS-CoV-2-infected patients than in controls (Fig. 1d).

To exclude unknown confounding factors that may have occurred in the retrospective clinical study, we sought to reproduce the findings in two animal models of SARS-CoV-2 infection. In hamsters infected with SARS-CoV-2, the length of string vessels increased on day 4 after infection (Fig. 1e,f and Supplementary Fig. 1a). Interestingly, the increase was transient and normalized at later time points. A similar finding was observed in K18-hACE2 mice, which express the human angiotensin-converting enzyme 2 (hACE2) receptor, under the control of the keratin 18 promoter. In this model, SARS-CoV-2 infection induced the formation of string vessels in a time-dependent manner (Fig. 1g,h and Supplementary Fig. 1b). In summary, the data show that SARS-CoV-2 infection induces a microvascular pathology in the brain in the form of string vessels.

### Evidence that SARS-CoV-2 can infect brain endothelial cells

**Fig. 2:**
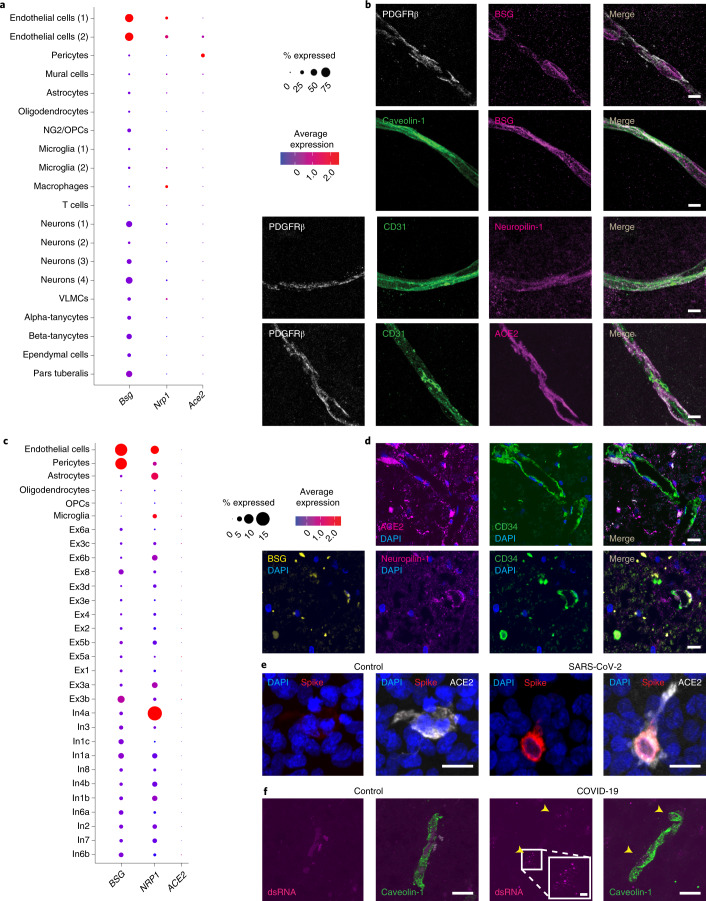
Brain endothelial cells express SARS-CoV-2 receptors in mice and humans. **a**, RNA-seq in single mouse brain cells characterized the cell-type-specific expression of the SARS-CoV-2 receptors *Ace2*, *Bsg* and *Nrp1*. Colors represent mean gene expression, and diameters denote the percentage of positive cells in the 20 cell clusters. Uniform manifold approximation and projection (UMAP) plot and dot plot for marker genes are shown in Extended Data Fig. 3. **b**, Representative images of mouse brain co-stained for ACE2, BSG, NRP1, the endothelial markers CD31 or caveolin-1 and the pericyte marker PDGFRβ. BSG and NRP1 were co-localized with caveolin-1 or CD31, respectively, but not with PDGFRβ. The staining was reproduced in at least six mice for each marker. Noteworthy, in the scRNA-seq analysis, the number of *Ace2* mRNA-positive cells was low and did not fully reflect the number of ACE2-positive cells identified by immunostainings. In immunostainings, almost all pericytes and tanycytes^59^ were positive for ACE2, in contrast to the scRNA-seq data. Scale bars, 5 µm. VLMCs, vascular and leptomeningeal cells; OPCs, oligodendrocyte progenitor cells. **c**, Cell-type-specific expression of *ACE2*, *BSG* and *NRP1* in a previously published single nuclear RNA-seq profile of human brain^28^. Gene expression of *ACE2*, *BSG* and *NRP1* is shown as dot plots for all 30 clusters. UMAP plot and dot plot for marker genes is shown in Extended Data Fig. 4. **d**, Representative images of the human frontal cortex co-stained for ACE2, BSG and NRP1 with the endothelial marker CD34 confirmed the cell-type-specific localization of the receptors in the vascular unit. ACE2, BSG and NRP 1 were co-localized with the endothelial protein CD34. Images were obtained from a dataset of three patients (three sections per patient). Scale bars, 5 µm. **e**, Human brain endothelial hCMEC/D3 cells were transfected with human *ACE2* and were incubated with SARS-CoV-2 (multiplicity of infection (MOI) of 1). Twenty-four hours after exposure to the virus, the spike glycoprotein was detected in several ACE2-positive cells indicating infection. The experiment was performed three times. Scale bars, 5 µm. **f**, dsRNA was found in caveolin-1-positive endothelial cells in the cortex of two of four patients with COVID-19 but not in three uninfected controls. Scale bar, 20 µm and 2 µm (inset).

**Extended Data Fig. 3:**
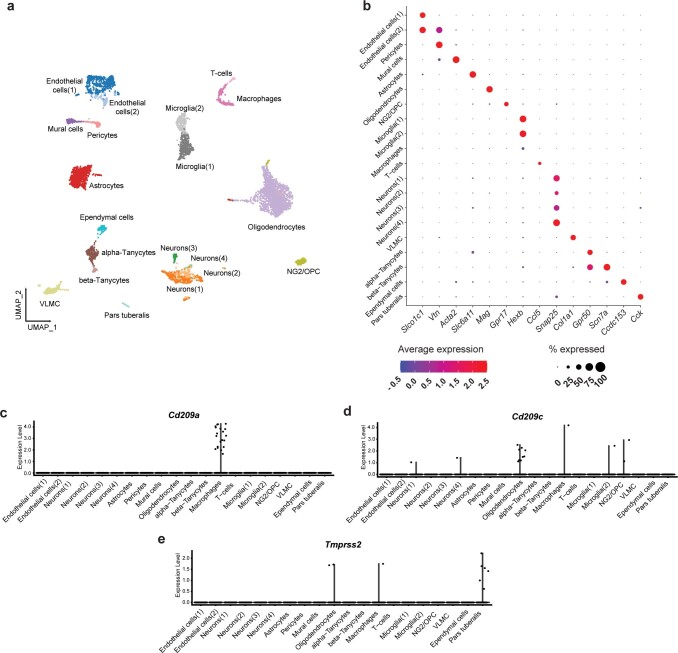
Clustering of mouse brain cells according to single-cell RNA sequencing. **a**, UMAP plot of 5,611 mouse brain cells. Clusters were annotated according to marker genes for the specific cell types. **b**, Dot plots of marker genes used for annotation. Gene expression for all 20 clusters is shown as dot plot with the color representing mean gene expression and the diameters showing the percentage of positive cells in the clusters. **c-e**, mRNA expression patterns of the putative SARS-CoV-2 entry factors *Cd209a* (**c**), *Cd209c* (**d**), and *Tmprss2* (**e**). There were no *Cd209b-* or *Cd209d*-expressing cells. VLMC, vascular and leptomeningeal cells; OPC, oligodendrocyte progenitor cells.

To approach the question of whether SARS-CoV-2 can infect brain endothelial cells, we determined the expression of membrane receptors and enzymes that are known to facilitate the entry of SARS-CoV-2 in host cells, namely ACE2, neuropilin-1 (*Nrp1*), and possibly basigin (*Bsg*), Cd209a, Cd209b, Cd209c, Cd209d and Tmprss2 (refs. ^3,8,24–27^). When analyzing isolated mouse brain cells by single-cell RNA sequencing (scRNA-seq), we identified 20 cell clusters, including two endothelial clusters (Extended Data Fig. 3a,b). Some cells in the endothelial cell cluster 2 expressed *Ace2*, albeit at a lower level than pericytes (Fig. 2a). Imaging confirmed that ACE2 was expressed in mouse cerebral microvessels, but co-staining of endothelial and pericytic markers and high-resolution microscopy demonstrated that ACE2 was mainly localized in pericytes (Fig. 2b). In contrast, we observed high levels of *Nrp1* and *Bsg* expression in mouse endothelial cells by scRNA-seq and immunostaining (Fig. 2a,b) but no *Cd209a*, *Cd209b*, *Cd209c*, *Cd209d* or *Tmprss2* (Extended Data Fig. 3c–e).

**Extended Data Fig. 4:**
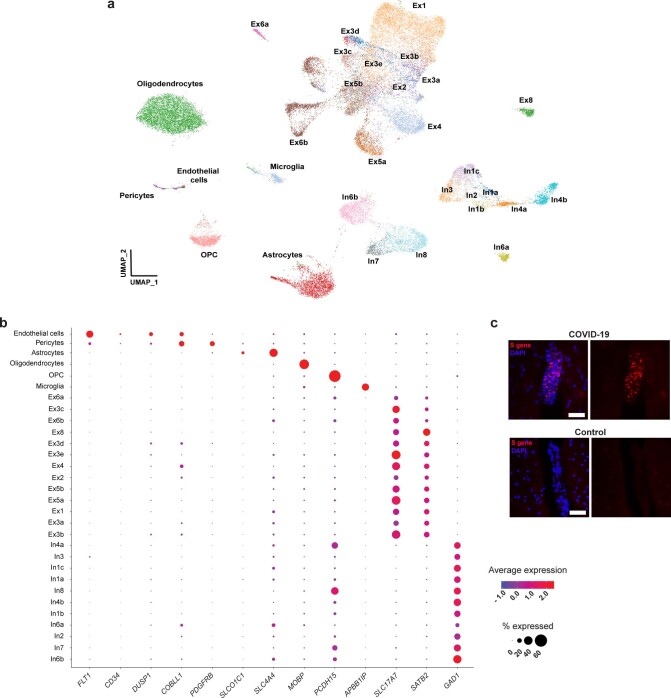
Clustering of human brain cells according to single nuclear RNA sequencing. **a**, UMAP plot of 35,289 human brain cells. Clusters were annotated according to marker genes for the specific cell types. The dataset has been published (Lake, B.B., et al. Integrative single-cell analysis of transcriptional and epigenetic states in the human adult brain. *Nat Biotechnol* 36, 70-80 (2018)). **b**, Dot plots of marker genes used for annotation. Gene expression for all 30 clusters is shown as dot plot with the color representing mean gene expression and the diameters showing the percentage of positive cells in the clusters. Ex, excitatory neuronal subtypes; In, inhibitory neuronal subtypes. **c**, The SARS-CoV-2 S gene encoding the spike protein was found in vascular cells in the cortex of a COVID-19 patient (exemplary for at least 3 vessels) but not in an uninfected control subject by *in situ* hybridization. Scale bar, 50 µm.

To evaluate the expression of *NRP1*, *BSG* and *ACE2* in human brain endothelial cells, we analyzed a published single-nuclei RNA-seq dataset that allowed the identification of endothelial and pericyte cell clusters^28^ (Extended Data Fig. 4a,b). In line with the mouse data, *BSG* and *NRP1* were highly expressed in endothelial cells (Fig. 2c). In this study, *ACE2* was below the detection limit in all neural cell clusters, although other studies have reported *ACE2* expression in the human brain^25,29^. Immunofluorescence staining confirmed the presence of BSG and NRP1 in brain endothelial cells, while only a few cells were positive for ACE2 (Fig. 2d). Because brain endothelial cells express NRP1, BSG and low levels of ACE2 as potential receptors for SARS-CoV-2, we asked whether the cells are susceptible to a SARS-CoV-2 infection. Because endothelial expression of *ACE2* decreases in vitro due to the lack of blood flow^30,31^, we transfected cultured brain endothelial hCMEC/D3 cells with an expression plasmid of *ACE2* and added SARS-CoV-2. In ACE2-positive cells, but not ACE2-negative cells, the spike protein was found in a perinuclear localization as reported for pulmonary endothelial cells (Fig. 2e)^32^. In accordance with the concept that SARS-CoV-2 can infect brain endothelial cells^5,8–10^, we detected double-stranded RNA (dsRNA) in endothelial cells (Fig. 2f) and the S gene of SARS-CoV-2 encoding the spike protein in cerebral vessels (Extended Data Fig. 4c) of a patient with COVID-19. Together, these data show that brain endothelial cells express receptors for SARS-CoV-2 and are susceptible to an infection.

### M^pro^ cleaves NEMO

**Extended Data Fig. 5:**
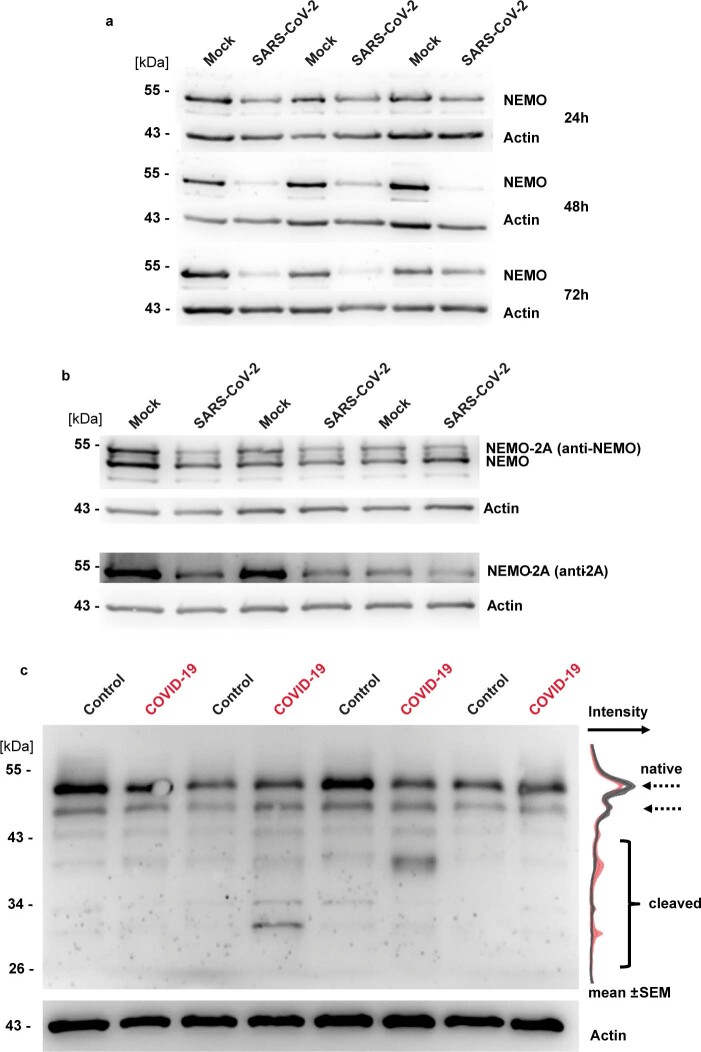
SARS-CoV-2 induces NEMO cleavage. **a**, SARS-CoV-2- (MOI: 1) or mock-infected Vero E6 cells were harvested at the indicated time points. Cell lysates were immunoblotted for NEMO. N = 3 experiments. **b**, Human brain endothelial hCMEC/D3 cells were transfected with plasmids encoding for NEMO-2A and for human ACE2 plus TMPRSS2. Twenty-four hours after transfection, cells were incubated with SARS-CoV-2 for 2 hours (MOI: 1) and harvested after additional 24 hours. Only transfected cells expressing human ACE2 as well as 2A-tagged NEMO were susceptible to SARS-CoV-2 infection (Fig. 2e). Accordingly, 2A-tagged NEMO but not untagged NEMO was degraded. Upper panel, immunoblot using anti-NEMO; lower panel, immunoblot using anti-2A antibodies. N = 3 experiments. **c**, Lysates of the medulla oblongata of SARS-CoV-2-infected and control patients were immunoblotted for NEMO. Samples were matched according to post-mortem intervals (PMI): lanes 1 and 2: 1 day PMI; lanes 3 and 4: 2–3 days PMI; lanes 5 and 6: 4–5 days PMI; lanes 7 and 8: 4–5 days PMI. Histogram intensities of each lane were measured and depicted as means ± s.e.m., showing a decrease of the native forms of NEMO and an increase in cleaved NEMO in the tissue of SARS-CoV-2-infected patients (red profile) in comparison to control subjects (black profile). N = 4 patients in each group. Source data

SARS-CoV-2 manipulates host cells to promote its survival and propagation. We hypothesized that it might be a successful evolutionary strategy for SARS-CoV-2 to cleave NEMO as an essential component of the antiviral immune response^21^. Indeed, NEMO was degraded in infected Vero E6 and hCMEC/D3 cells before the cells died (Extended Data Fig. 5a,b). Moreover, immunoblots of NEMO in brain lysates of SARS-CoV-2-infected and control patients indicated that NEMO was cleaved in some of the infected patients (Extended Data Fig. 5c).

**Fig. 3:**
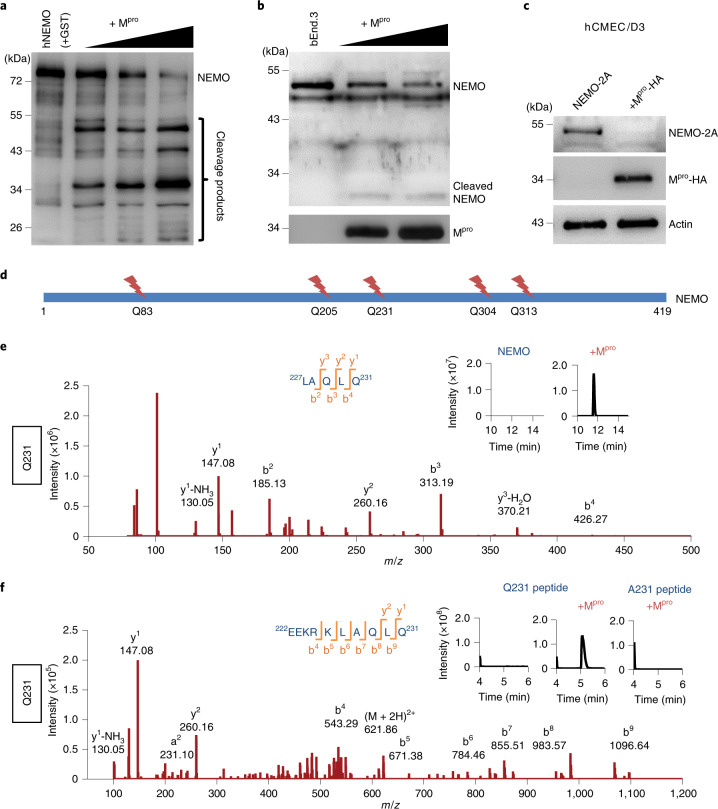
M^pro^ cleaves NEMO. **a**, SARS-CoV-2 M^pro^ in increasing concentrations (0, 5, 10 and 25 µM; 120 min) degraded full-length human NEMO (fused to GST) while several cleavage products emerged (representative of at least six experiments at different conditions). The full immunoblots are shown in Extended Data Fig. 6. **b**, Mouse NEMO in bEnd.3 cell extracts was cleaved to a short fragment after incubation with increasing concentrations of M^pro^ (0, 5 and 10 µM) for 120 min (representative of three experiments). **c**, In human brain endothelial hCMEC/D3 cells, M^pro^-HA degraded NEMO-2A. After transfecting the cells with pCAG-NEMO-2A-GFP ± pCAG-M^pro^-HA, immunoblots of cell lysates were performed (representative of at least nine experiments). **d**, Tryptic digestion and tandem mass spectrometry (MS/MS) analysis identified five M^pro^ cleavage sites in human NEMO as illustrated in the schematic. For the protein sequence, see Supplementary Fig. 2k. **e**, M^pro^ cleaved human NEMO at Q231. An extracted ion chromatogram of the tryptic peptide ^227^LAQLQ^231^ (*m/z*, 572.3414^2+^; retention time (RT), 11.6 min) derived from NEMO after incubation with M^pro^ (5 µM, inset) and the MS/MS spectrum that was used for peptide identification are shown. The experiment was performed once. **f**, A synthetic peptide corresponding to the human NEMO sequence confirmed that Q231 is an M^pro^ cleavage site. Total ion chromatograms after incubation of the synthetic peptide h-NEMO_222-241 (EEKRKLAQLQVAYHQLFQEY) in the presence or absence of M^pro^ (2.5 µM, inset) are shown. In the presence of M^pro^, the proteolysis product ^222^EEKRKLAQLQ^231^ (*m/z*, 414.9109^3+^; RT, 5.1 min) was detected. The mutant peptide h-NEMO-Q231A_222-241 (EEKRKLAQLAVAYHQLFQEY) was not cleaved by M^pro^ (inset). The MS/MS spectrum of the peptide ^222^EEKRKLAQLQ^231^ is shown (representative of five experiments with the synthetic peptide h-NEMO_222-241 and four experiments with the mutant peptide h-NEMO-Q231A_222-241). Source data

**Extended Data Fig. 6:**
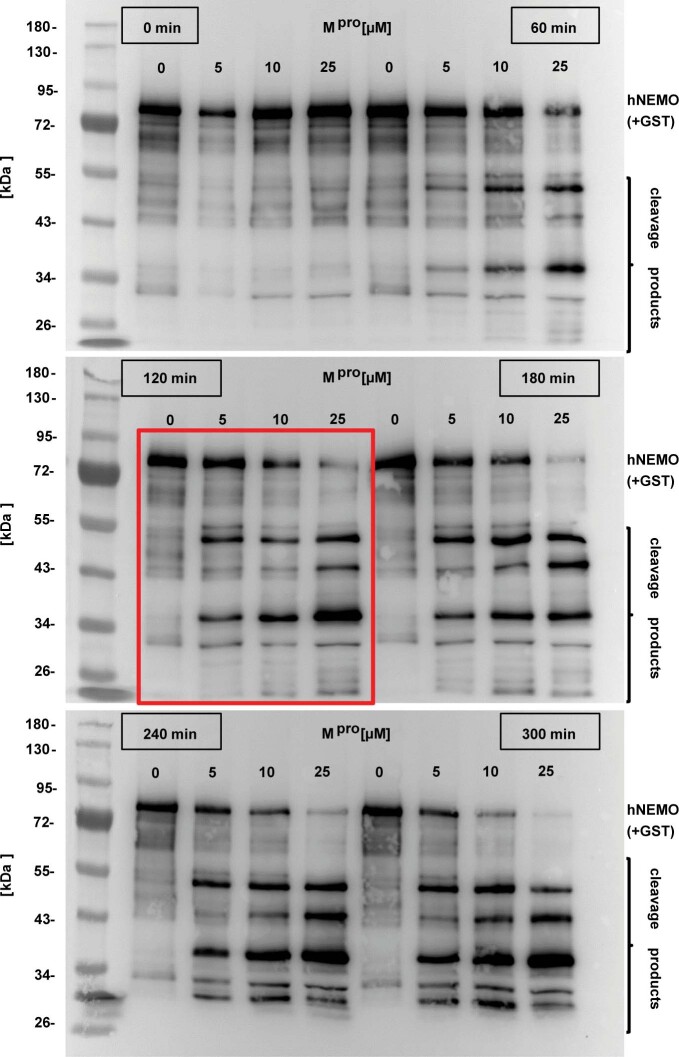
Time dependence of NEMO cleavage by M^pro^. Cleavage of human NEMO by SARS-CoV-2 M^pro^ (0, 5, 10, 25 µM) depended on the incubation time. NEMO (with GST tag) or the cleavage products were detected by immunoblotting (representative for at least 6 experiments at different conditions). The red-boxed part of the blot is shown in Fig. 3a. Source data

**Extended Data Fig. 7:**
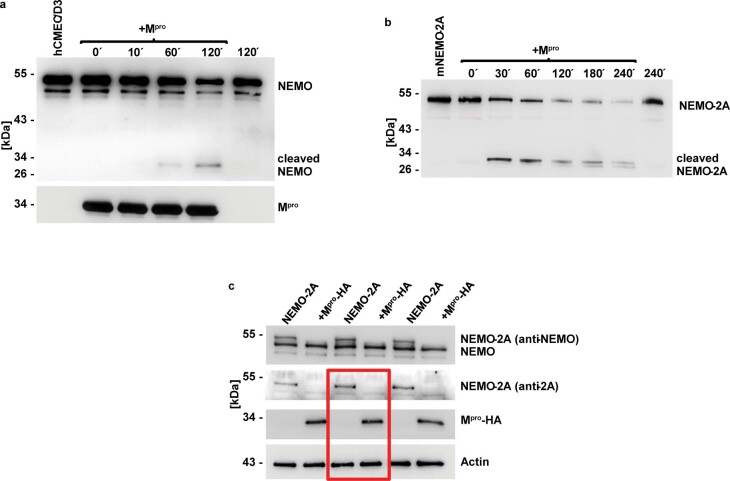
Cleavage of NEMO in cell extracts. **a**, Cell lysates of the human brain endothelial cell line hCMEC/D3 were incubated with M^pro^ (25 µM) for different durations and blotted against NEMO and M^pro^ (representative for at least 3 experiments at different conditions). **b**, HEK293T cells were transfected with a plasmid encoding for the mouse NEMO linked to GFP via a 2A sequence. After cell lysis, M^pro^ (25 µM) was added and incubated for the indicated durations. Antibodies against the 2A linker were used to identify NEMO and its cleavage products (representative for 2 experiments). **c**, hCMEC/D3 cells were transfected with the same plasmid as described in b (pNEMO-2A-GFP) and additionally with an empty control plasmid or a plasmid containing the HA-tagged form of the SARS-CoV-2 M^pro^ protease. After 2 days, cells were lysed and blotted against NEMO, 2A, M^pro^, and actin (representative for at least 9 experiments). The red-boxed part of the blots is shown in Fig. 3c. Source data

The SARS-CoV-2 genome encodes two proteases, M^pro^ and the papain-like protease^20^. Purified M^pro^ cleaved recombinant human NEMO as well as human and mouse NEMO in extracts of brain endothelial or HEK293T cells in a dose-dependent and time-dependent manner (Fig. 3a,b and Extended Data Figs. 6 and 7a,b). Importantly, M^pro^ cleaved NEMO also in intact brain endothelial cells (Fig. 3c and Extended Data Fig. 7c). When coexpressed with NEMO-2A-GFP that self-processes to NEMO-2A and green fluorescent protein (GFP), HA-tagged M^pro^ completely neutralized NEMO-2A in human hCMEC/D3 cells.

In vitro, M^pro^ produced several NEMO fragments (Fig. 3a and Extended Data Fig. 6). Tryptic digestion of M^pro^-treated NEMO followed by mass spectrometry of the generated peptides showed that cleavage occurred at Q83, Q205, Q231, Q304 and Q313 (Fig. 3d,e and Supplementary Fig. 2a–j). The cleavage sites that we identified (Supplementary Fig. 2k) resemble other known recognition sequences of M^pro^ (ref. ^20^). We verified the cleavage at Q231 by using synthetic peptides as substrates corresponding to both the human and mouse NEMO sequence (Fig. 3f and Supplementary Fig. 3a–c). With the human NEMO sequence at Q231, the apparent catalytic efficiency (about 43 M^−1^ s^−1^) was in the range that has been reported for the cleavage site between Nsp4 and Nsp5 (Supplementary Fig. 3d)^33^. In keeping with the central role of Q in the recognition sequence, the mutation p.Gln231Ala in the human NEMO sequence prevented cleavage by M^pro^ at this site (Fig. 3f).

**Fig. 4:**
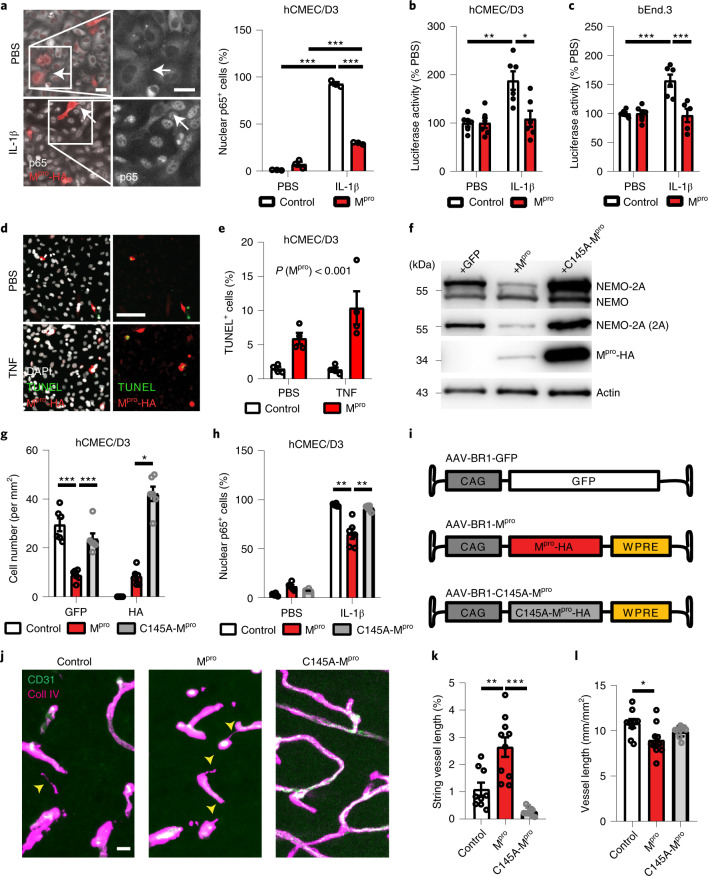
M^pro^ inactivates NEMO and induces brain endothelial cell loss mimicking COVID-19-associated brain pathology. **a**, M^pro^-HA inhibited the nuclear translocation of the NF-κB subunit p65 in hCMEC/D3 cells stimulated with IL-1β (0.25 µg ml^−1^) for 30 min. Cells (*N* = 3 wells per group; representative of three independent experiments) were transfected with a control plasmid (Bluescript) or pCAG-M^pro^-HA. Scale bar, 25 µm. **b**,**c**, M^pro^-HA blocked the activation of NF-κB by IL-1β (0.25 µg ml^−1^) in human (**b**) and mouse (**c**) brain endothelial cells. Cells were transfected with pNF-κB-Luc plus a control plasmid or pCAG-M^pro^-HA. *N* = 5–6 wells per group. **d**,**e**, M^pro^-HA induced death of hCMEC/D3 cells, especially after exposure to TNF (100 ng ml^−1^; 4.5 h). Cells (*N* = 12 wells per group) were transfected with a control plasmid or pCAG-M^pro^-HA. Scale bar, 100 µm. **f**, In hCMEC/D3 cells, M^pro^ degraded NEMO-2A, whereas the inactive variant p.Cys145Ala-M^pro^ did not. After transfecting the cells with pCAG-NEMO-2A-GFP plus pCAG-GFP as control, pCAG-M^pro^-HA or pCAG-p.Cys145Ala-M^pro^-HA, immunoblots of cell lysates were performed (representative of at least six experiments). **g**, More hCMEC/D3 cells survived after expressing the inactive variant p.Cys145Ala-M^pro^-HA than after expressing M^pro^-HA. All cells were transfected with pCAG-GFP in parallel. The numbers of GFP^+^ or HA^+^ cells are depicted (*N* = 6 wells per group). **h**, In contrast to M^pro^, p.Cys145Ala-M^pro^ did not inhibit the nuclear translocation of the NF-κB subunit p65 when hCMEC/D3 cells were stimulated with IL-1β (0.25 µg ml^−1^) for 30 min. Cells were transfected with control plasmid, pCAG-M^pro^-HA or pCAG-p.Cys145Ala-M^pro^-HA (*N* = 6 wells per group). **i**, Schematic of AAV-BR1 vectors to transduce brain endothelial cells in vivo. WPRE, woodchuck hepatitis posttranscriptional regulatory element. **j**, AAV-BR1-M^pro^ but not AAV-BR1-p.Cys145Ala-M^pro^ led to the formation of string vessels (arrowheads) in the brain of mice. Representative images were taken in the cortex. Scale bar, 20 µm. **k**, Quantification of string vessel length as a percentage of total vessel length (*N* = 9–10 mice per group). **l**, Total vessel length was reduced after mice received AAV-BR1-M^pro^ but not AAV-BR1-p.Cys145Ala-M^pro^ (*N* = 9–10 mice per group). *P < 0.05, **P < 0.01, ***P < 0.001. Means ± s.e.m. are shown. Detailed information on the exact test statistics, sidedness and values is provided in Supplementary Table 5. Source data

NEMO is an essential component of the canonical pathway leading to the activation of NF-κB by inflammatory factors such as interleukin (IL)-1β. Supporting the functional relevance of NEMO cleavage, M^pro^ blocked NF-κB activation. When expressed in human brain endothelial hCMEC/D3 cells, M^pro^ prevented the nuclear translocation of the NF-κB subunit p65, reflecting its activation in response to IL-1β (Fig. 4a). M^pro^ also abolished the stimulation of NF-κB-mediated gene transcription by IL-1β, which we investigated in hCMEC/D3 cells and mouse brain endothelial bEnd.3 cells using luciferase reporter gene assays (Fig. 4b, c). Thus, we obtained unequivocal evidence that M^pro^ cleaves and thereby inactivates NEMO.

### M^pro^-induced damage mimics microvascular pathology

As NEMO is required for the integrity of some but not all cell types^22^, the question arose whether the M^pro^-mediated cleavage of NEMO compromises endothelial survival. To test whether M^pro^ induces endothelial cell death, we transfected hCMEC/D3 cells with a plasmid encoding M^pro^-HA and treated the cells with tumor necrosis factor (TNF) to model the elevated TNF serum concentrations in patients with COVID-19 (ref. ^34^). M^pro^-expressing cells were more often positive for the cell death marker TUNEL, especially when exposed to TNF (Fig. 4d,e), demonstrating that M^pro^ promotes endothelial cell death. To test whether protease activity and NEMO cleavage are required for the toxic effects of M^pro^, we mutated the amino acid Cys145 in the catalytic site to alanine (p.Cys145Ala-M^pro^)^20^. This mutation abrogates the protease activity^35^. As expected, p.Cys145Ala-M^pro^ did not cleave NEMO-2A when coexpressed with NEMO-2A-GFP (Fig. 4f). In these experiments, levels of the HA-tagged p.Cys145Ala-M^pro^ were higher than those of M^pro^, probably reflecting a better cell viability with the mutated protease. In support of this concept, the number of GFP-positive hCMEC/D3 cells dropped when M^pro^-HA but not p.Cys145Ala-M^pro^-HA were co-transfected (Fig. 4g). In contrast to M^pro^, p.Cys145Ala-M^pro^ did not inhibit NF-κB activation by IL-1β (Fig. 4h). These data show that NEMO cleavage and cell toxicity by M^pro^ depend on its protease activity.

To explore the function of M^pro^ in vivo, we used the adeno-associated viral vector AAV-BR1, which selectively targets brain endothelial cells when injected intravenously^36^ (Fig. 4i). After administering AAV-BR1 vectors encoding GFP or HA-tagged proteins, the accumulation of genomic particles in the brain was similar between the vector groups, and about 10–20% of cerebral capillaries expressed GFP or the HA epitope, while different anti-M^pro^ antibodies did not detect the tagged protein (Fig. 4i and Supplementary Fig. 4). When mice received AAV-BR1-M^pro^ 2 weeks before, we observed an increased number of string vessels and a decreased vascular density in their brains (Fig. 4j–l). The inactive p.Cys145Ala-M^pro^ had no effect on string vessels or vascular density. In line with a detrimental effect of M^pro^ on endothelial cell survival, there were less HA-positive vessels after expression of M^pro^-HA in comparison to p.Cys145Ala-M^pro^-HA (Supplementary Fig. 4c). M^pro^-induced string vessels were indistinguishable from those that had occurred in SARS-CoV-2-infected patients.

### Capillaries are at risk from ablating NEMO

**Fig. 5:**
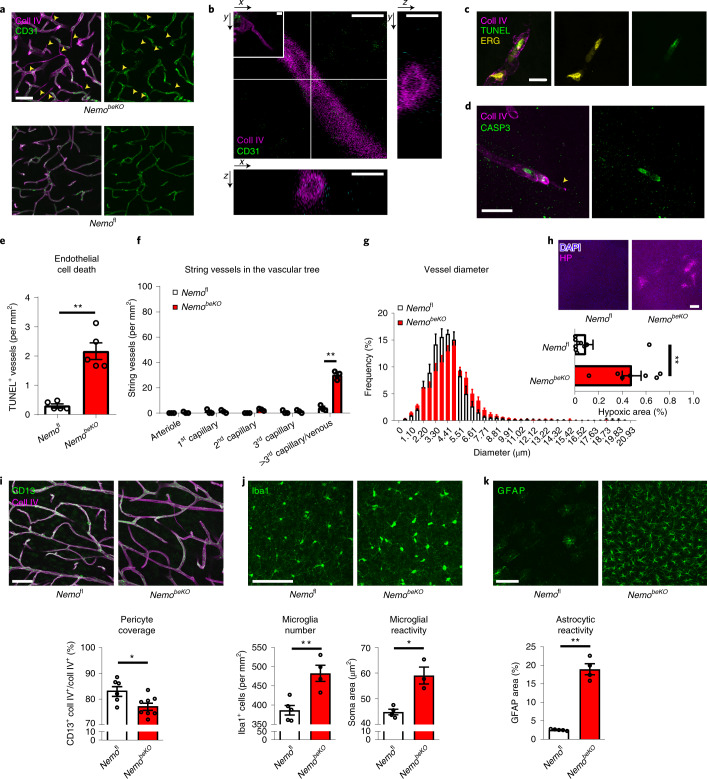
Brain endothelial loss of NEMO induces string vessel formation and influences the neurovascular unit. **a**, Cerebral microvasculature of *Nemo*^*beKO*^ mice deficient of NEMO in brain endothelial cells and *Nemo*^*fl*^ controls. Arrowheads indicate string vessels. Confocal images are representative of five *Nemo*^*fl*^ and five *Nemo*^*beKO*^ mice (six sections per animal) 28 d after tamoxifen treatment. Scale bar, 50 µm. **b**, STED microscopy showed that string vessels are thin tubes with a typical diameter of 0.5–1 µm. The image is representative of three *Nemo*^*beKO*^ mice. Scale bars, 1 µm. **c**,**d**, String vessels (arrowhead) were often adjacent to dying, TUNEL^+^ or active caspase-3^+^ endothelial cells in *Nemo*^*beKO*^ mice. ERG co-staining indicated TUNEL^+^ nuclei in the endothelium. Representative images in **c** were obtained from the dataset in **e**. Images in **d** are representative of two *Nemo*^*beKO*^ mice. Scale bar, 20 µm. **e**, Increased numbers of TUNEL^+^ cells localized in collagen IV-stained microvessels of *Nemo*^*beKO*^ mice (*N* = 5 mice per genotype). **f**, More string vessels were present in higher branch orders of the vascular tree in the cortices of *Nemo*^*beKO*^ mice (*N* = 3 mice per genotype). Branch orders were defined by α-SMA staining of arterioles (Extended Data Fig. 8a). **g**, *Nemo*^*beKO*^ mice (*N* = 3 mice per genotype) preferentially lost vessels with small diameters. The diameters of collagen IV^+^ vessels are shown as a histogram. **h**, *Nemo*^*beKO*^ mice (*N* = 7) demonstrated patchy cerebral hypoxia detected by the hypoxia probe (HP) in contrast to *Nemo*^*fl*^ controls (*N* = 10). **i**, Pericyte coverage of vessels was reduced in *Nemo*^*beKO*^ mice (*N* = 8) compared to *Nemo*^*fl*^ controls (*N* = 6). Scale bar, 50 µm. **j**, *Nemo*^*beKO*^ mice (*N* = 3–4) showed an increased number of activated microglia cells as shown by increased Iba1^+^ soma area in comparison to that of *Nemo*^*fl*^ controls (*N* = 4–5). Scale bar, 100 µm. **k**, *Nemo*^*beKO*^ mice (*N* = 4) demonstrated astrogliosis as shown by an increased GFAP^+^ area in the cortex compared to that of *Nemo*^*fl*^ controls (*N* = 5). Scale bar, 100 µm. *P < 0.05, **P < 0.01. Means ± s.e.m. are shown. Detailed information on the exact test statistics, sidedness and values is provided in Supplementary Table 5.

**Fig. 6:**
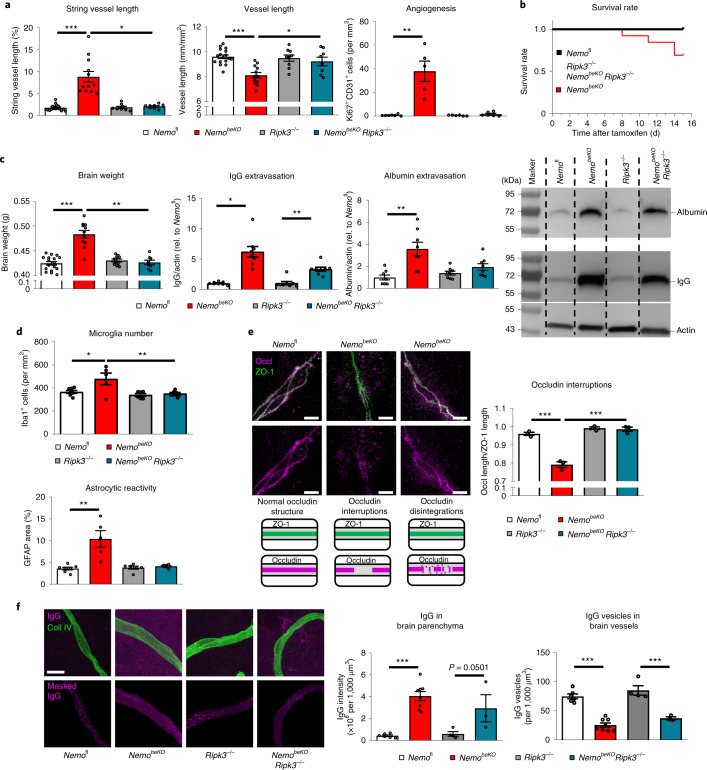
RIPK3 mediates microvascular pathology induced by NEMO ablation. **a**, *Ripk3* deletion prevented vascular pathology of *Nemo*^*beKO*^ mice, that is, string vessel formation and rarefaction of vessels (*N* = 17 *Nemo*^*fl*^, 12 *Nemo*^*beKO*^, 9 *Ripk3*^−/−^ and 8 *Nemo*^*beKO*^*Ripk3*^−/−^ mice), as well as endothelial proliferation (Ki67^+^ endothelial cells; *N* = 7 *Nemo*^*fl*^, 5 *Nemo*^*beKO*^, 6 *Ripk3*^−/−^ and 6 *Nemo*^*beKO*^*Ripk3*^−/−^ mice). **b**, *Ripk3* deletion normalized the survival of *Nemo*^*beKO*^ animals (*N* = 13) that was significantly reduced in comparison to control mice (*N* = 17; log-rank Mantel–Cox test, *P* = 0.015). In contrast, all *Nemo*^*beKO*^*Ripk3*^−/−^ mice (*N* = 8) survived. **c**, *Ripk3* deletion largely attenuated the disruption of the BBB in *Nemo*^*beKO*^ mice. Brain weight reflects brain edema (*N* = 17 *Nemo*^*fl*^, 11 *Nemo*^*beKO*^, 10 *Ripk3*^−/−^ and 8 *Nemo*^*beKO*^*Ripk3*^−/−^ mice). IgG and albumin extravasation were determined by immunoblots of brain tissue (*N* = 8 mice per genotype). **d**, RIPK3 ablation abolished glial activation. *Nemo*^*beKO*^ but not *Nemo*^*beKO*^*Ripk3*^−/−^ mice showed a higher number of Iba1^+^ microglia/macrophages and an increased area of GFAP^+^ astrocytes (*N* = 7 *Nemo*^*fl*^, 5 *Nemo*^*beKO*^, 6 *Ripk3*^−/−^ and 6 *Nemo*^*beKO*^*Ripk3*^−/−^ mice). **e**, Immunostainings of ZO-1 and occludin obtained by expansion microscopy in *Nemo*^*fl*^ and *Nemo*^*beKO*^ mice, representative of a dataset of three animals for each genotype. Scale bar, 10 µm (corresponding to approximately a 2.5-µm initial size). We classified tight junction structures as occludin interruptions or disintegrations shown as blurring of the occludin structure (middle). **f**, *Nemo*^*beKO*^*Ripk3*^−/−^ mice showed less IgG extravasation than Nemo^beKO^ mice and a similar decrease in the number of IgG vesicles in brain endothelial cells as *Nemo*^*beKO*^ mice. Scale bar, 10 µm. IgG extravasation was measured in IgG immunostainings as fluorescence intensity outside the vasculature normalized for parenchymal volume, and IgG vesicles were quantified inside vessels and normalized for the vessel volume (*N* = 6 *Nemo*^*fl*^, 8 *Nemo*^*beKO*^, 4 *Ripk3*^−/−^ and 3 *Nemo*^*beKO*^*Ripk3*^−/−^ mice). *P < 0.05, **P < 0.01, ***P < 0.001. Means ± s.e.m. are shown. Detailed information on the exact test statistics, sidedness and values is provided in Supplementary Table 5. Source data

**Extended Data Fig. 8:**
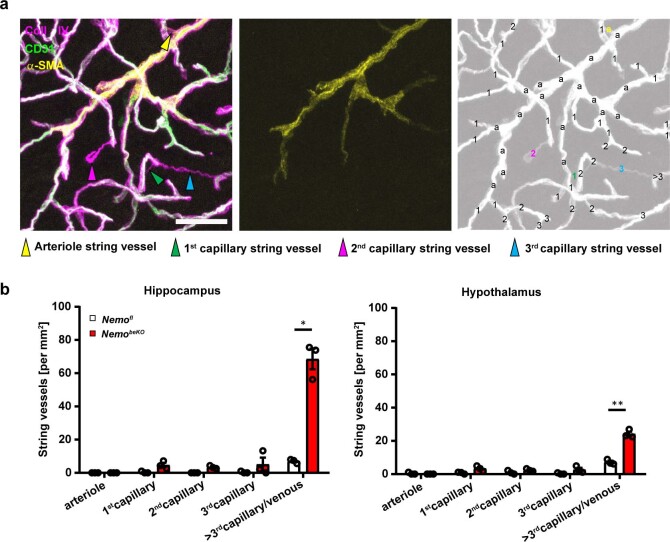
String vessels occur in the deeper part of the vascular tree. **a**, String vessels were tracked in confocal 3D scans of the cortex after staining against CD31, collagen IV and smooth muscle actin (α-SMA) (left). Since α-SMA is expressed in arteries and arterioles (middle), the branching order can be determined (right). String vessels positive for α-SMA are defined as arteriole string vessels, adjacent string vessels as 1^st^ capillary string vessels, etc. (right). The image was obtained by cropping a section from a *Nemo*^*beKO*^ tilescan from the dataset used for quantification in (b) (N = 3 animals). Scale bar, 50 µm. **b**, Tracking of string vessels in the hippocampus and hypothalamus of *Nemo*^*beKO*^ mice revealed that more string vessels were present in higher branch orders of the vascular tree in *Nemo*^*beKO*^ mice. N = 3 animals. Means ± s.e.m. are shown, *P < 0.05, **P < 0.01. Detailed information about the exact test statistics, sidedness and values are provided in Supplementary Table 5.

To test whether NEMO ablation could be responsible for the vascular pathology observed with endothelial M^pro^ expression, we used a mouse model of inducible *Ikbkg* (*Nemo*) deletion in brain endothelial cells (*Nemo*^*beKO*^)^37^. Similar to the M^pro^-mediated cleavage of NEMO, its genetic ablation led to numerous string vessels in the brain (Fig. 5a,b). STED microscopy showed that string vessels are tube-like structures with a similar morphology in humans, hamsters and *Nemo*^*beKO*^ or K18-hACE2 mice (Fig. 5b and Extended Data Fig. 1a–c). They were deficient of endothelial cells. Our interpretation of string vessels as a sign of endothelial demise is based on the following observations. NEMO deletion induced endothelial cell death as detected by the TUNEL reaction or by staining for active caspase-3 (Fig. 5c–e). Most of them were observed in higher-order capillaries (Fig. 5f and Extended Data Fig. 8), suggesting that capillaries are particularly susceptible to NEMO deficiency. Consequently, small-diameter vessels were predominantly lost and mice developed patchy hypoxia in the brain (Fig. 5g,h) but not in peripheral organs (Supplementary Fig. 5a,b). Finally, *Nemo* deletion led to a significant vessel rarefaction in the brain (Fig. 6a) and to a BBB disruption (Fig. 6c,f and Supplementary Fig. 5c).

The loss of endothelial cells induced by NEMO deficiency also affected other cell types in the neurovascular unit. While the overall coverage of vessels by pericytes was slightly lowered (Fig. 5i), the number of microglia increased and they exhibited an activated morphology (Fig. 5j). The astrogliosis marker glial fibrillary acidic protein (GFAP) was strongly upregulated, indicating an inflammatory response (Fig. 5k).

### NEMO ablation induces vascular pathology via RIPK signaling

Microglia orchestrate the neuroinflammatory response in the brain, including the activation of astrocytes^38^. Therefore, we tested their role in the microvascular pathology induced by NEMO deficiency. However, ablating microglia by administering the CSF-1R antagonist PLX5622 did neither prevent the formation of string vessels nor prevent the activation of astrocytes (Supplementary Fig. 6).

**Extended Data Fig. 9:**
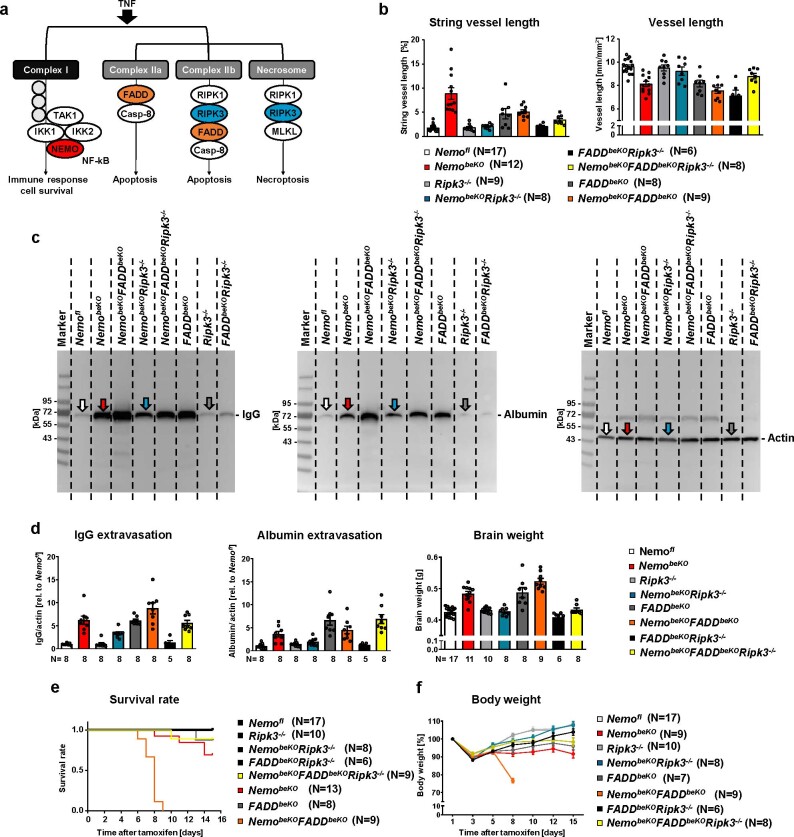
Modulation of apoptosis and necroptosis pathways in mice with NEMO deficient brain endothelial cells. **a**, Scheme showing the role of RIPK3 and FADD in cell death caused by NEMO deficiency. TNF leads to the activation of several adaptor proteins and kinases, such as TAK1. Activation of the IκB kinase complex triggers the translocation of NF-κB into the nucleus and the expression of target genes. NEMO is the regulatory subunit of the IκB kinase complex and is essential for NF-κB pathway activation. In the absence of NEMO, TNF leads to the formation of complex IIa, complex IIb, and the necrosome and induces apoptosis or necroptosis. According to current concepts (Kondylis, V., Kumari, S., Vlantis, K. & Pasparakis, M. The interplay of IKK, NF-kappaB and RIPK1 signaling in the regulation of cell death, tissue homeostasis and inflammation. *Immunol Rev* 277, 113-127 (2017)), apoptosis is mediated by FADD and RIPK3, while necroptosis depends on RIPK3. **b**, Deletion of *Fadd* in brain endothelial cells (*FADD*^*beKO*^) led to the formation of string vessels and reduced vessel lengths. The results obtained in *Nemo*^*fl*^, *Nemo*^*beKO*^, *Ripk3*^−*/*−^, and *Nemo*^*beKO*^*Ripk3*^−*/*−^ mice are also included in Fig. 6 of the main article. **c**, Representative immunoblots showing the extravasation of IgG and albumin into the brain tissue. In total, 9 blots were analyzed with one replicate per animal. **d**, Quantification of the immunoblots normalized for actin levels demonstrated a disruption of the BBB in FADD-deficient mice as shown by the extravasation of IgG and albumin. Brain weights hint at higher water content in FADD deficient mice. **e**, *Nemo*^*beKO*^*FADD*^*beKO*^ mice died within 9 days after tamoxifen injection while RIPK3 deficiency prevented the death of *Nemo*^*beKO*^ mice. **f**, *Ripk3* deletion normalized the body weight of mice with brain endothelial NEMO deficiency, while FADD deficiency worsened body weight loss. Only mice that survived until the end of the experiment were analyzed. Means ± s.e.m. are shown. Detailed information of group sizes are indicated in the individual figure. Source data

To develop alternative therapeutic options, we considered previous reports that NEMO blocks apoptosis or necroptosis in epithelial cells^22^. In *Nemo*^*beKO*^ mice, we inactivated the Fas-associated death domain protein (FADD), a component of apoptosis signaling and RIPK3, a kinase central for both necroptosis and apoptosis (Extended Data Fig. 9a). FADD deficiency did not ameliorate the consequences of *Nemo* deletion but enhanced the damage (Extended Data Fig. 9b–f). FADD not only mediates apoptosis but also inhibits necroptosis, explaining the detrimental effect of FADD deletion^39,40^.

In contrast, RIPK3 deficiency, which by itself did not affect the cerebral microvasculature, prevented the formation of string vessels and the rarefaction of cerebral vessels due to *Nemo* deletion (Fig. 6a). Probably as a response to the vessel rarefaction, NEMO deficiency stimulated endothelial proliferation indicating angiogenesis, which has been described in patients with COVID-19 previously^11^. Notably, *Ripk3* deletion abrogated endothelial proliferation (Fig. 6a). *Ripk3* deletion also normalized survival, brain weight and body weight of mice with a NEMO deficiency in brain endothelial cells and reduced the extravasation of IgG and albumin into the parenchyma, showing that disruption of the BBB was mitigated (Fig. 6b,c and Extended Data Fig. 9c–f). Consequentially, *Nemo* deletion did not activate microglia or astrocytes in the absence of RIPK3 (Fig. 6d).

To explore the mechanisms of BBB protection by RIPK3 deficiency, we quantified the levels of the tight junction protein occludin. NEMO deficiency led to interruptions and the disintegration of occludin-positive tight junctions, which was prevented by RIPK3 deficiency (Fig. 6e). In addition to endothelial tight junctions, the BBB is characterized by a low rate of transcytosis in cerebral capillaries. The increased IgG extravasation in *Nemo*^*beKO*^ mice was associated with a lower number of IgG-filled vesicles in brain endothelial cells (Fig. 6f). Super-resolution imaging confirmed the decrease of IgG-loaded vesicles, although the density of all vesicles that were detected by transmission electron microscopy was not reduced (Supplementary Fig. 7b,c). We have observed a similar reduction of IgG-filled vesicles despite increased overall IgG extravasation in *Pdgfb*^*Ret/Ret*^ mice, indicating that the detected population of IgG-filled vesicles limit IgG permeation across the BBB^41^. RIPK3 deficiency did not counteract the effect of NEMO ablation on IgG transcytosis (Fig. 6f). Therefore, we conclude that RIPK3 deficiency improves the BBB tightness of *Nemo*^*beKO*^ mice mainly by preventing endothelial cell death and rescuing tight junctions.

**Fig. 7:**
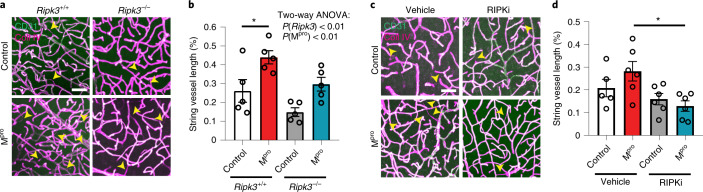
Inactivation of RIPK protects against M^pro^-induced vascular pathology. **a**,**b**, In *Ripk3*^−/−^ mice, the M^pro^-mediated increase in string vessels (arrowheads) was reduced. **a**, Representative images of microvessels in the cortex of *Ripk3*^+/+^ and *Ripk3*^−/−^ mice, 2 weeks after intravenous application of the control vector AAV-BR1-GFP (3.3 × 10^11^ genome particles per mouse) or AAV-BR1-M^pro^ (3.3 × 10^11^ genome particles per mouse). Brain sections were stained for coll IV and CD31. Scale bar, 50 µm. **b**, Quantification of string vessel length as a percentage of total vessel length. The M^pro^-induced string vessel formation in *Ripk3*^+/+^ mice was reduced in *Ripk3*^−/−^ mice (*N* = 5 mice per genotype). **c**,**d**, In RIPK1 inhibitor (RIPKi)-treated mice, the M^pro^-induced increase in string vessels was reduced. **c**, Representative images of microvessels in the cortex of mice, 2 weeks after intravenous application of the control vector AAV-BR1-GFP (3.3 × 10^11^ genome particles per mouse) or AAV-BR1-M^pro^ (3.3 × 10^11^ genome particles per mouse) and oral treatment with RIPKi or vehicle. Scale bar, 50 µm. **d**, Quantification of string vessel length as a percentage of total vessel length. RIPKi prevented the M^pro^-induced string vessel formation (*N* = 5 control-vehicle, 6 M^pro^-vehicle, 6 control-RIPKi and 6 M^pro^-RIPKi mice). *P < 0.05; means ± s.e.m. are shown. Detailed information on the exact test statistics, sidedness and values is provided in Supplementary Table 5. ANOVA, analysis of variance.

**Extended Data Fig. 10:**
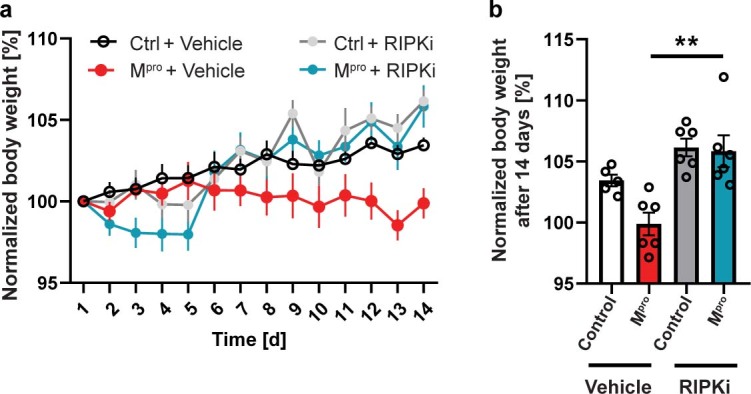
RIPK inhibition normalizes body weight gain in AAV-BR1-M^pro^-injected mice. **a**, RIPKi treatment normalized the body weight gain in AAV-BR1-M^pro^-injected mice (N = 5 Ctrl + Vehicle mice, 6 M^pro^ + Vehicle mice, 6 Ctrl + RIPKi mice, 6 M^pro^ + RIPKi mice). **b**, In AAV-BR1-M^pro^-injected mice, the cumulative body weight gain over 14 days was normalized by RIPKi treatment (N = 5 Ctrl + Vehicle mice, 6 M^pro^ + Vehicle mice, 6 Ctrl + RIPKi mice, 6 M^pro^ + RIPKi mice). Means ± s.e.m. are shown, ** *P* < 0.01. Detailed information about the exact test statistics, sidedness and values are provided in Supplementary Table 5.

Importantly, the M^pro^-induced string vessel formation also depended on the presence of RIPK3 (Fig. 7a,b), suggesting that inhibitors of RIPK signaling may protect against the microvascular pathology induced by M^pro^. Although RIPK3 inhibitors block necroptosis, they may induce apoptotic cell death, limiting their translational potential^42^. Therefore, we turned to a small-molecule inhibitor of the upstream kinase RIPK1 that activates RIPK3. The RIPK1 inhibitor abrogated M^pro^-induced string vessel formation (Fig. 7c,d) and normalized body weight gain that was impaired by M^pro^ expression in brain endothelial cells (Extended Data Fig. 10). Overall, these data indicate that inhibitors of RIPK signaling may prevent cerebral microvascular pathology in COVID-19.

## Discussion

In many patients with COVID-19, neurological and psychiatric symptoms occur during the acute disease and determine the post-acute syndrome^4^. Here, we show microvascular pathology in the brains of SARS-CoV-2-infected patients that likely explains signs and symptoms, although systemic effects, including respiratory failure and cytokine release, may contribute to CNS symptoms. Furthermore, we propose a mechanism by which SARS-CoV-2 infection compromises brain endothelial function, damages the BBB and reduces CNS perfusion.

In the brains of SARS-CoV-2-infected patients, we found an increase in string vessels. This finding was confirmed in two animal models of SARS-CoV-2 infection. String vessels are empty basement membrane tubes that often contain pericyte processes. In our view, string vessels are similar or, at least in part, identical to tunneling nanotubes that have been implicated in regulating cerebrovascular coupling^43^. Irrespective of such a function, the association of string vessels with endothelial cell death, BBB disruption and brain ischemia is strong^23,44^, especially because blocking cell death prevented string vessel formation and other changes (Figs. 6 and 7). Therefore, from a technical perspective, string vessels provide a straightforward quantitative measure of capillary damage that allowed a careful statistical comparison between SARS-CoV-2-infected patients and controls. We propose that death of brain endothelial cells in COVID-19 is secondary to their SARS-CoV-2 infection. Although several groups have provided converging evidence for endothelial infection^5,8,11,45^, others have questioned this, mainly based on doubts as to whether endothelial cells express ACE2 (refs. ^29,31^). The jury is still open whether ACE2-positive endothelial cells represent a special endothelial subpopulation or a contamination with pericytes. While ACE2 seems to be essential for SARS-CoV-2 infection, soluble ACE2 may substitute for its lack of expression in certain cell types^46^, suggesting a way how SARS-CoV-2 may infect brain endothelial cells even if they do not express ACE2. The presence of NRP1 and possibly BSG in brain endothelial cells facilitates SARS-CoV-2 cell entry and infectivity^8,24,27,47^.

How could SARS-CoV-2 infection induce the death of brain endothelial cells? Our data demonstrate that M^pro^ of SARS-CoV-2 cleaves host cell NEMO with high efficiency. In infected cells, M^pro^ is located in the cytosol and nucleus, where NEMO is also present^48,49^. Cleavage by M^pro^ inactivated NEMO. This may benefit the virus by preventing the induction of antiviral type I interferons that depends on NEMO^21^. Indeed, levels of type I interferons are low or absent in the peripheral blood of patients with COVID-19 (refs. ^34,50^). Cleaving NEMO is also a strategy used by other viruses^51–53^. However, the tropism of SARS-CoV-2 is likely to limit NEMO inactivation to specific cell types. Accordingly, some NF-κB-dependent cytokines, such as TNF and IL-6, are highly upregulated, indicating that the cells of origin have escaped NEMO inactivation^34^. In addition to its central role in immunity, NEMO supports the survival of some but not all cell types^22^. While neurons, glia and endothelial cells of peripheral vessels seem to resist NEMO deficiency or are even protected by it against inflammatory stimuli^54,55^, the survival of other cells, including brain endothelial cells, is supported by NEMO. Our data suggest that, in COVID-19, brain endothelial cells are at disproportionate risk when being infected by SARS-CoV-2 because of their dependence on NEMO activity for survival.

Cleavage of NEMO by M^pro^ mimics the genetic disease *incontinentia pigmenti* that is caused by inactivating mutations in the NEMO (*IKBKG*) gene. In the latter condition, patients suffer from a mix of neurological symptoms, such as encephalopathy, stroke and seizures that resemble neurological manifestations of COVID-19 (ref. ^56^). The absence of NEMO in mice induced a loss of endothelial cells and microvascular pathology. Subsequently, patchy hypoxia developed in the brain and the BBB became leaky. In parenchymal cells, a prominent upregulation of GFAP indicated the activation of astrocytes, in line with the finding that GFAP concentrations are elevated in the blood of patients with COVID-19 (ref. ^57^). An increased BBB permeability and astrocyte activation may cause epileptic seizures in patients with COVID-19 as in *incontinentia pigmenti*^56,58^.

M^pro^-mediated damage of brain endothelial cells suggests that inhibitors of M^pro^ may prevent neurological complications of the SARS-CoV-2 infection^26^. Another therapeutic approach may arise from the observation that deletion of *Ripk3* or inhibition of RIPK1 profoundly improved the microvascular pathology. RIPK1 inhibitors are available and have already entered clinical testing^40,42^ suggesting potential therapeutic options for COVID-19 as well as for *incontinentia pigmenti*.

## Methods

### Patients

The clinical details of SARS-CoV-2-infected and control patients are summarized in Supplementary Table 1. SARS-CoV-2 infection was diagnosed by RT–PCR from pharyngeal swabs. The results of a systematic neurological examination were not available. Patients were autopsied at the University Medical Center Hamburg-Eppendorf or at the University Medical Center Göttingen. SARS-CoV-2-infected patients had partially been included in previous studies^8,9,19^. The study was approved by the local ethics committees in Hamburg and Göttingen (Hamburg approval no. PV7311; Göttingen approval no. 42/8/20). Control participants were matched to SARS-CoV-2-infected patients according to age and sex. Comorbidities did not differ between the groups, but more SARS-CoV-2-infected patients were ventilated than controls (Supplementary Table 2). Brains were fixed in buffered 4% formaldehyde, examined macroscopically and underwent routine neuropathological workup that did not show morphological signs of a global hypoxic–ischemic encephalopathy in any case (Extended Data Fig. 2j). We analyzed 3–12-µm-thick paraffin-embedded sections of the frontal lobe. The section thickness did not differ between groups (Supplementary Table 2). String vessel measurements were normalized to image volume.

For immunoblotting, frozen tissue samples of medulla oblongata from patients deceased with/from COVID-19 or controls were homogenized in cold RIPA buffer (50 mM Tris-HCl, pH 8, 150 mM NaCl, 1% NP-40, 0.5% Na-deoxycholate and 0.1% SDS) supplemented with protease and phosphatase inhibitor (cOmplete mini EDTA-free, PhosSTOP, Roche). Samples were incubated on ice for 30 min and centrifuged at 12,000*g* at 4 °C for 10 min. The resulting supernatant was mixed with Laemmli sample buffer and denatured for 10 min at 96 °C.

### Animals

#### Transgenic mouse models

Mice were housed in individually ventilated Green Line cages (Tecniplast) under a 12-h light–dark cycle and fed an autoclaved pelleted mouse diet ad libitum. We performed all studies in accordance with the German Animal Welfare Act and the corresponding regulations. Experimental procedures were approved by the local animal ethics committee (Ministerium für Landwirtschaft, Umwelt und ländliche Räume, Kiel, Germany). All mouse lines were established on a C57BL/6 background. In all experiments, adult littermate mice at the age of 6 to 24 weeks were used that were matched by age and sex between experimental groups. Unless stated otherwise, male and female mice were used.

For brain endothelial knockout of *Ikbkg* (*Nemo*) and *Fadd*, mice with the respective *loxP*-flanked alleles^60,61^ were crossed with the BAC-transgenic *Slco1c1*-CreER^T2^ strain^62^, which expresses the tamoxifen-inducible CreER^T2^ recombinase under the control of the mouse *Slco1c1* regulatory sequences in brain endothelial cells and epithelial cells of the choroid plexus. *Ripk3*^−/−^ mice have been reported previously^63^. Tamoxifen (dissolved in 90% Miglyol 812 with 10% ethanol, 50 mg per kg body weight, intraperitoneally (i.p.), twice per day for five consecutive days; Sigma-Aldrich) was injected to induce recombination. After receiving tamoxifen, *Nemo*^*fl/fl*^; *Slco1c1*-CreER^T2^ mice were indicated as *Nemo*^*beKO*^, while control littermates lacking the Cre recombinase but receiving tamoxifen injection were termed *Nemo*^*fl*^. If not mentioned otherwise, mice were perfused 14 d after receiving the first dose of tamoxifen.

For microglia depletion, mice were fed the CSF-1R inhibitor PLX5622-containing (1,200 ppm) AIN-76A rodent diet (D11100404i) 14 d before the start of tamoxifen injection. Controls received a control diet (OpenSource Diets, D10001i). Mice received pimonidazole HCl (100 mg ml^−1^ in 0.9% NaCl; Hypoxyprobe) 1 h before perfusion (i.p., 60 μg per gram of body weight).

For RIPK inhibition, a specific RIPK1 inhibitor (GSK′547, RA15777187)^64,65^ was suspended in 0.6% methyl cellulose and mice were treated with 60 mg per kg body weight every 12 h by oral gavage. For control treatment we used methyl cellulose. Treatment started 1 d before injection of viral vectors and was stopped at the day of perfusion 2 weeks after viral injection.

#### SARS-CoV-2 infection

Male 8- to 10-week-old golden Syrian hamsters and K18-hACE2-expressing C57BL/6 mice (B6.Cg-Tg(K18-hACE2)2Prlmn/J) were purchased from the Janvier Laboratory (Le Genest-St-Isle, France) and the Jackson Laboratory, respectively. The BetaCoV/France/IDF0372/2020 strain of SARS-CoV-2 was supplied by the French National Reference Center for Respiratory Viruses hosted by the Institut Pasteur (Paris, France). Hamsters were anesthetized by i.p. injection of ketamine (100 mg per kg body weight), atropine (0.75 mg per kg body weight) and diazepam (2.5 mg per kg body weight) and intranasally infected with 100 µl of DMEM containing (or not, in mock samples) 2 × 10^4^ TCID_50_ (50% tissue culture infectious dose) of SARS-CoV-2. Male 8- to 10-week-old mice were anesthetized by i.p. injection of ketamine (100 mg per kg body weight) and xylazine (10 mg per kg body weight) and intranasally infected with 50 µl of DMEM containing 5 × 10^3^ TCID_50_ of SARS-CoV-2. For brain preparation, animals were euthanized by i.p. injection of pentobarbital (140 mg per kg body weight) on day 2, 4, 7 or 24 after infection.

All experiments were performed within the biosafety level 3 suite on the Institut Pasteur de Lille campus and complied with current national and institutional regulations and ethical guidelines (Institut Pasteur de Lille/B59-350009). The protocols were approved by the institutional ethical committee (Comité d’Ethique en Experimentation Animale 75, Nord Pas-de-Calais, France) and authorized by the ‘Education, Research and Innovation Ministry’ (APAFIS no. 25041-2020040917227851; APAFIS no. 25517-2020052608325772 v3).

### Plasmid construction and AAV vector production

Plasmids (pCAG-M^pro^-HA and pCAG-p.Cys145Ala-M^pro^-HA; Vectorbuilder) contained inverted terminal repeats of AAV2, the 1,733-bp-long CAG promoter^66^, a Kozak sequence with an ATG followed by the native SARS-CoV-2 sequence encoding M^pro^ (gene ID: 43740578) or a mutated M^pro^ (codon of Cys145 (TGT) changed to Ala145 (GCT)). The 3′-end was labeled with a HA tag and a TAA codon followed by WPRE and the bovine growth hormone polyadenylation signal. We produced AAV vectors by triple transfection of HEK293T cells or Sf9 insect cells as described before^36,67,68^.

Genomic titers were determined by quantitative PCR against CAG (forward primer: 5′- AACGCCAATAGGGACTTTC-3′; reverse primer: 5′-GTAGGAAAGTCCCATAAGGTCA-3′). Vectors were injected into the tail veins of mice (1.8 or 3.3 × 10^11^ genomic particles per mouse, 100 µl). Except in the experiment using *Ripk3*^−/−^ mice (Fig. 7a,b), for which we used male and female mice, only male C57BL/6 mice were used for vector injection. Mice were perfused under deep anesthesia with PBS and paraformaldehyde (PFA, 2% or 4%) 2 weeks after administering the vector. Total DNA of a sagittal brain section (50-µm thick) was extracted using the DNeasy tissue kit (Qiagen) according to the manufacturer’s instructions. We quantified DNA with a spectral photometer (Nanodrop ND-2000C, Peqlab) as described previously^69^.

### Cell culture and transfection

#### hCMEC/D3 and bEnd.3 cells

The human brain endothelial cell line hCMEC/D3 (Merck SCC066, RRID: CVCL_U985) and the mouse brain endothelial cell line bEnd.3 (American Type Culture Collection (ATCC), CRL-2299, RRID: CVCL_0170) were cultivated as described previously^36,37^. We used 24-well plates for luciferase assays, 48-well or 96-well plates or chamber slides for immunocytochemistry and 6-well or 12-well plates for immunoblotting.

After withdrawing heparin (hCMEC/D3) or penicillin–streptomycin (bEnd.3) from the medium, we transfected the cells using Lipofectamine 3000 (Thermo Fisher Scientific) and the following plasmids: pNF-κB-Luciferase (200 ng per well; Stratagene), pCAG-hACE2-TMPRSS2 (100 ng per well on 8-well chamber slides; 2,500 ng per well on 6-well plates, Invivogen), pCAG-GFP, pCAG-p.Cys145Ala-M^pro^-HA or pCAG-M^pro^-HA (400 ng per well on 24-well and 48-well plates; 1,000 ng per well on 12-well plates), pCAG-NEMO-2A-eGFP (1,000 ng per well on 12-well plates)^36^ and pRL-SV40 (40 ng per well). The DNA was filled up with pBluescript to equal amounts per well. One day after lipofection, we treated the cells with IL-1β (0.25 µg ml^−1^; PeproTech) and measured luciferase activity using the Dual Luciferase Reporter Assay (Promega) after 6 h. Immunocytochemistry of p65 or the TUNEL reaction was performed after stimulating cells for 30 min with IL-1β (0.25 µg ml^−1^) or for 4.5 h with TNF (100 ng ml^−1^), 2–3 d after transfection. For immunoblotting, we lysed cells 2–3 d after transfection.

For infection of hCMEC/D3 cells, SARS-CoV-2 virus was isolated and propagated in Caco2 cells as previously described^70,71^. To infect endothelial cells, the viral stock was diluted to the desired MOI in culture medium supplemented with 1% FCS and incubated for 2 h with the cells 24 h after transfection. Twenty-four hours after infection, endothelial cells were fixed in 4% PFA for 10 min or lysed for immunoblotting in lysis buffer (20 mM Tris-HCl, pH 7.5, 20 mM NaF, 150 mM NaCl, 10 mM NaPPi and 1% Triton X-100).

#### Vero E6 cells

Vero E6 cells (ATCC CRL-1008) cultivated in DMEM containing 3% FCS, 1% penicillin–streptomycin, 2 mM l-glutamine, 1% sodium pyruvate and 1% non-essential amino acids (all Gibco/Thermo Fisher) were seeded in 6-well plates and infected with SARS-CoV-2 isolate HH-1 at a MOI of 1 (ref. ^72^). At the indicated time points after infection, cells were centrifuged (2,000 r.p.m., 5 min) and lysed in SDS inactivation buffer (6% SDS, 150 mM Tris, pH 6.8, 30% glycerol, 100 mM dithiothreitol (DTT) and bromophenol blue).

### Single-cell RNA sequencing

Two 10-week-old male C57BL/6 mice were killed by decapitation. The hypothalamic region was microdissected and digested with the Papain Dissociation System (Worthington, LK003150) at 37 °C. After triturating, the cell suspension was centrifuged at 700*g* for 5 min at room temperature, and the supernatant removed. We resuspended the cell pellet (500 µl EBSS, 56 µl reconstituted albumin–ovomucoid inhibitor solution and 28 µl DNase, Papain Dissociation System) and passed it through a 40-µm cell strainer. After another centrifugation step (700*g* for 5 min at room temperature), we resuspended the cell pellet in HBSS containing 5% glucose and stored it on ice.

Single-cell capture was achieved by random distribution of the single-cell suspension across >200,000 microwells through a limited dilution approach with the BD Rhapsody system. Cells were sorted as described by the manufacturer (BD Rhapsody cartridge reagent kit, 633731). In total, 20,448 viable cells were captured. Upon cDNA synthesis (BD Rhapsody cDNA kit; 633773), each cDNA molecule was tagged on the 5′ end with a molecular index and cell label indicating its cell of origin^73^. Whole-transcriptome libraries were prepared with half of the beads using the BD Resolve single-cell whole-transcriptome amplification workflow (BD targeted and AbSeq Amplification Kit, 633774) with a randomer primer pool (tcagacgtgtgctcttccgatctNNNNNNNNN). In brief, second-strand cDNA was synthesized, followed by the ligation of the adaptor for universal amplification. Eighteen cycles of PCR were used to amplify the adaptor-ligated cDNA products. Libraries were quantified using a High Sensitivity DNA chip (Agilent) on a Bioanalyzer 2100 and the Qubit High Sensitivity DNA assay (Thermo Fisher Scientific). Libraries were sequenced using High Output sequencing kits (75 × 2 bp; Illumina) by a commercial provider (Novogene).

### Bioinformatic analysis of single-cell RNA-sequencing data

Raw gene expression matrices were generated for each sample by a custom pipeline combining kallisto (v.0.46.1) and bustools (v.0.46.1) coupled with mouse reference version GRCm38. The output filtered gene expression matrices were analyzed by R software (v.4.2.0) with the DropletUtils (v.1.8.0) and Seurat (v.3.2.0) packages. In brief, for each sample, cells were detected by ranking cell barcodes according to their number of unique molecular identifiers (UMIs) captured using the barcodeRanks function. Low-ranked cells from this process were labeled as false positives and were discarded.

Only genes expressed in >0.5% of the dataset and cells with >200 genes assigned were selected for further analyses. Low-quality cells were removed if they included >20% UMIs derived from the mitochondrial genome. Gene expression matrices were normalized by the NormalizeData function and 2,000 features with high cell-to-cell variation were calculated using the FindVariableFeatures function. For both samples, we identified ‘anchors’ between individual datasets with the FindIntegrationAnchors function and fed these anchors into the IntegrateData function to create a batch-corrected expression matrix of all cells, which allowed cells from different datasets to be integrated and analyzed together. The dimensionality of the data was reduced by principal-component analysis followed by visualization with UMAP clustering using the Louvain algorithm. Finally, we clustered cells by using the FindClusters and FindNeighbors functions. Cluster-specific markers were identified by the FindAllMarkers function and assigned to cell types. Clusters were then classified and annotated based on expressions of canonical markers of particular cell types. All details regarding the Seurat analyses performed in this work can be found in the website tutorial (https://satijalab.org/seurat/v3.2/pbmc3k_tutorial.html).

For analysis of single-nuclei RNA-seq data from human brain, preprocessed expression matrices were obtained from the Gene Expression Omnibus (GEO; GSE97942) consisting of >60,000 single nuclei from the human adult visual cortex, frontal cortex and cerebellum^28^. The gene expression matrices were further processed as described above using the Seurat package (v.3.2.0).

### Proteolytic cleavage of NEMO

The M^pro^ protein was generated as described recently^20^. The purified protein was stored at −80 °C in protease buffer (20 mM Tris, 150 mM NaCl, 1 mM EDTA, 1 mM DTT, pH 7.8) until usage.

For immunoblotting, recombinant human NEMO with an N-terminal GST tag (4.1 µg, ab206008, Abcam) was incubated with M^pro^ at the indicated concentrations and time points in protease buffer. For mass spectrometry, NEMO was incubated with M^pro^ (5 µM) in protease buffer for 3 h at 37 °C and samples were lyophilized. Tryptic in-solution digestion was performed as described previously^74^. Briefly, after resuspending samples in 50 µl 6 M urea, a reduction was performed using 2.5 mM DTT (in 100 mM NH_4_HCO_3_) at 56 °C for 20 min and samples were alkylated using 7.4 mM iodoacetamide (in 100 mM NH_4_HCO_3_) for 30 min at room temperature in the dark. For tryptic digestion, first NH_4_HCO_3_ (425 µl, 100 mM) and then trypsin solution (sequencing grade, 1.5 µl, 0.05 µg µl^−1^ in 50 mM acetic acid, Promega) were added. After incubation for 18 h at 37 °C, samples were desalted using C18 SPE cartridges (Sep-Pak, Waters) and resuspended in 30 µl 0.1% formic acid for liquid chromatography (LC)–MS/MS.

To validate the cleavage of human and mouse NEMO at Q231, synthetic peptide substrates as shown in the respective figures and reference h-NEMO_222-231 (EEKRKLAQLQ), consisting of the native or mutated human or mouse NEMO sequence, were commercially obtained (Peptide Specialty Laboratories). Peptide substrates (10 µM) were incubated with M^pro^ (2.5 µM) for 1 h at 37 °C in water. We precipitated proteins using ice-cold acetonitrile and then kept samples at −20 °C for 10 min followed by centrifugation at 4 °C and 20,817*g* for 10 min. The supernatant was lyophilized and samples were dissolved in 30 µl 0.1% formic acid and further diluted at a 1:1 ratio with 0.1% formic acid for LC–MS/MS. To determine the apparent catalytic efficiency, M^pro^ (2.5 µM) was incubated with different concentrations of h-NEMO_222-241 for 30 min at 37 °C in protease buffer and samples were processed as described above.

### Immunoblotting

Samples were supplemented 1:4 with SDS buffer (0.75 M Tris-HCl, 0.08 g ml^−1^ SDS, 40% glycerol, 0.4 mg ml^−1^ bromophenol blue and 62 mg ml^−1^ DTT) and incubated at 95 °C for 10 min. After loading on SDS–PAGE gels, we transferred proteins to nitrocellulose membranes, which were incubated with primary antibodies (Supplementary Table 3) overnight at 4 °C. Subsequently, we incubated membranes with HRP‐conjugated secondary antibodies (Supplementary Table 4) for 2 h at room temperature. For detection, we used enhanced chemiluminescence (SuperSignal West Femto Substrate, Thermo Scientific) and a digital detection system (Fusion Solo S, Vilber). Immunoblots of IgG and albumin were analyzed by ImageJ (National Institutes of Health, RRID: SCR_002285). The intensity of the target protein was expressed relative to the intensity of actin and normalized to the ratio of the control group (*Nemo*^*fl*^).

### Dextran extravasation

Dextran (4 kDa, labeled with FITC; BD) was suspended in PBS (12 mg ml^−1^) and intravenously injected (100 µl per mouse) 30 min before perfusion. Brains were homogenized as described previously^37^. In supernatants, fluorescence was detected using a microplate reader (CLARIOstar, BMG LABTECH).

### Mass spectrometry

Analyses were performed on a UHPLC system (Dionex Ultimate 3000, Thermo Scientific) coupled to a quadrupole orbitrap mass spectrometer (Orbitrap Q-Exactive, Thermo Scientific). We separated the samples on a RP separation column (ACQUITY UPLC BEH C18, 130 Å, 1.7 µm, 2.1 × 100 mm, Waters) using H_2_O and acetonitrile (LC–MS grade, Merck KGaA), both containing formic acid (0.1%), as eluents at a flow rate of 200 µl min^−1^. We used stepwise and linear gradients: For tryptic NEMO peptides, 3–45% B in 43 min, 45–70% B in 7 min; for synthetic NEMO peptides, 3–70% B in 10 min. All spectra were acquired in positive-ion mode and capillary voltage was set to 3,500 V, capillary temperature to 320 °C, sheath gas flow to 30 and auxiliary gas flow to 10. Full scan spectra were acquired with a scan range of 150–2,000 *m/z* (tryptic NEMO peptides) or 400–2,000 *m/z* (synthetic NEMO peptides) and the resolution was set to 70,000, AGC target to 3 × 10^6^ and maximum injection time to 100 ms. For precursor selection, ions of charge states of 1+ and >6+ were excluded from fragmentation. We fragmented precursor ions using higher-energy C-trap dissociation with a stepped normalized collision energy of 30. MS/MS spectra were acquired with a resolution of 17,500, AGC target was set to 10^5^ and the maximum injection time to 50 ms. Data analysis was performed using the Xcalibur software (version 3.0.63, Thermo Scientific). To identify NEMO-derived peptides, a database search using the MaxQuant software (version 1.6.1.0, Max Planck Society)^75^ with the Andromeda search engine was performed. We searched raw data against a human NEMO database (https://www.uniprot.org/; July 2020). Digestion mode was set to unspecific and minimum peptide length to 3.

### RNAscope (in situ hybridization) for detecting SARS-CoV-2 in human samples

Human brains were immersion fixed in 10% formalin for 1 week at room temperature followed by 4% PFA and PBS 0.1 M (pH 7.4) for an additional 48 h at 4 °C, cryoprotected in 30% sucrose for one additional week at 4 °C, Tissue-tek embedded and frozen in liquid nitrogen at the crystallization temperature of isopentane. The SARS-CoV-2 S gene encoding the spike protein was detected on 20-µm-thick sections using RNAscope Multiplex Fluorescent Reagent kit v2 Assay and the V-nCov2019-S probe (848561, both Advanced Cell Diagnostics) according to the manufacturer’s instructions.

### Immunofluorescence staining and confocal microscopy

Human brain sections were deparaffinized in xylene and ethanol, rehydrated in water and rinsed in 0.1% Triton X-100 in PBS for 10 min and 0.1% Tween-20 in PBS for 5 min. To retrieve antigens, we incubated the sections in sodium citrate buffer (10 mM, pH 6, 95 °C, 10 min). The sections were blocked in PBS containing 5% BSA and 0.1% Triton X-100 for 30 min. Primary antibodies (Supplementary Table 3) diluted in blocking solution were incubated at 4 °C overnight. Secondary antibodies (Supplementary Table 4) diluted in blocking solution were incubated at room temperature for 1 h in the dark.

For the staining of cryosections of mouse brains, we perfused mice under deep anesthesia with PBS containing heparin (10 IU ml^−1^). Brains were frozen on dry ice and stored at −80 °C. Sections (20-µm thick) were postfixed in methanol for 10 min at −20 °C or in 4% PFA in PBS for 15–20 min at room temperature, if not indicated otherwise. Specimens were blocked with either 1–3% BSA in PBS (methanol post-fixation) or 1–3% BSA and 0.1–0.3% Triton X-100 in PBS (PFA post-fixation) for 1 h and stained as described for human sections.

For vibratome sections, mice were either not perfused or perfused with freshly prepared PFA (2% in PBS, 4% for GFP staining). Brains were postfixed in 2% PFA for 7 h (4% PFA for 2 h for GFP staining) at 4 °C before sectioning using a vibratome (Leica, VT1200S). Before the staining of brain sections from SARS-CoV-2-infected hamsters or mice, pepsin antigen retrieval was performed for 10 min at 37 °C (0.1 mg ml^−1^ pepsin in PBS, 0.2 N HCl). Sections (50- or 100-µm thick) were blocked with 3% BSA in PBS containing 0.1–0.3% Triton X-100 for 6 h at room temperature, and incubation with primary antibodies (Supplementary Table 3) was performed at 4 °C for 48–72 h, while incubation with secondary antibodies (Supplementary Table 4) was performed in blocking solution at 4 °C overnight.

For the TUNEL assay, mouse brains were postfixed in 4% PFA in PBS at 4 °C overnight and transferred to a 30% sucrose solution the next day. On the following day, brains were frozen and stored at −80 °C. Cryosections (40-µm thick) were prepared and a heat-induced epitope retrieval was performed using 10 mM sodium citrate buffer at 95 °C for 20 min. TUNEL assay was applied after immunohistochemistry staining according to the manufacturer’s instructions (In Situ Cell Death Detection Kit, Fluorescein; Roche, 11684795910).

Images were taken by confocal laser scanning microscopes (Leica, SP5 or SP8) or a fluorescence microscope (Leica, DMI 6000B). Images for determining the number of string vessels in human samples as well as super-resolution images were taken using a STED microscope, custom made by Abberior Instruments. All images were produced using the same setting.

For all analyses, we imaged four fields from two sections per individual unless stated otherwise.

### Non-fluorescence histological staining on human samples

Histological hematoxylin and eosin staining and Nissl staining were performed as described before^19^. For active caspase-3 staining, deparaffinized tissue sections were treated for antigen retrieval as described above and subsequently with 3% H_2_O_2_ before blocking with PBS containing 10% FCS. Primary antibodies were applied overnight and visualized using the EnVision+ System for rabbit and mouse (Dako). We briefly counterstained sections with hemalaun. To evaluate the number of active caspase-3-positive cells, sections were scanned (magnification of ×200) using the Virtual Slide Microscope VS120. Image visualization and manual analysis were performed using Omero Server software (5.6.3)^76^.

### Super-resolution microscopy

We used stimulated emission depletion (STED) imaging and expansion microscopy. For STED, 640- and 561-nm diode excitation lasers, a 775-nm STED laser, all pulsed at 40 MHz, and a ×100 1.4-NA Olympus UPlanSApo were utilized. A spatial light modulator (Hamamatsu) was used to produce either a doughnut-shaped (two-dimensional (2D) STED) or a top-hat (three-dimensional (3D) STED) phase mask, shaping different depletion beams without changing the optical setup. To reduce photobleaching at an optimal signal-to-noise ratio, we used DyMIN adaptive illumination.

For expansion microscopy, after immunofluorescence staining, gelation, digestion and expansion were performed as described previously^77^. Notably, we extended incubation time in monomer solution to 45 min and gelation time to 2.5 h, and digestion was performed overnight. Images were taken with an HC PL APO CS2 ×40/1.10 water objective. Expansion microscopy was used for qualitative representation of occludin and ZO-1 morphology.

### Quantitative analysis of immunostainings

To analyze IgG transcytosis, *z*-stacks were taken with an HCX PL APO CS ×63/1.4 oil objective for confocal microscopy or a ×100 1.4-NA Olympus UPlanSApo for STED microscopy. Deconvolution was performed for confocal images by Huygens Software (Scientific Volume Imaging). IgG vesicle quantification was performed using Imaris 9.3.0 (Bitplane) as described before^41^. The 3D vasculature mask was smoothed under surface details (1.0 µm confocal/0.4 µm for STED), and spots were identified with an estimated diameter of 0.5 µm. IgG^+^ vesicles and IgG extravasation were quantified on 10–15 images on one section for each animal (confocal) or three images for each animal (STED).

String vessels and their localization in the vascular tree, vessel length, vessel diameter, occludin interruptions and GFAP^+^ astrocytes were analyzed using ImageJ. Mouse and hamster string vessels were analyzed as before^37^.

For vascular tree analysis, string vessels were counted manually and tracked in tile scans of the cortex, hippocampus and hypothalamus (one section per animal). As the starting point of the vascular tree, we chose α-SMA^+^ vessels, indicating arterioles. The number of string vessels was normalized to the total area of the image.

Because CD34 staining was unreliable in human brain sections, human string vessels were identified as collagen IV-positive tubes of <4 µm in apparent outer diameter in images produced by confocal laser scanning microscopy. Because the theoretical minimal diameter for a functional capillary is 2.7 µm and more than 90% of brain capillaries in aged humans have an inner diameter of >3.5 µm, we expect this threshold to be selective for string vessels^78,79^.

Vessel diameter was measured by using the DiameterJ plugin for ImageJ. For the TUNEL analysis, collagen IV^+^ and TUNEL^+^ vessels were counted and normalized to the total image area.

GFAP-positive astrocytes were quantified as the percentage of the GFAP^+^ area relative to the total area of the image. Quantifications were obtained from 4–6 images per animal (one section per animal). Images were taken from the cortex if not stated otherwise.

Pericyte coverage was analyzed using MotionTracking software (MPI-CBG, v8). In general, the CD13^+^ area inside the collagen IV^+^ vessels was normalized to the total collagen IV^+^ area. For measuring occludin interruptions, the lengths of occludin and ZO-1 tight junctions were traced manually by using the Simple Neurite Tracer plugin (ImageJ). Then, occludin length was normalized to ZO-1 length for 15 images per animal (one section per animal).

For the soma size measurement of Iba1^+^ cells, confocal imaging was obtained with 25 steps and a step size of 0.99 µm. Maximal-intensity *z*-stacks were generated using ImageJ. A threshold was applied (Li autothreshold), converted to a mask and speckles and outliers removed. Then, all the processes were removed from the soma and the soma area was measured for 15 microglia per sample.

### Electron microscopy

For electron microscopy, mice were perfused with heparinized Ringer’s solution and with freshly prepared PFA and glutaraldehyde (2.5% glutaraldehyde and 2% PFA in PBS). Until further processing, brains were postfixed in Monti-Graziadei solution (2% glutaraldehyde, 0.6% PFA, 0.03% calcium chloride in 0.06 M sodium cacodylate buffer, pH 7.35) for at least 48 h at 4 °C. After further fixation in 1% osmium tetroxide in 0.1 M cacodylate buffer for 2 h, samples (approximately 1 mm^3^) were dehydrated in an ascending series of ethanol and incubated in propylene oxide followed by a 1:1 mixture of propylene oxide and araldite (Sigma-Aldrich) and subsequently embedded in araldite. Ultrathin sections were cut at approximately 80 nm and were transferred to copper grids. Sections were contrasted in a contrasting system for ultrathin sections using uranyl acetate ready-to-use solution, followed by lead citrate ready-to-use solution (all Leica Microsystems). Images of vessels smaller than 10 µm were taken by an electron microscope (Jeol JEM 1011). After putting images of a vessel into a collage by using Inkscape 1.0.1 (RRID: SCR_014479), vesicles in the range of 30–200 nm were manually counted from three capillaries for each animal, and luminal membrane length was measured using ImageJ.

### Statistics and reproducibility

No statistical methods were used to predetermine sample sizes, but our sample sizes are similar to those reported in previous publications^37,41,80^. Data were analyzed using Prism 8 (GraphPad) and SPSS 25 (IBM). Significance was considered when *P* < 0.05. Depending on the dataset and experimental design, different statistical methods were used as indicated in Supplementary Table 5. Parametric statistics (for example, *t*-test and ANOVA) were only applied if assumptions were met, that is, datasets were examined for Gaussian distribution using the D’Agostino–Pearson test, aided by visual inspection of the data and homogeneity of variances by Brown–Forsythe, Levene’s or *F*-test (depending on the statistical method used). If assumptions for parametric procedures were not met or could not be reliably assumed due to small sample size, non-parametric methods were used as indicated. Two-tailed tests were applied if not indicated otherwise. Greenhouse–Geisser correction was used in ANOVA statistics if the sphericity assumption was violated (Mauchly test). No data points were excluded. Cell culture studies were performed at least three times in independent experiments with at least three replicates per condition and per experiment unless stated otherwise. Animal experiments were repeated as stated by the *N* number. Animals were randomly allocated to diet or treatment groups as long as age-matched, sex-matched and littermate conditions were fulfilled. All analyses were performed blinded without the knowledge of the genotype, treatment or infection status if not needed for subsequent processing.

### Reporting Summary

Further information on research design is available in the Nature Research Reporting Summary linked to this article.

## Online content

Any methods, additional references, Nature Research reporting summaries, source data, extended data, supplementary information, acknowledgements, peer review information; details of author contributions and competing interests; and statements of data and code availability are available at 10.1038/s41593-021-00926-1.

## Supplementary information

Supplementary Information Supplementary Tables 1–5 and Supplementary Figs. 1–7

Reporting Summary

## Data Availability

The data that support the findings of this study are available from the authors on reasonable request. RNA-seq data are available at the GEO under accession GSE180984. Source data are provided with this paper.

## Source data

Source Data Fig. 3 Unprocessed western blots.
Source Data Fig. 4 Unprocessed western blots.
Source Data Fig. 6 Unprocessed western blots.
Source Data Extended Data Fig. 5 Unprocessed western blots.
Source Data Extended Data Fig. 6 Unprocessed western blots.
Source Data Extended Data Fig. 7 Unprocessed western blots.
Source Data Extended Data Fig. 9 Unprocessed western blots.
