# Supplementary material for: The SARS-CoV-2 main protease Mpro causes microvascular brain pathology by cleaving NEMO in brain endothelial cells
Source: Nat Neurosci. 2021 Oct 21;24(11):1522–33. doi: 10.1038/s41593-021-00926-1 (PMC8553622; doi:10.1038/s41593-021-00926-1)
Supplement: Supplementary file 1 — Supplementary Tables 1–5 and Supplementary Figs. 1–7 [file 41593_2021_926_MOESM1_ESM.pdf]

nature portfolio

https://doi.org/10.1038/s41593-021-00926-1

Supplementary information

The SARS-CoV-2 main protease M^pro causes microvascular brain pathology by cleaving NEMO in brain endothelial cells

In the format provided by the authors and unedited

The SARS-CoV-2 main protease M^{pro} causes microvascular brain pathology by cleaving NEMO in brain endothelial cells

Jan Wenzel*, Josephine Lampe*, Helge Müller-Fielitz*, Raphael Schuster, Marietta Zille, Kristin Müller, Markus Krohn, Jakob Körbelin, Linlin Zhang, Ümit Özorhan, Vanessa Neve, Julian U. G. Wagner, Denisa Bojkova, Mariana Shumliakivska, Yun Jiang, Anke Fähnrich, Fabian Ott, Valentin Sencio, Cyril Robil, Susanne Pfefferle, Florent Sauve, Caio Coelho, Jonas Franz, Frauke Spiecker, Beate Lembrich, Sonja Binder, Nina Feller, Peter König, Hauke Busch, Ludovic Collin, Roberto Villaseñor, Olaf Jöhren, Hermann C. Altmeppen, Manolis Pasparakis, Stefanie Dimmeler, Jindrich Cinatl, Klaus Püschel, Matija Zelic, Dimitry Ofengeim, Christine Stadelmann, François Trottein, Ruben Nogueiras, Rolf Hilgenfeld, Markus Glatzel, Vincent Prevot, Markus Schwaninger

* contributed equally

Supplementary data

Supplementary Table 1: Clinical characteristics of patients. Sex: m, male; f, female. Place of death: ER, emergency room; hosp., hospital ward; ICU, intensive care unit; NH, nursing home. PMI, postmortem interval. Ventilated: N, no; Y, yes; if known, including duration of ventilation. Medical history: AML, acute myeloid leukemia; CI, cardiac insufficiency; COPD, chronic obstructive pulmonary disease; DM, diabetes mellitus; IHD, ischemic heart disease; RI, renal insufficiency. For brain edema, brain atrophy, arteriosclerosis, the following score was used: 0: none; 1: basal, 2: moderate; 3: severe. Macroscopic findings: AD, Alzheimer disease; MCA, middle cerebral artery; PCA, posterior cerebral artery; PICA, posterior inferior cerebellar artery; ARDS, Acute respiratory distress syndrome.

ID	Sex	Age [y]	Place of death	PMI (days)	Cause of death	Ventilated	Medical history	Brain weight [g]	Brain edema	Brain atrophy	Arterio-sclerosis	Routine neuropathological findings	Classification of comorbidities
**COVID-19**													
1	f	87	NH	0	pneumonia	N	COPD, dementia, IHD, RI	1215	0	1	2	none	cerebral, cardiovascular, respiratory, renal insufficiency
2	f	85	hosp.	0	pneumonia	Y	atrial fibrillation, CI, IHD, myelofibrosis, RI	1240	0	1	2	fresh infarction in territory of the PCA	cerebral, cardiovascular, renal insufficiency
3	m	90	NH	2	pneumonia	N	atrial fibrillation, dementia, DM, history of stroke	1015	0	2	3	old infarctions in territory of the PCA	cerebral, cardiovascular, diabetes
4	m	76	ICU	3	sepsis, aortic valve endocarditis, pneumonia	Y	AML, cardiomyopathy, thyroid cancer	1270	0	1	1	none	cardiovascular, cancer
5	m	70	hosp.	1	pneumonia (aspiration)	Y	CI, COPD, IHD, Parkinson's disease	1430	1	0	3	none	cerebral, cardiovascular, respiratory
6	f	87	home	1	pneumonia	Y	CI, COPD	1180	0	1	3	none	cardiovascular, respiratory

ID	Sex	Age [y]	Place of death	PMI (days)	Cause of death	Ventilated	Medical history	Brain weight [g]	Brain edema	Brain atrophy	Arterio-sclerosis	Routine neuropathological findings	Classification of comorbidities
7	m	71	hosp.	2	pneumonia	Y	Lung disease, CI	1665	2	0	2	none	cardiovascular, respiratory
8	m	63	ICU	1	lung embolism	Y	CI	1435	0	0	1	none	cardiovascular
9	m	94	hosp.	2	pneumonia	Y	CI, DM	1000	0	1	1	old infarctions in territory of the PICA	cerebral, cardiovascular, diabetes
10	f	75	hosp.	4	pneumonia	Y	CI	1210	0	1	3	old infarctions in territory of the PICA	cerebral, cardiovascular
11	f	70	hosp	4	pneumonia	Y	CI, DM, renal insufficiency	1150	0	1	2	none	cardiovascular, diabetes, renal insufficiency
12	f	72	hosp.	1	pneumonia	Y (>1d)	None	1303	0	0	2	none	cardiovascular
13	f	98	hosp.	4	pneumonia	Y (4d)	arterial hypertension, atrial fibrillation, bradycardia, left MCA stroke	1080	0	2	2	old infarctions in territory of the MCA	cerebral, cardiovascular
14	m	80	hosp.	3	pneumonia	Y (24d)	arterial hypertension, atrial fibrillation, 2014, Stage 4 chronic kidney disease endocarditis, granulomatosis with polyangiitis, anemia, polyneuropathy, osteoporosis	1137	0	1	2	none	cardiovascular
15	m	68	hosp.	1	pneumonia	Y (17d)	None	1449	0	0	0	none	none
16	m	68	hosp.	1	sepsis, multiple organ failure	Y (4d)	Pancreatitis	1524	0	0	1	none	cardiovascular
17	m	71	hosp.	0.5	pneumonia	Y	arterial hypertension, Parkinson’s disease	1559	0	0	1	none	cerebral, cardiovascular

ID	Sex	Age [y]	Place of death	PMI (days)	Cause of death	Ventilated	Medical history	Brain weight [g]	Brain edema	Brain atrophy	Arterio-sclerosis	Routine neuropathological findings	Classification of comorbidities
**Controls**													
1	f	87	hosp.	3	myocardial infarction	N	cardiomyopathy	1270	1	0	1	none	cardiovascular
2	f	86	hosp.	4	cardiac failure	N	DM, IHD	1538	2	0	3	none	cardiovascular, diabetes
3	f	86	hosp.	6	cardiac failure	N	cardiomyopathy	1180	1	1	1	none	cardiovascular
4	m	76	hosp.	3	pneumonia	no info	myocardial infarction	1250	0	1	1	none	cardiovascular
5	m	70	hosp.	3	myocardial infarction	no info	CI, liver cirrhosis	1301	1	0	2	none	cardiovascular
6	m	88	hosp.	3	cardiac failure	no info	CI, cardiomyopathy	1420	1	0	1	none	cardiovascular
7	m	71	hosp.	5	hemorrhagic shock	N	pancreatic carcinoma	1510	1	0	1	none	cardiovascular, cancer
8	f	71	hosp.	4	lung embolism	N	CI	1276	0	0	2	none	cardiovascular
9	m	62	hosp.	4	cardiac failure	Y	CI, RI	1270	0	0	0	none	cardiovascular, renal insufficiency
10	f	70	hosp	3	multiple organ failure	N	CI, RI	1130	0	0	1	none	cardiovascular, renal insufficiency
11	m	74	hosp	3	pneumonia	Y	CI	1348	0	0	1	none	cardiovascular
12	m	85	hosp	1	circulatory arrest	Y (<1d)	RI	1220	0	0	1	none	cardiovascular, renal insufficiency
13	m	62	hosp	5	circulatory arrest	Y (<1d)	cancer (rectum, bladder, prostate, colon)	1400	0	0	1	old infarctions in basal ganglia and pons	cerebral, cardiovascular, cancer
14	f	86	hosp	2	circulatory arrest	N	hiatus hernia, gastroesophageal reflux disease	1171	0	0	1	fresh infarction	cerebral, cardiovascular

ID	Sex	Age [y]	Place of death	PMI (days)	Cause of death	Ventilated	Medical history	Brain weight [g]	Brain edema	Brain atrophy	Arterio-sclerosis	Routine neuropathological findings	Classification of comorbidities
15	m	79	hosp	1	cardiac failure	Y (13d)	surgery for a penetrating aortic ulceration with post-operative complications including mesenteric ischemia, hemicolectomy, pneumonia, and cardiac arrest	1460	0	0	3	none	cardiovascular, respiratory
16	m	60	hosp	4	sepsis, pneumonia	Y (20d)	None	1300	0	0	1	none	cardiovascular
17	f	64	hosp	1	circulatory arrest	Y (25d)	None	1230	1	0	1	old intracerebral hemorrhage, fresh subarachnoid hemorrhage	cerebral, cardiovascular
18	f	70	hosp	2	pneumonia	Y	CI, RI, cardiac cirrhosis, sepsis, suspected H1N1 infection	1258	0	0	1	AD pathology	cerebral, cardiovascular, renal insufficiency
19	m	89	hosp	3	cardiac failure	N	multiple bleedings, increased tendency to bleed, prostate hyperplasia, RI, anemia, CI, monoclonal gammopathy, sleep apnea, DM, hyperlipoproteinemia, resection of the stomach, ulcus and spleen in 1984, myelodysplastic syndrome suspected	1303	0	0	2	none	cardiovascular, diabetes, renal insufficiency, cancer

ID	Sex	Age [y]	Place of death	PMI (days)	Cause of death	Ventilated	Medical history	Brain weight [g]	Brain edema	Brain atrophy	Arterio-sclerosis	Routine neuropathological findings	Classification of comorbidities
20	f	75	hosp	7	multi organ failure	N	CI, COPD	1336	0	0	1	none	cardiovascular, respiratory
21	m	66	hosp	2	pneumonia	Y (>4d)	Arthritis	1390	0	0	1	none	cardiovascular
22	m	64	hosp	4	ARDS	Y (24d)	cardiomyopathy, CI, pulmonary hypertension, coronary heart disease, DM, RI, pneumonia, squamous cell carcinoma, hypoxemia	1486	0	0	2	none	cardiovascular, respiratory, diabetes, renal insufficiency, cancer
23	f	81	hosp	3	aorto-esophageal fistel, bleeding	N	aortic aneurysm	1215	0	0	1	none	cardiovascular

Supplementary Table 2: Characteristics of control and COVID-19 patients. Mann Whitney U test was performed to compare age and the thickness of frontal brain sections that were used in Fig. 1. Fisher's exact test was used for sex, comorbidities, treatment in an intensive care unit, and ventilation. IQR, interquartile range. $^{+}$information not available for all patients, $^{\#}$the duration of ventilation was only available for part of the patients (control: 7, COVID-19: 5).

	**Control**	**COVID-19**	**Statistics**
N	23	17	
Age, median (IQR)	74 (66-86)	75 (70-87)	p = 0.373
Sex, female/male	10/13	7/10	p = 1.000
Section thickness [µm], mean ± SEM	7.3 ± 0.5	6.8 ± 0.7	p = 0.464
Intensive care unit	16/22^{+}	15/17	p = 0.426
Artificial ventilation	10/20^{+}	14/16^{+}	p = 0.032
Duration of ventilation$^{\#}$, mean ± SEM	12.6 ± 4.0	10.2 ± 4.4	p = 0.900
**Comorbidities**			
Cerebral	4/23	8/17	p = 0.079
Cardiovascular	23/23	16/17	p = 0.425
Respiratory	3/23	4/17	p = 0.432
Diabetes	3/23	3/17	p = 1.000
Renal insufficiency	6/23	3/17	p = 0.707
Cancer	4/23	1/17	p = 0.373

Supplementary Table 3: List of primary antibodies, IF: immunofluorescence on cryosections (cryo), fixed cells (cells), or fixed vibratome sections (vibratome); IHC-P: immunohistochemistry on human paraffin sections; IB: immunoblotting.

Target	Company (catalogue no.)	Dilution (final concentration)	RRID	Usage	Validation
2A	Millipore (ABS31)	1:1000	AB_10615498	IB	Fig. 2c
ACE2	R&D Systems (AF3437)	1:100 (2 µg/ml)	AB_2223140	IF, vibratome	Specific in IB according to manufacturer
ACE2	R&D Systems (AF933)	1:50 (4 µg/ml)	AB_355722	IF, cryo	Used in numerous studies according to manufacturer
Actin	Millipore (MAB1501)	1:5000	AB_2223041	IB	'highly published and validated' according to manufacturer, used in numerous studies
Albumin	Bethyl (A90-134)	1:16,000	AB_67120	IB	Fig. 6c; mentioned in 9 publications according to manufacturer
Caveolin-1	Cell Signaling Technology (3267)	1:400	AB_2275453	IF, vibratome	Monoclonal rabbit antibody, specific in IB according to manufacturer
CD13	R&D Systems (AF2335)	1:200 (1 µg/ml)	AB_2227288	IF, vibratome	Grubb, S., *et al.* Precapillary sphincters maintain perfusion in the cerebral cortex. *Nat Commun* **11**(2020)
CD147	Abcam (ab34016)	1:200 (5 µg/ml)	AB_726136	IF, vibratome	Hanna, S.M., *et al.* A novel form of the membrane protein CD147 that contains an extra Ig-like domain and interacts homophilically. *BMC Biochemistry* **4**, 17 (2003).
CD31	BD-Pharmingen (553370)	1:500 (1.0 µg/ml)	AB_394816	IF, cryo	Monoclonal antibody used by numerous studies according to manufacturer, validated by the staining pattern
CD31	Bio-Rad (MCA2388)	1:200 (5 µg/ml)	AB_2161024	IF, vibratome	Monoclonal antibody used by numerous studies according to manufacturer, validated by the staining pattern
CD34	Leica (NCL-L-END)	1:50 (5.2 µg/ml)	AB_563552	IHC-P	Monoclonal antibody used by numerous studies according to manufacturer, validated by the staining pattern
Cleaved Caspase-3	BD Biosciences (559565; C92-605)	1:150 (3.3 µg/ml)	AB_397274	IHC-P	Used by numerous studies according to manufacturer, validated by the staining pattern
Cleaved Caspase-3	Cell Signaling Technology (9664S)	1:400	AB_2070042	IF, vibratome	Monoclonal antibody validated by IB, used in numerous studies according to manufacturer
Collagen IV	Abcam (ab6586)	1:1000	AB_305584	IF, cryo	Used by numerous publications according to manufacturer, validated by the staining pattern
Collagen IV	Bio-Rad (134001)	1:200 (2 µg/ml)	AB_2082646	IF, cryo, IHC-P, vibratome,	Used by 4 publications according to manufacturer, validated by the staining pattern
Double-stranded RNA (dsRNA)	SINCONS J2 (1001050)	1:500	AB_2651015	IF, paraffin	Used in > 600 publications
ERG	Abcam (ab92513)	1:200 (4.4 µg/ml)	AB_2630401	IF, cryo	Monoclonal antibody, validated by IB, used by numerous studies according to manufacturer
GFAP	Dako (Z033429-2)	1:1000 (3.2 µg/ml)	Not available	IF, vibratome	Validated by cell morphology (Fig. 5k)
GFP	Abcam (ab13970)	1:2000 (5 µg/ml)	AB_300798	IF, vibratome, cells	Used by numerous publications according to manufacturer
HA-tag	Santa Cruz Biotechnology (sc-805)	1:500 (0.4µg/ml)	AB_631618	IB	Fig. 3c
HA-tag	Cell Signaling Technology (3724)	1:1000	AB_1549585	IF	Monoclonal antibody validated by IB and IF, used in numerous studies according to manufacturer
Iba1	Wako (019-19741)	1:400	AB_839504	IF, vibratome	Validated by cell morphology (Fig. 5j)
Iba1	Abcam (ab5076)	1:800 (0.63 µg/ml)	AB_2224402	IF, vibratome	Validated by cell morphology (Fig. 5j)

Ki67	Abcam (ab16667)	1:200 (5.77 µg/ml)	AB_302459	IF, vibratome	Knockout validated according to manufacturer
NEMO	Santa Cruz Biotechnology (sc-8330)	1:500	AB_2124846	IB	Fig. 3
NEMO	Abcam (ab244244)	1:500 (0.4 µg/ml)	Not available	IB	Fig. 3
Neuropilin-1	R&D Systems (AF566)	1:100 (2 µg/ml)	AB_355445	IF, vibratome	Validated by IB and used by numerous studies according to manufacturer
Neuropilin-1	Abcam (ab81321)	1:250 (1.55 µg/ml)	AB_1640739	IF, cryo	Monoclonal antibody validated by IB and IF according to manufacturer
M^{pro} (Nsp5)	Novus Biologicals (NBP1-78110)	1:10.000 (0.5 µg/ml)	AB_11014870	IB	Fig. 3b
Occludin	Thermo Fisher Scientific (71-1500)	1:200 (1.25 µg/ml)	AB_2533977	IF, vibratome	Validated by IB according to manufacturer and by staining pattern (Fig. 6e)
p65	Santa Cruz Biotechnology (sc-8008)	1:200 (2 µg/ml)	AB_628017	IF, cells	Validated by IB according to manufacturer and by nuclear translocation in response to IL-1β (Fig. 4a)
PDGFRβ	Cell Signaling Technology (3169)	1:100	AB_2162497	IF, vibratome	Monoclonal antibody validated by IB according to manufacturer
Pimonidazole	Hypoxyprobe Inc. (PAb2627AP)	1:500 (1 µg/ml)	Not available	IF, vibratome	Validated by the lack of staining in vehicle-treated animals
SARS-CoV-2 (2019-nCoV) Spike S1	Sino Biological (40150-R007-100)	1:1500	Not available	IF, cells	Monoclonal antibody, validated in ACE2-overexpressed 293T cells, infected or noninfected by 2019-nCOV-Spike pseudovirus (PSV) according to the manufacturer
Smooth muscle actin	Millipore (C6198-100UL)	1:100 (15 µg/ml)	AB_476856	IF, vibratome	Skalli, O., *et al.* A monoclonal antibody against alpha-smooth muscle actin: a new probe for smooth muscle differentiation. *J Cell Biol* **103**, 2787-2796 (1986).
ZO-1	Thermo Fisher Scientific (14-9776-82)	1:200 (2.5 µg/ml)	AB_2573026	IF, vibratome	Validated by knockdown experiments according to manufacturer

Supplementary Table 4: List of secondary antibodies, IF: immunofluorescence; IHC-P: immunohistochemistry on human paraffin sections; STED: stimulated emission depletion microscopy; IB: immunoblotting.

**Target (coupled to)**	**Company (catalogue no.)**	**Dilution (final concentration)**	**RRID**	**Usage**
chicken IgG (Alexa Fluor 488)	Jackson ImmunoResearch Labs (703-545-155)	1:400 (3.75 µg/ml)	AB_2340375	IF
goat IgG (Alexa Fluor 488)	Thermo Fisher Scientific (A-11055)	1:400 (5 µg/ml)	AB_2534102	IF, STED
goat IgG (Alexa Fluor 647)	Thermo Fisher Scientific (A-21447)	1:400 (5 µg/ml)	AB_2535864	IF, IHC-P
goat IgG (Cy3)	Jackson ImmunoResearch Labs (705-165-147)	1:400 (3.75 µg/ml)	AB_2307351	IF
goat IgG (HRP)	DakoCytomati (P0160)	1:2000	Not available	IB
goat IgG (StarRed)	Abberior (STRED-1055-500UG)	1:250	AB_2861383	IF, STED
mouse IgG (Alexa Fluor 488)	Thermo Fisher Scientific (A-21202)	1:400 (5 µg/ml)	AB_141607	IHC-P
mouse IgG (Alexa Fluor 594)	Thermo Fisher Scientific (A-21203)	1:100 (20 µg/ml)	AB_2535789	IF, STED
mouse IGG (Cy3)	Jackson ImmunoResearch Labs (715-165-151)	1:200	AB_2315777	IF
mouse IgG (HRP)	Santa Cruz Biotechnology (sc-2005)	1:2500 (0.16 µg/ml)	AB_631736	IB
rabbit IgG (Alexa Fluor 488)	Thermo Fisher Scientific (A-21206)	1:400 (5 µg/ml)	AB_2535792	IF
rabbit IgG (Alexa Fluor 647)	Thermo Fisher Scientific (A-31573)	1:400 (5 µg/ml)	AB_2536183	IF
rabbit IgG (Cy3)	Jackson ImmunoResearch Labs (711-165-152)	1:400 (3.75 µg/ml)	AB_2307443	IF
rabbit IgG (HRP)	Santa Cruz Biotechnology (sc2301)	1:5000 (0.08 µg/ml)	AB_650500	IB
rat IgG (Alexa Fluor 488)	Thermo Fisher Scientific (A-21208)	1:400 (5 µg/ml)	AB_2535794	IF
rat IgG (Alexa Fluor 555)	Abcam (ab150154)	1:400 (5 µg/ml)	AB_2813834	IF
rat IgG (Alexa Fluor 647)	Abcam (ab150155)	1:400 (5 µg/ml)	AB_2813835	IF
rat IgG (Cy3)	Jackson ImmunoResearch Labs (712-165-150)	1:400 (3.75 µg/ml)	AB_2340666	IF
rat IgG (StarORANGE)	Abberior (STORANGE-1007-500UG)	1:500	AB_2833017	IF, STED

Supplementary Table 5. Results of statistical analyses.

Figure	Sample size (n)	Statistical test	Values
1c	Controls: 23 patients<br>COVID-19: 17 patients	Two-tailed Mann-Whitney U test	$p = 0.015$
1d	6 patients/group	Two-tailed Mann-Whitney U test	$P = 0.015$
1f	4 hamsters/group	One-way ANOVA followed by Tukey's post-hoc tests (*p*-values adjusted for multiple comparisons)	F (3,12) = 3.6, $p = 0.048$<br><br>Control vs. Day 4: $p = 0.042$<br>Control vs. Day 7: $p = 0.728$<br>Control vs. Day 24: $p = 0.933$<br>Day 4 vs. Day 7: $p = 0.231$<br>Day 4 vs. Day 25: $p = 0.113$<br>Day 7 vs. Day 25: $p = 0.968$
1g	Control: 5 mice<br>Day 2: 3 mice<br>Day 7: 3 mice	One-way ANOVA followed by Tukey's post-hoc tests (*p*-values adjusted for multiple comparisons)	F (2,8) = 14.3, $p = 0.002$<br><br>Control vs. Day 2: $p = 0.617$<br>Control vs. Day 7: $p = 0.005$<br>Day 2 vs. Day 7: $p = 0.003$
4a	3 wells/group	Two-way ANOVA followed by Tukey's post-hoc tests (*p*-values adjusted for multiple comparisons)	M^{pro}: F (1,8) = 301.9, $p < 0.0001$;<br>treatment: F (1,8) = 1215, $p < 0.0001$;<br>interaction: F (1,8) = 449.9, $p < 0.0001$;<br><br>PBS-Control vs. PBS-M^{pro}: $p = 0.1$;<br>PBS-Control vs. IL-1β-Control: $p < 0.0001$;<br>PBS-M^{pro} vs. IL-1β -M^{pro}: $p < 0.0001$;<br>IL-1β -Control vs. IL-1β -M^{pro}: $p < 0.0001$
4b	6 wells/group	Scheirer-Ray-Hare test followed by targeted Mann-Whitney U tests, Bonferroni-Holm corrected	M^{pro}: $\chi 2$ (1) = 3.9, $p = 0.049$;<br>treatment: $\chi 2$ (1) = 4.3, $p = 0.037$;<br>interaction: $\chi 2$ (1) = 2.6, $p = 0.106$;<br><br>IL-1β -Control vs. IL-1β -M^{pro}: $p = 0.026$;<br>PBS-Control vs. IL-1β-Control: $p = 0.004$
4c	6 wells/group except IL-1β -M^{pro}:5 wells	Two-way ANOVA followed by Tukey's post-hoc tests (*p*-values adjusted for multiple comparisons)	M^{pro}: F (1,19) = 13.8, p = 0.002;<br>treatment: F (1,19) = 10.9, p = 0.004;<br>interaction: F (1,19) = 13.8, p = 0.002<br><br>PBS-Control vs. PBS-M^{pro}: $p > 0.9999$;<br>PBS-Control vs. IL-1β-Control: $p = 0.0004$<br>PBS-M^{pro} vs. IL-1β -M^{pro}: $p = 0.992$;<br>IL-1β -Control vs. IL-1β -M^{pro}: $p = 0.0003$
4e	12 wells/group	Scheirer-Ray-Hare test followed by targeted Mann-Whitney U tests, Bonferroni-Holm corrected	M^{pro}: $\chi 2$(1) = 11.3, $p < 0.001$;<br>treatment: χ2(1) = 0.4, p = 0.528;<br>interaction: χ2(1) = 0.4, p = 0.528;<br><br>TNF -Control vs. TNF -M^{pro}: $p = 0.057$;<br>PBS-Control vs. PBS- M^{pro}: $p = 0.057$
4g GFP	6 wells/group	One-way ANOVA followed by Tukey's post-hoc tests (*p*-values adjusted for multiple comparisons)	F (2,15) = 25.2, $p < 0.0001$;<br><br>Control vs. M^{pro}: $p < 0.0001$<br>Control vs. C145A-M^{pro}: $p = 0.162$;<br>M^{pro} vs. C145A-M^{pro}: $p = 0.0005$
4g HA	6 wells/group	Kruskal-Wallis test followed by targeted Dunn's post-tests (*p*-values adjusted for multiple comparisons)	$\chi 2$(2) = 15.8, $p < 0.0001$;<br><br>M^{pro} vs. C145A-M^{pro}: $p = 0.047$;
4h	6 wells/group	Scheirer-Ray-Hare test followed by targeted Mann-Whitney U tests, Bonferroni-Holm corrected	M^{pro}: $\chi 2$(2) = 0.03, $p = 0.996$;<br>treatment: $\chi 2$(1) = 26.3, $p < 0.001$;<br>interaction: $\chi 2$(2) = 3.5, $p = 0.171$;<br><br>IL-1β -Control vs. IL-1β -M^{pro}: $p = 0.0044$;<br>IL-1β -M^{pro} vs. IL-1β - C145A-M^{pro}: $p = 0.0044$
4k	Control: 9 mice<br>M^{pro}: 10 mice<br>C145A-M^{pro}: 10 mice	Welch's ANOVA followed by Tamhane T2 post-hoc tests (*p*-values adjusted for multiple comparisons)	W (2,11.9) = 25,2, p < 0.0001;<br><br>Control vs. M^{pro}: $p = 0.008$;<br>Control vs. C145A-M^{pro}: $p = 0.025$;<br>M^{pro} vs. C145A-M^{pro}: $p = 0.0003$
4l	Control: 9 mice<br>M^{pro}: 10 mice<br>C145A-M^{pro}: 10 mice	One-way ANOVA followed by Tukey's post-hoc tests (*p*-values adjusted for multiple comparisons)	F (2,26) = 4.7, $p = 0.019$;<br><br>Control vs. M^{pro}: $p = 0.014$;<br>Control vs. C145A-M^{pro}: $p = 0.356$;<br>M^{pro} vs. C145A-M^{pro}: $p = 0.229$

5e	5 mice/group	One-tailed Mann-Whitney U test	p = 0.004
5f	3 mice/group	Repeated measures ANOVA followed by Sidak's post-hoc tests (p-values adjusted for multiple comparisons)	Genotype: F (1,4) = 171.6, p = 0.0002;<br>branch order: F (1.9,7.8) = 103.9, p < 0.0001;<br>interaction: F (4,16) = 63.83, p < 0.0001;<br><br>*Nemo*^{fl} vs. *Nemo*^{beKO}:<br>arteriol: p = 0.936;<br>1st capillary: p > 0.9999;<br>2nd capillary: p = 0.177;<br>3rd capillary: p = 0.994;<br>> 3rd capillary/venous: p = 0.006
5g	3 mice/group	Descriptives only	
5h	*Nemo*^{fl}: 10 mice<br>*Nemo*^{beKO}: 7 mice	Two-tailed Mann-Whitney U test	p = 0.002
5i	*Nemo*^{fl}: 6 mice<br>*Nemo*^{beKO}: 8 mice	Two-tailed unpaired t test	T (12) = 2.7, p = 0.019
5j left	*Nemo*^{fl}: 5 mice<br>*Nemo*^{beKO}: 4 mice	Two-tailed unpaired t test	T (7) = 4.1, p = 0.004
5j right	*Nemo*^{fl}: 4 mice<br>*Nemo*^{beKO}: 3 mice	Two-tailed unpaired t test (Welch corrected)	T (2.4) = 4.1, p = 0.039
5k	*Nemo*^{fl}: 5 mice<br>*Nemo*^{beKO}: 4 mice	Two-tailed unpaired t test (Welch corrected)	T (3.0) = 11.1, p = 0.002
6a left	*Nemo*^{fl}: 17 mice<br>*Nemo*^{beKO}: 12 mice<br>*Ripk3*$^{-/-}$: 9 mice<br>*Nemo*^{beKO}*Ripk3*$^{-/-}$: 8 mice	Kruskal Wallis test followed by Dunn's post-hoc tests (p-values adjusted for multiple comparisons)	χ^2(3) = 27.6, p < 0.0001;<br><br>*Nemo*^{fl} vs. *Ripk3*$^{-/-}$: p > 0.9999;<br>*Nemo*^{fl} vs. *Nemo*^{beKO}: p < 0.0001;<br>*Nemo*^{fl} vs. *Nemo*^{beKO}*Ripk3*$^{-/-}$: p > 0.9999;<br>*Ripk3*$^{-/-}$ *vs. Nemo*^{beKO}: p = 0.0004;<br>*Ripk3*$^{-/-}$ *vs. Nemo*^{beKO}*Ripk3*$^{-/-}$: p > 0.9999;<br>*Nemo*^{beKO} vs. *Nemo*^{beKO}*Ripk3*$^{-/-}$: p = 0.02
6a middle	*Nemo*^{fl}: 17 mice<br>*Nemo*^{beKO}: 12 mice<br>*Ripk3*$^{-/-}$: 9 mice<br>*Nemo*^{beKO}*Ripk3*$^{-/-}$: 8 mice	One-way ANOVA followed by Tukey's post-hoc tests (p-values adjusted for multiple comparisons)	F (3,42) = 10.4, p < 0.0001;<br><br>*Nemo*^{fl} vs. *Ripk3*$^{-/-}$: p = 0.984;<br>*Nemo*^{fl} vs. *Nemo*^{beKO}: p < 0.0001;<br>*Nemo*^{fl} vs. *Nemo*^{beKO}*Ripk3*$^{-/-}$: p = 0.646;<br>*Ripk3*$^{-/-}$ *vs. Nemo*^{beKO}: p = 0.0008;<br>*Ripk3*$^{-/-}$ *vs. Nemo*^{beKO}*Ripk3*$^{-/-}$: p = 0.883;<br>*Nemo*^{beKO} vs. *Nemo*^{beKO}*Ripk3*$^{-/-}$: p = 0.012
6a right	*Nemo*^{fl}: 7 mice<br>*Nemo*^{beKO}: 5 mice<br>*Ripk3*$^{-/-}$: 6 mice<br>*Nemo*^{beKO}*Ripk3*$^{-/-}$: 6 mice	Kruskal Wallis test followed by Dunn's post-hoc tests (p-values adjusted for multiple comparisons)	χ^2(3) = 12.8, p = 0.005;<br><br>*Nemo*^{fl} vs. *Ripk3*$^{-/-}$: p > 0.9999;<br>*Nemo*^{fl} vs. *Nemo*^{beKO}: p = 0.008;<br>*Nemo*^{fl} vs. *Nemo*^{beKO}*Ripk3*$^{-/-}$: p > 0.9999;<br>*Ripk3*$^{-/-}$ *vs. Nemo*^{beKO}: p = 0.011;<br>*Ripk3*$^{-/-}$ *vs. Nemo*^{beKO}*Ripk3*$^{-/-}$: p > 0.9999;<br>*Nemo*^{beKO} vs. *Nemo*^{beKO}*Ripk3*$^{-/-}$: p = 0.130
6b	*Nemo*^{fl}: 17 mice<br>*Nemo*^{beKO}: 13 mice<br>*Ripk3*$^{-/-}$: 10 mice<br>*Nemo*^{beKO}*Ripk3*$^{-/-}$: 8 mice	Log Rank Mantel-Cox test (*Nemo*^{fl} vs. *Nemo*^{beKO})	p = 0.015
6c left	*Nemo*^{fl}: 17 mice<br>*Nemo*^{beKO}: 11 mice<br>*Ripk3*$^{-/-}$: 10 mice<br>*Nemo*^{beKO}*Ripk3*$^{-/-}$: 8 mice	Kruskal Wallis test followed by Dunn's post-hoc tests (p-values adjusted for multiple comparisons)	χ^2(3) = 21.4, p < 0.0001<br><br>*Nemo*^{fl} vs. *Ripk3*$^{-/-}$: p > 0.9999;<br>*Nemo*^{fl} vs. *Nemo*^{beKO}: p < 0.0001;<br>*Nemo*^{fl} vs. *Nemo*^{beKO}*Ripk3*$^{-/-}$: p > 0.9999;<br>*Ripk3*$^{-/-}$ *vs. Nemo*^{beKO}: p = 0.013;<br>*Ripk3*$^{-/-}$ *vs. Nemo*^{beKO}*Ripk3*$^{-/-}$: p > 0.9999;<br>*Nemo*^{beKO} vs. *Nemo*^{beKO}*Ripk3*$^{-/-}$: p = 0.003
6c middle	8 mice/group	Kruskal Wallis test followed by Dunn's post-hoc tests (p-values adjusted for multiple comparisons)	χ^2(3) = 25.0, p < 0.0001;<br><br>*Nemo*^{fl} vs. *Ripk3*$^{-/-}$: p > 0.9999;<br>*Nemo*^{fl} vs. *Nemo*^{beKO}: p = 0.001;<br>*Nemo*^{fl} vs. *Nemo*^{beKO}*Ripk3*$^{-/-}$: p = 0.123;<br>*Ripk3*$^{-/-}$ *vs. Nemo*^{beKO}: p < 0.0001;<br>*Ripk3*$^{-/-}$ *vs. Nemo*^{beKO}*Ripk3*$^{-/-}$: p = 0.019;<br>*Nemo*^{beKO} vs. *Nemo*^{beKO}*Ripk3*$^{-/-}$: p = 0.901
6c right	8 mice/group	Kruskal Wallis test followed by Dunn's post-hoc tests (p-values adjusted for multiple comparisons)	χ^2(3) = 14.6, p = 0.002;<br><br>*Nemo*^{fl} vs. *Ripk3*$^{-/-}$: p > 0.9999;<br>*Nemo*^{fl} vs. *Nemo*^{beKO}: p = 0.001;<br>*Nemo*^{fl} vs. *Nemo*^{beKO}*Ripk3*$^{-/-}$: p = 0.241;

			$Ripk3^{-/-}$ vs. $Nemo^{beKO}$: p = 0.058;<br>$Ripk3^{-/-}$ vs. $Nemo^{beKO}Ripk3^{-/-}$: p > 0.9999;<br>$Nemo^{beKO}$ vs. $Nemo^{beKO}Ripk3^{-/-}$: p = 0.624
6d upper	$Nemo^{fl}$: 7 mice<br>$Nemo^{beKO}$: 5 mice<br>$Ripk3^{-/-}$: 6 mice<br>$Nemo^{beKO}Ripk3^{-/-}$: 6 mice	One-way ANOVA with Tukey's post-hoc tests (p-values adjusted for multiple comparisons)	F (3,20) = 6.5, p = 0.003;<br><br>$Nemo^{fl}$ vs. $Ripk3^{-/-}$: p = 0.842;<br>$Nemo^{fl}$ vs. $Nemo^{beKO}$: p = 0.017;<br>$Nemo^{fl}$ vs. $Nemo^{beKO}Ripk3^{-/-}$: p = 0.966;<br>$Ripk3^{-/-}$ vs. $Nemo^{beKO}$: p = 0.039;<br>$Ripk3^{-/-}$ vs. $Nemo^{beKO}Ripk3^{-/-}$: p = 0.985;<br>$Nemo^{beKO}$ vs. $Nemo^{beKO}Ripk3^{-/-}$: p = 0.008
6d lower	$Nemo^{fl}$: 7 mice<br>$Nemo^{beKO}$: 5 mice<br>$Ripk3^{-/-}$: 6 mice<br>$Nemo^{beKO}Ripk3^{-/-}$: 6 mice	Kruskal Wallis test followed by Dunn's post-hoc tests (p-values adjusted for multiple comparisons)	χ^2(3) = 12.9, p = 0.005;<br><br>$Nemo^{fl}$ vs. $Ripk3^{-/-}$: p > 0.9999;<br>$Nemo^{fl}$ vs. $Nemo^{beKO}$: p = 0.003;<br>$Nemo^{fl}$ vs. $Nemo^{beKO}Ripk3^{-/-}$: p > 0.9999;<br>$Ripk3^{-/-}$ vs. $Nemo^{beKO}$: p = 0.043;<br>$Ripk3^{-/-}$ vs. $Nemo^{beKO}Ripk3^{-/-}$: p > 0.9999;<br>$Nemo^{beKO}$ vs. $Nemo^{beKO}Ripk3^{-/-}$: p = 0.144
6e	3 mice/group	One-way ANOVA followed by Tukey's post-hoc tests (p-values adjusted for multiple comparisons)	F (3,8) = 72.7, p < 0.001;<br><br>$Nemo^{fl}$ vs. $Ripk3^{-/-}$: p = 0.284;<br>$Nemo^{fl}$ vs. $Nemo^{beKO}$: p < 0.0001;<br>$Nemo^{fl}$ vs. $Nemo^{beKO}Ripk3^{-/-}$: p = 0.429;<br>$Ripk3^{-/-}$ vs. $Nemo^{beKO}$: p < 0.0001;<br>$Ripk3^{-/-}$ vs. $Nemo^{beKO}Ripk3^{-/-}$: p = 0.985;<br>$Nemo^{beKO}$ vs. $Nemo^{beKO}Ripk3^{-/-}$: p < 0.0001
6f left	$Nemo^{fl}$: 6 mice<br>$Nemo^{beKO}$: 8 mice<br>$Ripk3^{-/-}$: 4 mice<br>$Nemo^{beKO}Ripk3^{-/-}$: 3 mice	One-way ANOVA followed by Tukey's post-hoc tests (p-values adjusted for multiple comparisons)	F (3,17) = 16.7, p < 0.0001;<br><br>$Nemo^{fl}$ vs. $Ripk3^{-/-}$: p = 0.995;<br>$Nemo^{fl}$ vs. $Nemo^{beKO}$: p < 0.0001;<br>$Nemo^{fl}$ vs. $Nemo^{beKO}Ripk3^{-/-}$: p = 0.021;<br>$Ripk3^{-/-}$ vs. $Nemo^{beKO}$: p = 0.0004;<br>$Ripk3^{-/-}$ vs. $Nemo^{beKO}Ripk3^{-/-}$: p = 0.050;<br>$Nemo^{beKO}$ vs. $Nemo^{beKO}Ripk3^{-/-}$: p = 0.440
6f right	$Nemo^{fl}$: 6 mice<br>$Nemo^{beKO}$: 8 mice<br>$Ripk3^{-/-}$: 4 mice<br>$Nemo^{beKO}Ripk3^{-/-}$: 3 mice	One-way ANOVA followed by Tukey's post-hoc tests (p-values adjusted for multiple comparisons)	F (3,17) = 37.4, p < 0.0001;<br><br>$Nemo^{fl}$ vs. $Ripk3^{-/-}$: p = 0.490;<br>$Nemo^{fl}$ vs. $Nemo^{beKO}$: p < 0.0001;<br>$Nemo^{fl}$ vs. $Nemo^{beKO}Ripk3^{-/-}$: p = 0.0009<br>$Ripk3^{-/-}$ vs. $Nemo^{beKO}$: p < 0.0001;<br>$Ripk3^{-/-}$ vs. $Nemo^{beKO}Ripk3^{-/-}$: p = 0.0001;<br>$Nemo^{beKO}$ vs. $Nemo^{beKO}Ripk3^{-/-}$: p = 0.437
7b	5 mice/group	Two-way ANOVA followed by Tukey's post-hoc tests (p-values adjusted for multiple comparisons)	Genotype: F (1,16) = 9,7, p = 0.007;<br>M^{pro}: F (1,16) = 15.9, p = 0.0011;<br>interaction: F (1,16) = 0.1, p = 0.719;<br><br>$Ripk3^{+/+}$-Control vs. $Ripk3^{+/+}$- M^{pro}: p = 0.033<br>$Ripk3^{+/+}$-Control vs. $Ripk3^{-/-}$-Control: p = 0.251<br>$Ripk3^{+/+}$-Control vs. $Ripk3^{-/-}$- M^{pro}: p = 0.926<br>$Ripk3^{+/+}$- M^{pro} vs. $Ripk3^{-/-}$- Control: p = 0.0007<br>$Ripk3^{+/+}$- M^{pro} vs. $Ripk3^{-/-}$- M^{pro}: p = 0.105<br>$Ripk3^{-/-}$- Control vs. $Ripk3^{-/-}$- M^{pro}: p = 0.089
7d	Control-Vehicle: 5 mice<br>M^{pro}-Vehicle: 6 mice<br>Control-RIPKi: 6 mice<br>M^{pro}- RIPKi: 6 mice	Two-way ANOVA followed by Tukey's post-hoc tests (p-values adjusted for multiple comparisons)	treatment: F (1,19) = 9.4, p = 0.006;<br>M^{pro}: F (1,19) = 0.44, p = 0.517;<br>interaction: F (1,19) = 2.5, p = 0.131;<br><br>Control-vehicle vs. M^{pro}-vehicle: p = 0.431;<br>Control-vehicle vs. control- RIPKi: p = 0.739;<br>Control-vehicle vs. M^{pro}-RIPKi: p = 0.372;<br>M^{pro}-vehicle vs. control- RIPKi: p = 0.063;<br>M^{pro}-vehicle vs. M^{pro} - RIPKi: p = 0.016;<br>Control- RIPKi vs. M^{pro}-RIPKi: p = 0.908;
Ext. 2b	Controls: 23 patients<br>COVID-19: 17 patients	Scheirer-Ray-Hare test	patient group: χ^2(1) = 5.32, p = 0.021;<br>sex: χ^2(1) = 0.17, p = 0.680;<br>interaction: $\chi 2$(1) = 0.23, p = 0.632;
Ext. 2c	Controls: 23 patients<br>COVID-19: 17 patients	Descriptives only	

Ext. 2d	Controls: 23 patients<br>COVID-19: 17 patients	Scheirer-Ray-Hare test	patient group: $\chi^2(1) = 5.99$, $p = 0.014$;<br>cerebral comorbidities: $\chi^2(1) = 0.01$, $p = 0.920$;<br>interaction: $\chi 2(1) = 0.77$, $p = 0.380$;
Ext. 2e	Controls: 23 patients<br>COVID-19: 17 patients	Two-tailed Mann-Whitney U test	$p = 0.404$
Ext. 2f	Controls: 23 patients<br>COVID-19: 17 patients	Two-tailed Mann-Whitney U test	$p = 0.252$
Ext.2g	Controls: 23 patients<br>COVID-19: 17 patients	Two-tailed Mann-Whitney U test	$p = 0.0009$
Ext. 2h	10 patients/group	Descriptives only	-
Ext. 2i	No ICU: 6 patients<br>ICU: 16 patients	Descriptives only	-
Ext. 8b left	3 mice/group	Repeated measures ANOVA followed by Sidak's post-hoc tests (*p*-values adjusted for multiple comparisons)	genotype: F (1,4) = 77.2, $p = 0.0009$;<br>branch order: F (1.8,7.2) = 95.0, $p < 0.0001$;<br>interaction: F (4,16) = 62.32, $p < 0.0001$;<br><br>$Nemo^{fl}$ vs. $Nemo^{beKO}$:<br>arteriol: $p > 0.9999$;<br>1st capillary: $p = 0.699$;<br>2nd capillary: $p = 0.864$;<br>3rd capillary: $p = 0.621$;<br>> 3rd capillary/venous: $p < 0.0001$
Ext. 8b right	3 mice/group	Repeated measures ANOVA followed by Sidak's post-hoc tests (*p*-values adjusted for multiple comparisons)	genotype: F (1,4) = 67.2, $p = 0.001$;<br>branch order: F (1.6,6.2) = 153.2, $p < 0.0001$;<br>interaction: F (4,16) = 45.23, $p < 0.0001$;<br><br>$Nemo^{fl}$ vs. $Nemo^{beKO}$:<br>arteriol: $p = 0.999$;<br>1st capillary: $p = 0.116$;<br>2nd capillary: $p = 0.687$;<br>3rd capillary: $p = 0.205$;<br>> 3rd capillary/venous: $p < 0.0001$
Ext. 9b left	$Nemo^{fl}$: 17 mice<br>$Nemo^{beKO}$: 12 mice<br>$Ripk3^{-/-}$: 9 mice<br>$Nemo^{beKO}Ripk3^{-/-}$: 8 mice<br>$Fadd^{beKO}Ripk3^{-/-}$: 6 mice<br>$Nemo^{beKO}Fadd^{beKO}Ripk3^{-/-}$: 8 mice<br>$Fadd^{beKO}$: 8 mice<br>$Nemo^{beKO}Fadd^{beKO}$: 9 mice	Descriptives only	-
Ext. 9b right	$Nemo^{fl}$: 17 mice<br>$Nemo^{beKO}$: 12 mice<br>$Ripk3^{-/-}$: 9 mice<br>$Nemo^{beKO}Ripk3^{-/-}$: 8 mice<br>$Fadd^{beKO}Ripk3^{-/-}$: 6 mice<br>$Nemo^{beKO}Fadd^{beKO}Ripk3^{-/-}$: 8 mice<br>$Fadd^{beKO}$: 8 mice<br>$Nemo^{beKO}Fadd^{beKO}$: 9 mice	Descriptives only	-
Ext. 9d left	$Nemo^{fl}$: 8 mice<br>$Nemo^{beKO}$: 8 mice<br>$Ripk3^{-/-}$: 8 mice<br>$Nemo^{beKO}Ripk3^{-/-}$: 8 mice<br>$Fadd^{beKO}Ripk3^{-/-}$: 5 mice<br>$Nemo^{beKO}Fadd^{beKO}Ripk3^{-/-}$: 8 mice<br>$Fadd^{beKO}$: 8 mice<br>$Nemo^{beKO}Fadd^{beKO}$: 8 mice	Descriptives only	-
Ext. 9d middle	$Nemo^{fl}$: 8 mice<br>$Nemo^{beKO}$: 8 mice<br>$Ripk3^{-/-}$: 8 mice<br>$Nemo^{beKO}Ripk3^{-/-}$: 8 mice<br>$Fadd^{beKO}Ripk3^{-/-}$: 5 mice	Descriptives only	-

	$Nemo^{beKO}Fadd^{beKO}Ripk3^{-/-}$: 8 mice<br>$Fadd^{beKO}$: 8 mice<br>$Nemo^{beKO}Fadd^{beKO}$: 8 mice		
Ext. 9d right	$Nemo^{fl}$: 17 mice<br>$Nemo^{beKO}$: 11 mice<br>$Ripk3^{-/-}$: 10 mice<br>$Nemo^{beKO}Ripk3^{-/-}$: 8 mice<br>$Fadd^{beKO}Ripk3^{-/-}$: 6 mice<br>$Nemo^{beKO}Fadd^{beKO}Ripk3^{-/-}$: 8 mice<br>$Fadd^{beKO}$: 8 mice<br>$Nemo^{beKO}Fadd^{beKO}$: 9 mice	Descriptives only	-
Ext. 9e	$Nemo^{fl}$: 17 mice<br>$Nemo^{beKO}$: 13 mice<br>$Ripk3^{-/-}$: 10 mice<br>$Nemo^{beKO}Ripk3^{-/-}$: 8 mice<br>$Fadd^{beKO}Ripk3^{-/-}$: 6 mice<br>$Nemo^{beKO}Fadd^{beKO}Ripk3^{-/-}$: 9 mice<br>$Fadd^{beKO}$: 8 mice<br>$Nemo^{beKO}Fadd^{beKO}$: 9 mice	Descriptives only	-
Ext. 9f	$Nemo^{fl}$: 17 mice<br>$Nemo^{beKO}$: 9 mice<br>$Ripk3^{-/-}$: 10 mice<br>$Nemo^{beKO}Ripk3^{-/-}$: 8 mice<br>$Fadd^{beKO}Ripk3^{-/-}$: 6 mice<br>$Nemo^{beKO}Fadd^{beKO}Ripk3^{-/-}$: 8 mice<br>$Fadd^{beKO}$: 7 mice<br>$Nemo^{beKO}Fadd^{beKO}$: 9 mice	Descriptives only	-
Ext. 10a	Ctrl + Vehicle: 5 mice<br>M^{pro} + Vehicle: 6 mice<br>Ctrl + RIPKi: 6 mice<br>M^{pro} + RIPKi: 6 mice	Repeated measures two-way ANOVA	Group: F (3,19) = 2.1, p = 0.130;<br>Day: F (5.3,100.5) = 18.5, p < 0.0001;<br>interaction: F (39,247) = 4.9, p < 0.0001
Ext. 10b	Ctrl + Vehicle: 5 mice<br>M^{pro} + Vehicle: 6 mice<br>Ctrl + RIPKi: 6 mice<br>M^{pro} + RIPKi: 6 mice	Two-way ANOVA followed by Tukey's post-hoc tests (p-values adjusted for multiple comparisons)	M^{pro}: F (1,19) = 4.2, p = 0.054;<br>treatment: F (1,19) = 21.0, p = 0.0002;<br>interaction: F (1,19) = 3.0, p = 0.101;<br><br>Ctrl + Vehicle vs. M^{pro} + Vehicle: p = 0.075;<br>Ctrl + Vehicle vs. Ctrl + RIPKi: p = 0.232;<br>M^{pro} + Vehicle vs. M^{pro} + RIPKi: p = 0.001;<br>Ctrl + RIPKi vs. M^{pro} + RIPKi: p = 0.995
Sup. 1a	4 hamsters/group	Kruskal-Wallis test	$\chi^2(3)$ = 3.5, p = 0.349
Sup. 1b	Control: 5 mice<br>Day 2: 3 mice<br>Day 7: 3 mice	Kruskal-Wallis test	$\chi^2(2)$ = 2.1, p = 0.393
Sup. 4b	Control: 9 mice<br>M^{pro}: 9 mice<br>C145A-M^{pro}: 10 mice	One-way ANOVA	F (2,25) = 1.7, p = 0.21
Sup. 4c	M^{pro}: 6 mice<br>C145A-M^{pro}: 10 mice	Two-tailed unpaired t test	T (14) = 2.6, p = 0.023
Sup. 5c	$Nemo^{fl}$: 9 mice<br>$Nemo^{beKO}$: 10 mice	Two-tailed Mann-Whitney U test	p < 0.0001
Sup. 6b left (Microglia number)	$Nemo^{fl}$ + Control: 5 mice<br>$Nemo^{beKO}$ + Control: 4 mice<br>$Nemo^{fl}$ + PLX: 5 mice<br>$Nemo^{beKO}$ + PLX: 6 mice	Two-way ANOVA followed by Tukey's post-hoc tests (p-values adjusted for multiple comparisons)	genotype: F (1,16) = 18.9, p = 0.0005;<br>diet: F (1,16) = 764.7, p < 0.0001;<br>interaction: F (1,16) = 5.5, p = 0.033;<br><br>$Nemo^{fl}$ + Control vs. $Nemo^{beKO}$ + Control: p = .002;<br>$Nemo^{fl}$ + Control vs. $Nemo^{fl}$ + PLX: p <.0001;<br>$Nemo^{fl}$ + Control vs. $Nemo^{beKO}$ + PLX: p < .0001;

			Nemo^{beKO} + Control vs. *Nemo*^{fl} + PLX: p <.0001;<br>*Nemo*^{beKO} + Control vs. *Nemo*^{beKO} + PLX: p < .0001;<br>*Nemo*^{fl} + PLX vs. *Nemo*^{beKO} + PLX: p = .460
Sup. 6b middle left (IgG extravasation)	*Nemo*^{fl} + Control: 5 mice<br>*Nemo*^{beKO} + Control: 4 mice<br>*Nemo*^{fl} + PLX: 5 mice<br>*Nemo*^{beKO} + PLX: 6 mice	Scheirer-Ray-Hare test	genotype: $\chi^2(1) = 14.1$, $p < 0.001$;<br>diet: $\chi^2(1) = 0.002$, $p = 0.967$;<br>interaction: $\chi^2(1) = 0.01$, $p = 0.920$
Sup. 6b middle right (String vessel length)	*Nemo*^{fl} + Control: 3 mice<br>*Nemo*^{beKO} + Control: 3 mice<br>*Nemo*^{fl} + PLX: 5 mice<br>*Nemo*^{beKO} + PLX: 6 mice	Scheirer-Ray-Hare test	genotype: $\chi^2(1) = 9.5$, $p = 0.002$;<br>diet: $\chi^2(1) = 0.2$, $p = 0.671$;<br>interaction: $\chi^2(1) = 0.6$, $p = 0.458$
Sup. 6b right (Astrocyte number)	*Nemo*^{fl} + Control: 5 mice<br>*Nemo*^{beKO} + Control: 4 mice<br>*Nemo*^{fl} + PLX: 5 mice<br>*Nemo*^{beKO} + PLX: 6 mice	Scheirer-Ray-Hare test	genotype: $\chi^2(1) = 13.9$, $p < 0.001$;<br>treatment: $\chi^2(1) = 1.0$, $p = 0.310$;<br>interaction: $\chi^2(1) = 0.7$, $p = 0.392$
Sup. 7b	4 mice/group	Two-tailed Mann-Whitney U test	$p = 0.029$
Sup. 7c	*Nemo*^{fl}: 4 mice<br>*Nemo*^{beKO}: 5 mice	Two-tailed unpaired t test	$T(7) = 1.9$, $p = 0.103$

Supplementary Figure 1

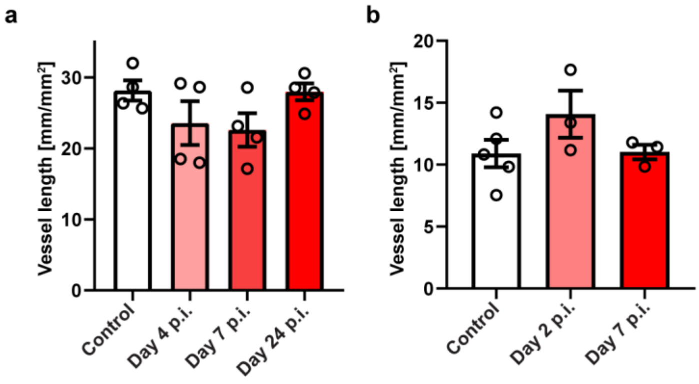

Supplementary Fig. 1: The density of caveolin-1-positive vessels was not changed over time in SARS-CoV-2-infected hamsters or K18-hACE2 mice.

a, Quantification of caveolin-1-positive vessel length per mm^2 in hamsters 4, 7 and 24 days after SARS-CoV-2 infection and uninfected control hamsters (N = 4). **b**, Quantification of caveolin-1-positive vessel lengths per mm^2 in K18-hACE2 mice 2 and 7 days after SARS-CoV-2 infection and uninfected control K18-hACE2 mice (N = 5 control mice, 3 mice 2 p. i., 3 mice 7 p.i.). Means ± SEM are shown.

Supplementary Figure 2

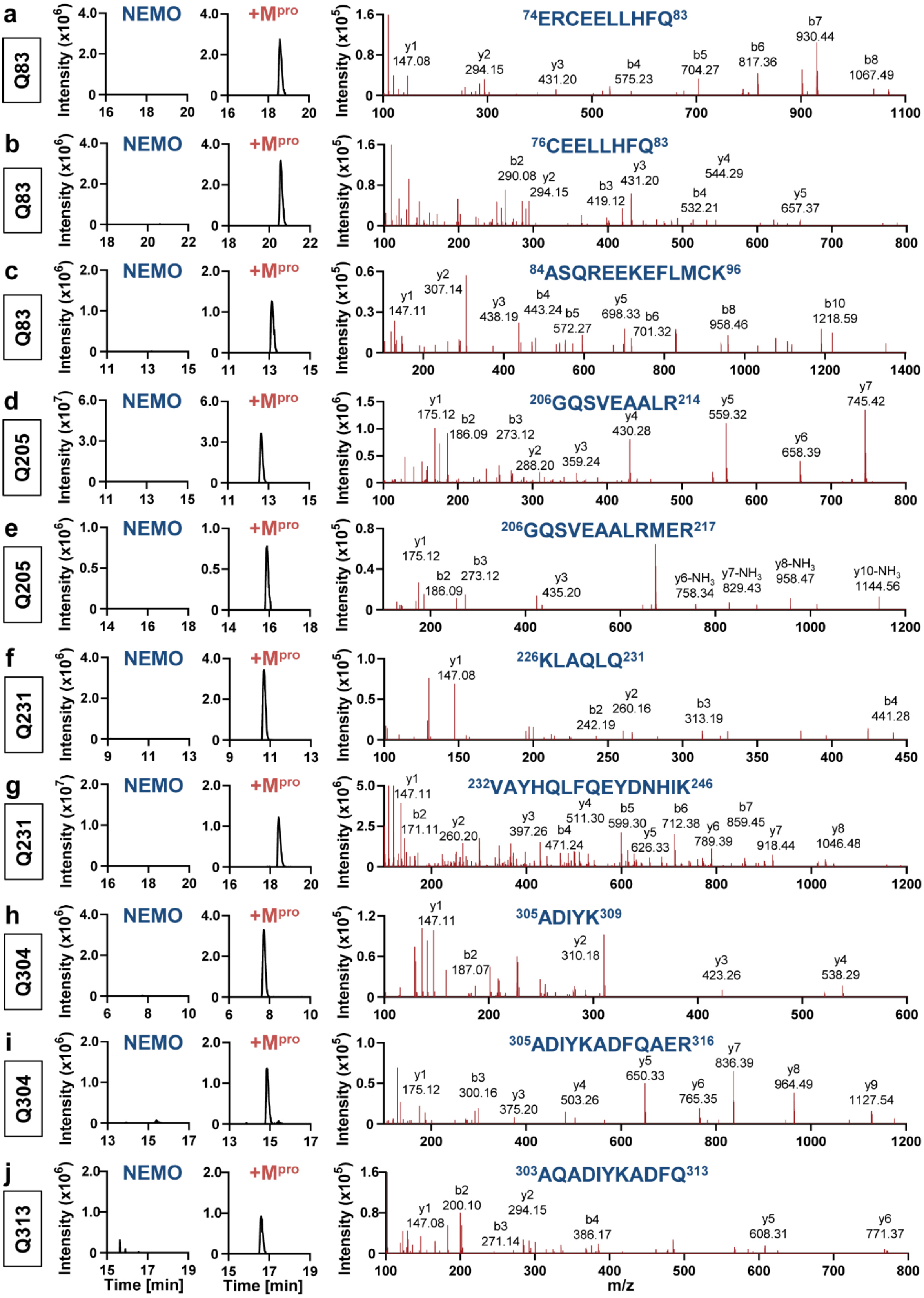

k

Human NEMO sequence with M^{pro} cleavage sites

1 MNRHLWKSQLCEMVQPSGGPAADQDVLGEESPLGKPAMLHLPSEQGAPETLQRCLEENQE 60

61 LRDAIRQSNQILRERCEELLHF**Q**ASQREEKEFLMCKFQEARKLVERLGLEKLDLKRQKEQ 120

121 ALREVEHLKRCQQQMAEDKASVKAQVTSLLGELQESQSRLEAATKECQALEGRARAASEQ 180

181 ARQLESEREALQQQHSVQVDQLRM**Q**GQSVEAALRMERQAASEEKRKLAQL**Q**VAYHQLFQE 240

241 YDNHIKSSVVGSERKRGMQLEDLKQQLQQAEEALVAKQEVIDKLKEEAEQHKIVMETVPV 300

301 LKA**Q**ADIYKADF**Q**AERQAREKLAEKKELLQEQLEQLQREYSKLKASCQESARIEDMRKRH 360

361 VEVSQAPLPPAPAYLSSPLALPSQRRSPPEEPPDFCCPKCQYQAPDMDTLQIHVMECIE 419

Supplementary Fig. 2: Tryptic digestion and MS analysis showing that human NEMO is cleaved by M^{pro} at Q83, Q205, Q231, Q304, and Q313.

a, Extracted ion chromatogram (EIC) of the tryptic peptide ^{74}ERCEELLHFQ^{83} (m/z, 680.8210^{2+}; retention time [RT], 18.6 min) derived from NEMO that was treated with M^{pro} (5 µM) and its MS/MS spectrum. **b,** EIC of the tryptic peptide ^{76}CEELLHFQ^{83} (m/z, 538.2484^{2+}; RT, 20.6 min) derived from NEMO that was treated with M^{pro} (5 µM) and its MS/MS spectrum. **c,** EIC of the tryptic peptide ^{84}ASQREEKEFLMCK^{96} (m/z, 828.3980^{2+}; RT, 13.1 min) derived from NEMO that was treated with M^{pro} (5 µM) and its MS/MS spectrum. **d,** EIC of the tryptic peptide ^{206}GQSVEAALR^{214} (m/z, 465.7550^{2+}; RT, 12.7 min) derived from NEMO that was treated with M^{pro} (5 µM) and its MS/MS spectrum. **e,** EIC of the tryptic peptide ^{206}GQSVEAALRMER^{217} (m/z, 673.8467^{2+}; RT, 15.9 min) derived from NEMO that was treated with M^{pro} (5 µM) and its MS/MS spectrum. **f,** EIC of the tryptic peptide ^{226}KLAQLQ^{231} (m/z, 350.7215^{2+}; RT, 10.7 min) derived from NEMO that was treated with M^{pro} (5 µM) and its MS/MS spectrum. **g,** EIC of the tryptic peptide ^{232}VAYHQLFQEYDNHIK^{246} (m/z, 635.6492^{3+}; RT, 18.4 min) derived from NEMO that was treated with M^{pro} (5 µM) and its MS/MS spectrum. **h,** EIC of the tryptic peptide ^{305}ADIYK^{309} (m/z, 305.1663^{2+}; RT, 7.7 min) derived from NEMO that was treated with M^{pro} (5 µM) and its MS/MS spectrum. **i,** EIC of the tryptic peptide ^{305}ADIYKADFQAER^{316} (m/z, 713.8524^{2+}; RT, 14.9 min) derived from NEMO that was treated with M^{pro} (5 µM) and its MS/MS spectrum. **j,** EIC of the tryptic peptide ^{303}AQADIYKADFQ^{313} (m/z, 635.3097^{2+}; RT, 16.6 min) derived from NEMO that was treated with M^{pro} (5 µM) and its MS/MS spectrum. **k,** Protein sequence of human NEMO (Genebank ID: AAC36330.1), in which the five cleavage sites are labeled.

Supplementary Figure 3

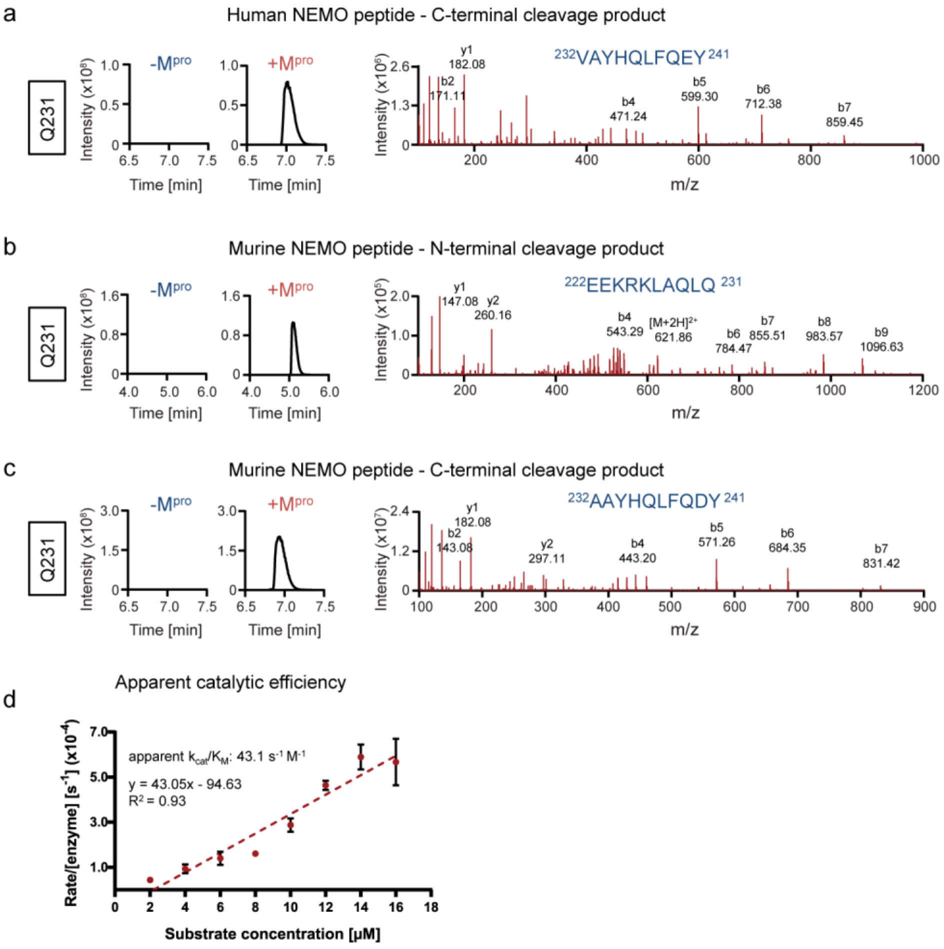

Supplementary Fig. 3: Sequence specificity and catalytic efficiency of NEMO cleavage by M^{pro}.

a, Extracted ion chromatogram (EIC) of the peptide ^{232}VAYHQLFQEY^{241} (m/z, 649.3149^{2+}; retention time [RT], 7.0 min) after incubation of the synthetic peptide h-NEMO_222-241 (EEKRKLAQLQVAYHQLFQEY) without and with M^{pro} (2.5 µM) and its MS/MS spectrum. **b,** EIC of the peptide ^{222}EEKRKLAQLQ^{231} (m/z, 414.9104^{3+}; RT, 5.1 min) after incubation of the synthetic peptide m-NEMO_222-241 (EEKRKLAQLQAAYHQLFQDY) without and with M^{pro} (2.5 µM) and its MS/MS spectrum. **c,** EIC of the peptide ^{232}AAYHQLFQDY^{241} (m/z, 628.2915^{2+}; RT, 6.9 min) after incubation of the synthetic peptide m-NEMO_222-241 (EEKRKLAQLQAAYHQLFQDY) without and with M^{pro} (2.5 µM) and its MS/MS spectrum. **d,** Determination of the apparent catalytic efficiency k_{cat}/K_M by incubating the synthetic peptide h-NEMO_222-241 (EEKRKLAQLQVAYHQLFQEY) with M^{pro} (2.5 µM). The concentration of proteolysis product h-NEMO_222-231 was quantified by LC-MS/MS, using synthetic peptide h-NEMO_222-231 for calibration, and was converted to initial reaction rate. Initial reaction rates were normalized by the concentration of M^{pro} and plotted against substrate concentrations. The apparent k_{cat}/K_M was obtained from the slope of the curve. Experiments were performed as biological triplicates. Data are shown as means ± SEM.

Supplementary Figure 4

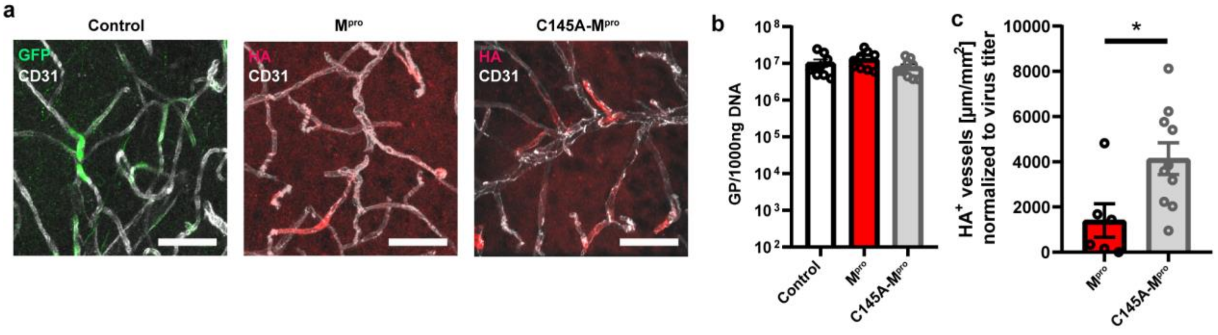

Supplementary Fig. 4: Transduction of brain endothelial cells in mice with AAV-BR1 vectors.

a, Representative staining of expressed proteins in cortical vessels of mice, two weeks after AAV-BR1-GFP (control, $3.3x10^{11}$ gp, 100 µl, i.v.), AAV-BR1-M^{pro} (M^{pro}, $3.3x10^{11}$ gp, 100 µl, i.v.), or AAV-BR1-C145A-M^{pro} (C145A-M^{pro}, $3.3x10^{11}$ gp, 100 µl, i.v.) were injected. Sections were stained for GFP (control) or the HA-tagged M^{pro} and C145A-M^{pro} in combination with the endothelial marker CD31. Scale bar, 100 µm (representative for the dataset that was used for quantification in (c)). **b**, Accumulation of genomic particles (gp) in PFA-fixed brains of mice, two weeks after AAV-BR1-M^{pro} ($3.3x10^{11}$ gp, 100 µl, i.v., N = 9 mice), AAV-BR1-GFP (control, $3.3x10^{11}$ gp, 100 µl, i.v., N = 9 mice) or AAV-BR1-C145A-M^{pro} ($3.3x10^{11}$ gp, 100 µl, i.v., N = 10 mice) injection. Genomic particles were quantified by q-PCR. **c**, Quantification of HA-positive vessel length normalized to virus titer that was measured in the brains of mice that received AAV-BR1-M^{pro} (N = 6 mice) or AAV-BR1-C145A-M^{pro} (N = 10 animals). Data are shown as means ± SEM. Detailed information about the exact test statistics, sidedness and values are provided in Supplementary Table 5.

Supplementary Figure 5

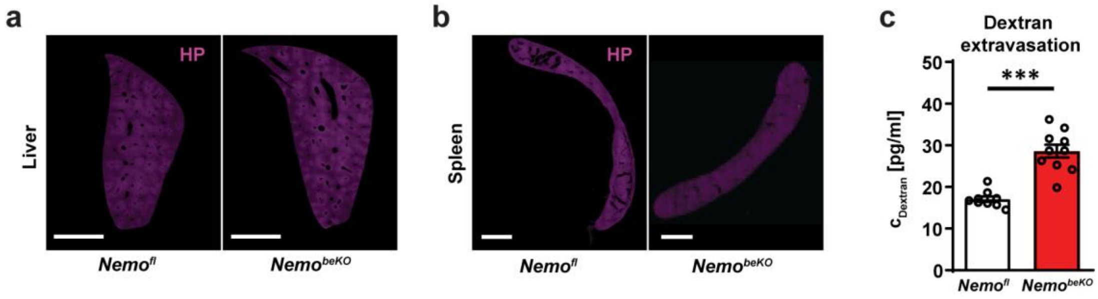

Supplementary Fig. 5: No evidence for hypoxia in liver and spleen and a tracer study confirming the blood-brain barrier disruption in *Nemo*^{beKO} mice.

a, b, Staining of the hypoxia probe (HP) provided no evidence for hypoxic areas in liver and spleen of *Nemo*^{beKO} mice, indicating that hypoxia is limited to the central nervous system (representative for 3 animals per genotype). Mice were treated with pimonidazole 1 hour before perfusion. Scale bar, 2 mm. **c,** Increased concentrations of 4-kDa-FITC-dextran in brain lysates of *Nemo*^{beKO} mice (N = 10 mice) indicated a disrupted BBB in comparison to *Nemo*^{fl} controls (N = 9 animals). Mice were treated with 4-kDa-FITC-dextran 30 min before perfusion. Data are shown as means ± SEM, ***$p < 0.001$. Detailed information about the exact test statistics, sidedness and values are provided in Supplementary Table 5.

Supplementary Figure 6

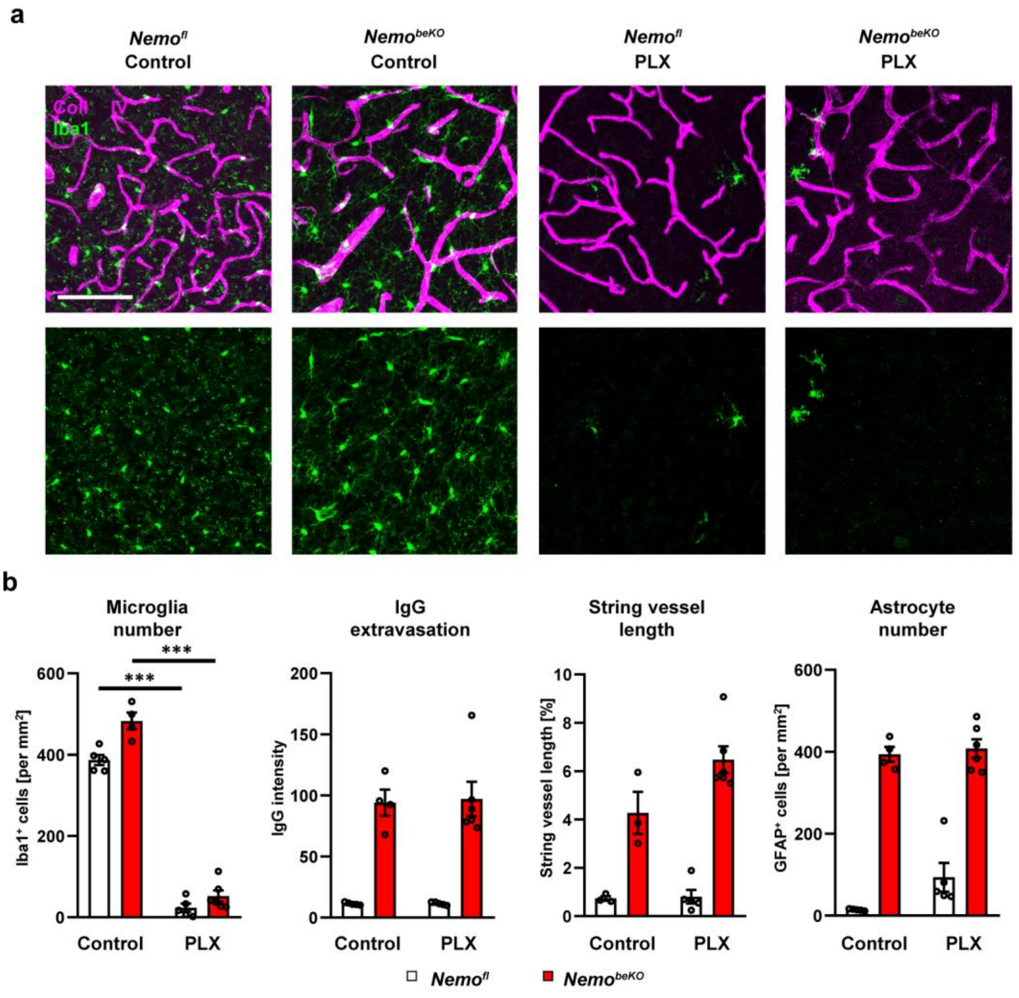

Supplementary Fig. 6: Ablation of microglia does not improve the microvascular pathology in mice with endothelial NEMO deficiency.

a, Administering the CSF-1R antagonist PLX5622 (PLX) reduced microglia in *Nemo*^{fl} and *Nemo*^{beKO} animals. Representative images from (b) are shown. Scale bar, 100 µm. **b,** Left, compared to the control diet, the PLX diet strongly reduced the density of microglia in all genotypes: *Nemo*^{fl}+Control, N = 5 mice; *Nemo*^{beKO}+Control, N = 4 mice; *Nemo*^{fl}+PLX, N = 5 mice; *Nemo*^{beKO}+PLX, N = 6 mice. Middle left, ablating microglia by PLX diet did not prevent the disruption of the BBB as shown by IgG extravasation: *Nemo*^{fl}+Control, N = 5 mice; *Nemo*^{beKO}+Control, N = 4 mice; *Nemo*^{fl}+PLX, N = 5 mice; *Nemo*^{beKO}+PLX, N = 6 mice. Middle right, ablating microglia by PLX diet did not reduce the formation of string vessels: *Nemo*^{fl}+Control, N = 3 mice; *Nemo*^{beKO}+Control, N = 3 mice; *Nemo*^{fl}+PLX, N = 5 mice; *Nemo*^{beKO}+PLX, N = 6 mice. Right, ablating microglia did not affect the activation of astrocytes: *Nemo*^{fl}+Control, N = 5 mice; *Nemo*^{beKO}+Control, N = 4 mice; *Nemo*^{fl}+PLX, N = 5 mice; *Nemo*^{beKO}+PLX, N = 6 mice). Means ± SEM are shown, ***$p < 0.001$. Detailed information about the exact test statistics, sidedness and values are provided in Supplementary Table 5.

Supplementary Figure 7

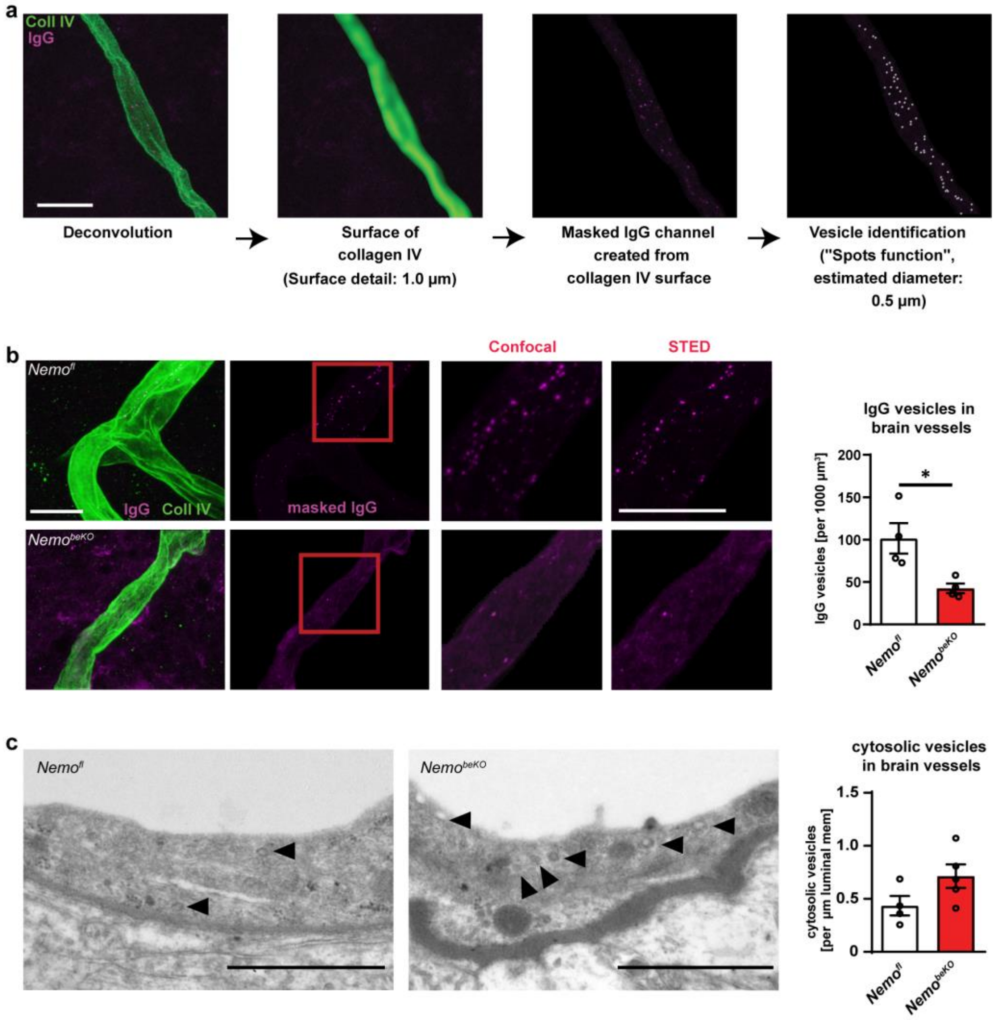

Supplementary Fig. 7: Super-resolution imaging techniques allow detailed analysis of brain endothelial transcytosis in *Nemo*^{beKO} mice

a, Scheme showing the procedure of IgG vesicle analysis in vessels. Deconvolution of confocal images was performed by Huygens software. The following steps were done using Imaris 9.3.0. First, a surface of collagen IV was created with a surface detail of 1.0 µm (0.4 µm for STED images). Then, the collagen IV surface was used to mask the IgG channel. Finally, IgG-filled vesicles were identified and counted by the "Spots function" of Imaris using an estimated diameter of 0.5 µm. Scale bar, 10 µm. **b,** STED microscopy captures more IgG-filled vesicles than conventional confocal microscopy. STED images of *Nemo*^{fl} and *Nemo*^{beKO} showed an increased number of IgG-filled vesicles with apparent smaller diameter compared to the corresponding confocal images (left). Quantification of STED images demonstrated a similar decrease of IgG-filled vesicles in *Nemo*^{beKO} mice as confocal images (Fig. 6f, right panel). Scale bar, 10 µm. N = 4 animals/genotype. **c,** Analysis of electron microscopy images showed no differences in the number of vesicles in brain capillaries of *Nemo*^{beKO} (N = 5 mice) and *Nemo*^{fl} controls (N = 4 mice). We analyzed 3 vessels/animal. Scale bar, 1 µm. Means ± SEM are shown, * $p < 0.05$. Detailed information about the exact test statistics, sidedness and values are provided in Supplementary Table 5.
