# Supplementary material for: The SARS-CoV-2 main protease Mpro causes microvascular brain pathology by cleaving NEMO in brain endothelial cells
Source: Nat Neurosci. 2021 Oct 21;24(11):1522–33. doi: 10.1038/s41593-021-00926-1 (PMC8553622; doi:10.1038/s41593-021-00926-1)
Supplement: Supplementary file 2 — Reporting Summary [file 41593_2021_926_MOESM2_ESM.pdf]

nature research

Corresponding author(s): Markus Schwaninger

Last updated by author(s): July 29, 2021

Reporting Summary

Nature Research wishes to improve the reproducibility of the work that we publish. This form provides structure for consistency and transparency in reporting. For further information on Nature Research policies, see our Editorial Policies and the Editorial Policy Checklist.

Statistics

For all statistical analyses, confirm that the following items are present in the figure legend, table legend, main text, or Methods section.

n/a	Confirmed	
☐	☒	The exact sample size (n) for each experimental group/condition, given as a discrete number and unit of measurement
☐	☒	A statement on whether measurements were taken from distinct samples or whether the same sample was measured repeatedly
☐	☒	The statistical test(s) used AND whether they are one- or two-sided<br>*Only common tests should be described solely by name; describe more complex techniques in the Methods section.*
☒	☐	A description of all covariates tested
☐	☒	A description of any assumptions or corrections, such as tests of normality and adjustment for multiple comparisons
☐	☒	A full description of the statistical parameters including central tendency (e.g. means) or other basic estimates (e.g. regression coefficient) AND variation (e.g. standard deviation) or associated estimates of uncertainty (e.g. confidence intervals)
☐	☒	For null hypothesis testing, the test statistic (e.g. F, t, r) with confidence intervals, effect sizes, degrees of freedom and P value noted<br>*Give P values as exact values whenever suitable.*
☒	☐	For Bayesian analysis, information on the choice of priors and Markov chain Monte Carlo settings
☒	☐	For hierarchical and complex designs, identification of the appropriate level for tests and full reporting of outcomes
☒	☐	Estimates of effect sizes (e.g. Cohen's d, Pearson's r), indicating how they were calculated

Our web collection on statistics for biologists contains articles on many of the points above.

Software and code

Policy information about availability of computer code

Data collection	None
Data analysis	ImageJ (v 1.52p), Imaris (v.9.3.0), Seurat package (v.3.2.0), kallisto (v.0.46.1), bustools (v.0.46.1), R software (v.4.2.0), DropletUtils (v.1.8.0), Xcalibur software (v.3.0.63), MaxQuant software (v.1.6.1.0), GraphPad Prism 8 (v.8), SPSS 25 (v.25), MotionTracking (MPI-CBG, v8)

For manuscripts utilizing custom algorithms or software that are central to the research but not yet described in published literature, software must be made available to editors and reviewers. We strongly encourage code deposition in a community repository (e.g. GitHub). See the Nature Research guidelines for submitting code & software for further information.

Data

Policy information about availability of data

All manuscripts must include a data availability statement. This statement should provide the following information, where applicable:

- Accession codes, unique identifiers, or web links for publicly available datasets
- A list of figures that have associated raw data
- A description of any restrictions on data availability

RNAseq data is available at GEO: GSE180984 after July 31st 2021. Other data are available upon request.

Field-specific reporting

Please select the one below that is the best fit for your research. If you are not sure, read the appropriate sections before making your selection.

☒ Life sciences ☐ Behavioural & social sciences ☐ Ecological, evolutionary & environmental sciences

For a reference copy of the document with all sections, see nature.com/documents/nr-reporting-summary-flat.pdf

Life sciences study design

All studies must disclose on these points even when the disclosure is negative.

Sample size	No statistical methods were used to predetermine sample sizes, but our sample sizes are similar to those reported in previous publications. For other analyses, the availability of patient material determined the sample size.
Data exclusions	No data were excluded.
Replication	All experiments and analyses, apart from the analyses of patients, were replicated at least three times if not stated otherwise.
Randomization	For treatment allocation, mice were randomized.
Blinding	Whenever possible experimenters were blinded for genotype and treatment.

Reporting for specific materials, systems and methods

We require information from authors about some types of materials, experimental systems and methods used in many studies. Here, indicate whether each material, system or method listed is relevant to your study. If you are not sure if a list item applies to your research, read the appropriate section before selecting a response.

Materials & experimental systems

n/a	Involved in the study	
☐	☒	Antibodies
☐	☒	Eukaryotic cell lines
☒	☐	Palaeontology and archaeology
☐	☒	Animals and other organisms
☐	☒	Human research participants
☒	☐	Clinical data
☒	☐	Dual use research of concern

Methods

n/a	Involved in the study	
☒	☐	ChIP-seq
☒	☐	Flow cytometry
☒	☐	MRI-based neuroimaging

Antibodies

Antibodies used	Details are presented in the Supplementary Table 3 and 4.
Validation	Data on the validation of primary antibodies is presented in the Supplementary Table 2.

Eukaryotic cell lines

Policy information about cell lines

Cell line source(s)	bEnd.3 cells from ATCC (CRL-2299, RRID:CVCL_0170), hCMEC/D3 cells from Merck Millipore (SCC066, RRID: CVCL_U985).
Authentication	Morphology and gene expression
Mycoplasma contamination	Cells tested negative for mycoplasma contamination
Commonly misidentified lines (See ICLAC register)	None

Animals and other organisms

Policy information about studies involving animals; ARRIVE guidelines recommended for reporting animal research

Laboratory animals	Details are reported in the section "Animals" of the manuscript.

Wild animals	Not applicable
Field-collected samples	Not applicable
Ethics oversight	Details are reported in the section "Animals" of the manuscript.

Note that full information on the approval of the study protocol must also be provided in the manuscript.

Human research participants

Policy information about studies involving human research participants

Population characteristics	Details are reported in Supplementary Table 1 and 2.
Recruitment	Consecutive patients.
Ethics oversight	The study was approved by the local ethics committee of the Hamburg Chamber of Physicians and the University of Göttingen.

Note that full information on the approval of the study protocol must also be provided in the manuscript.
