# Supplementary figures and images for: The SARS-CoV-2 main protease Mpro causes microvascular brain pathology by cleaving NEMO in brain endothelial cells
Source: Nat Neurosci. 2021 Oct 21;24(11):1522–33. doi: 10.1038/s41593-021-00926-1 (PMC8553622; doi:10.1038/s41593-021-00926-1)

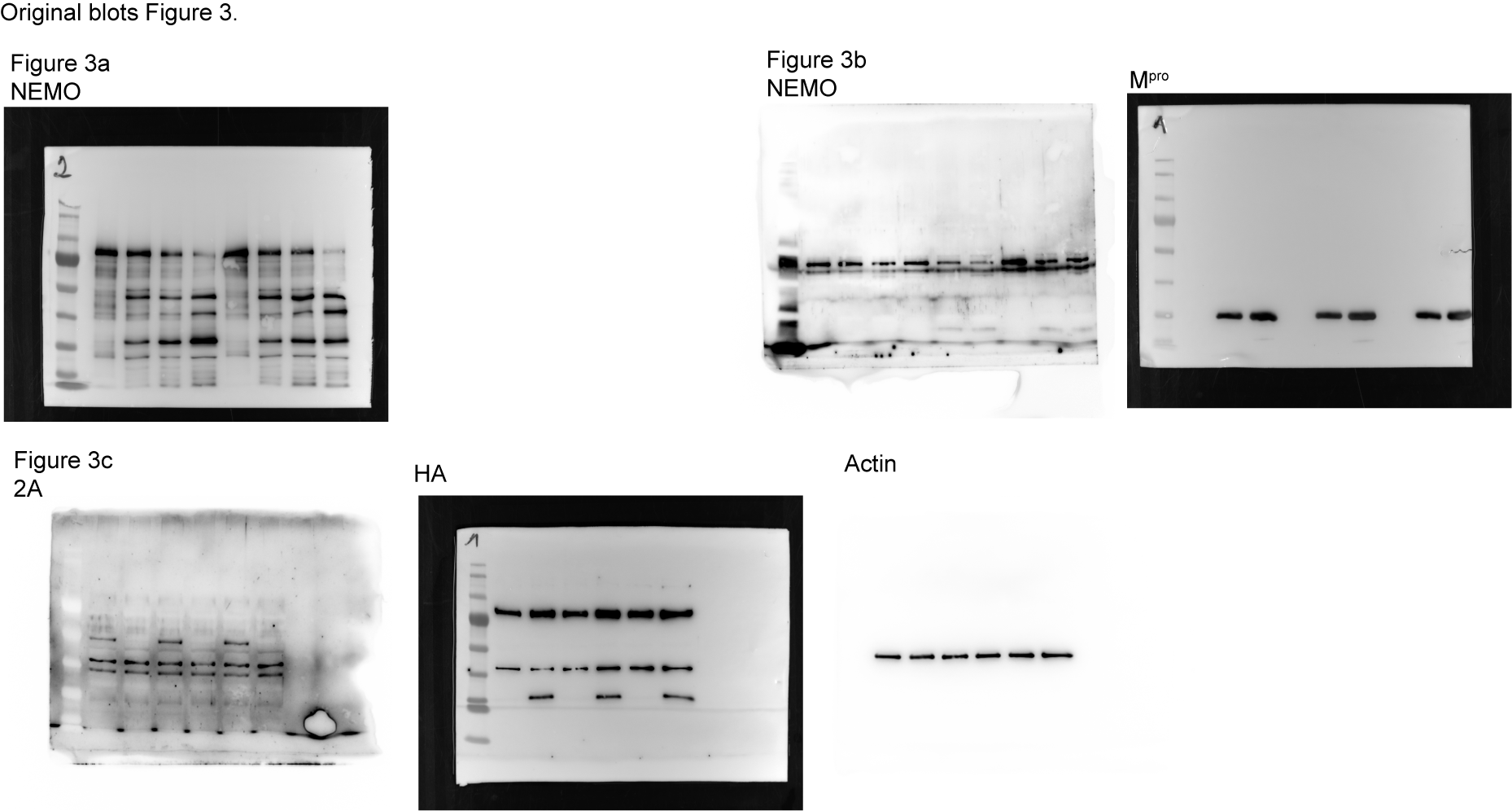

Supplement: Source Data Fig. 3 — Unprocessed western blots. [file 41593_2021_926_MOESM3_ESM.tif]

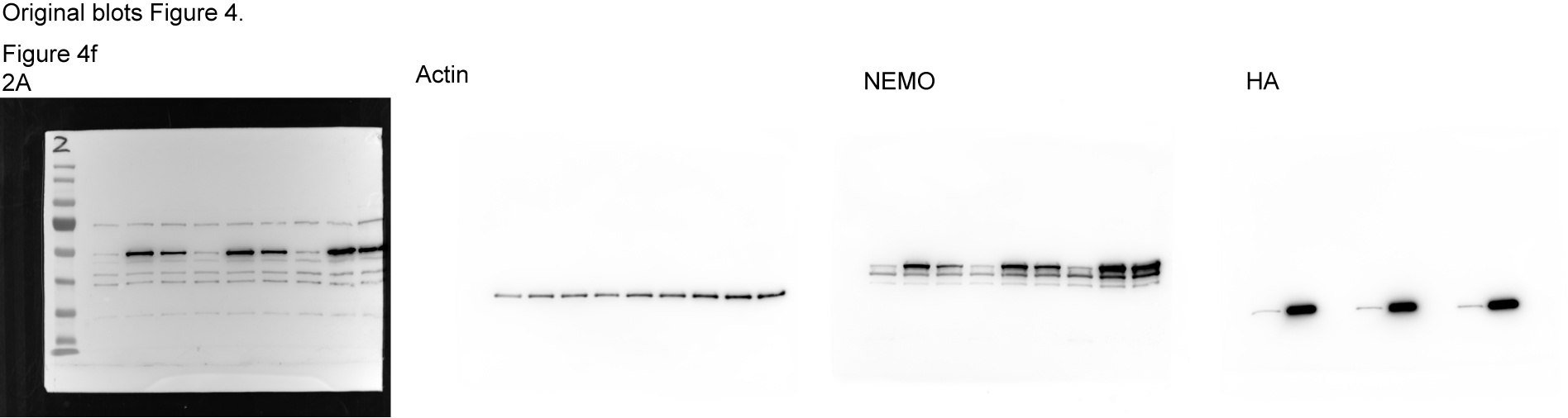

Supplement: Source Data Fig. 4 — Unprocessed western blots. [file 41593_2021_926_MOESM4_ESM.tif]

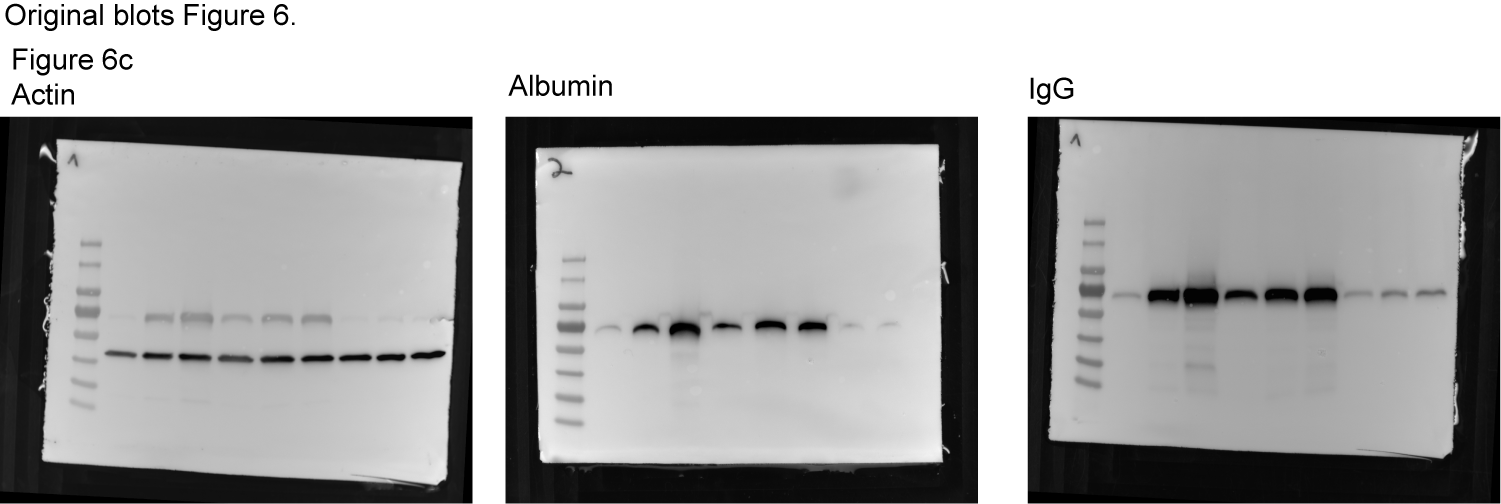

Supplement: Source Data Fig. 6 — Unprocessed western blots. [file 41593_2021_926_MOESM5_ESM.tif]

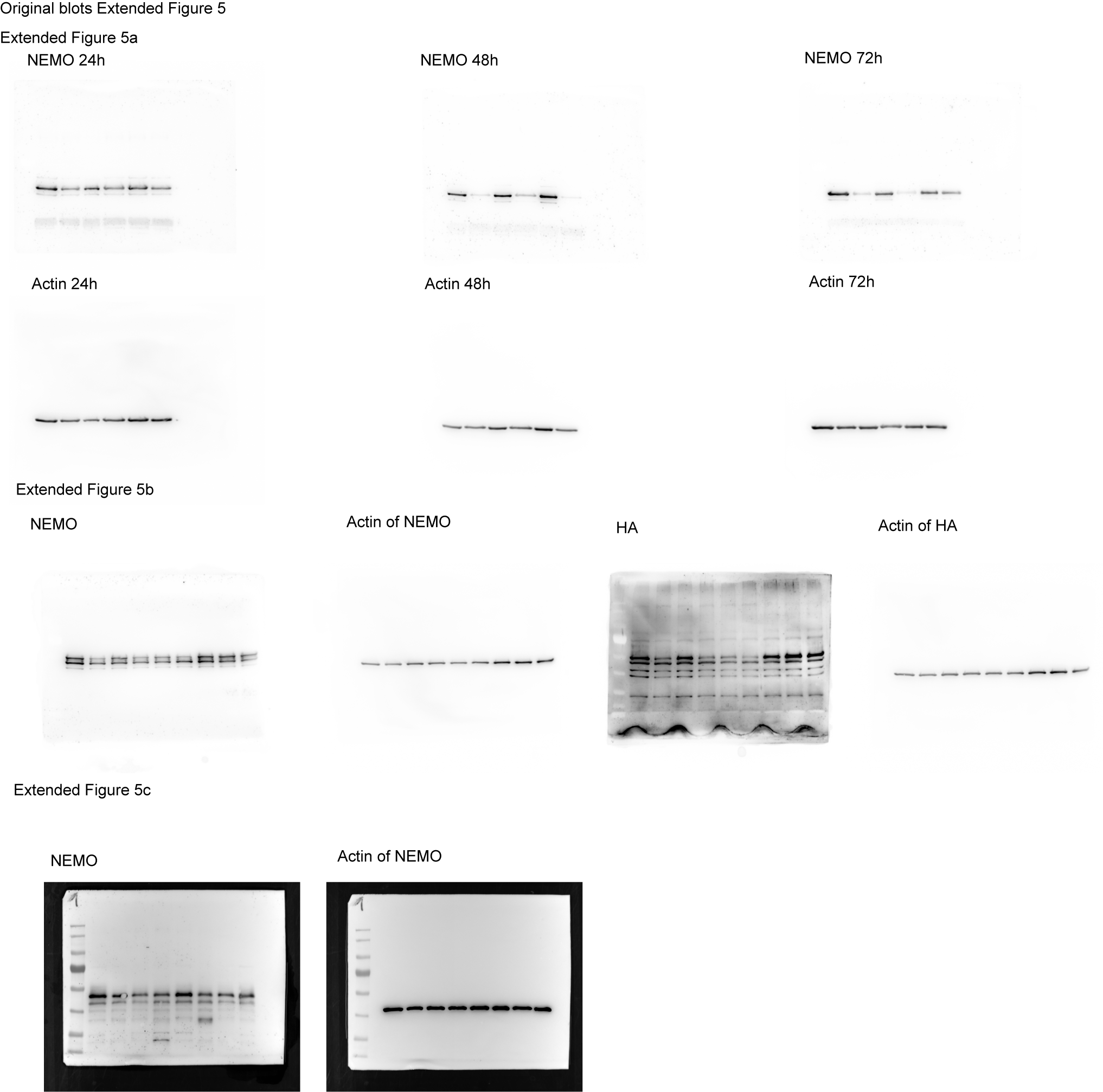

Supplement: Source Data Extended Data Fig. 5 — Unprocessed western blots. [file 41593_2021_926_MOESM6_ESM.tif]

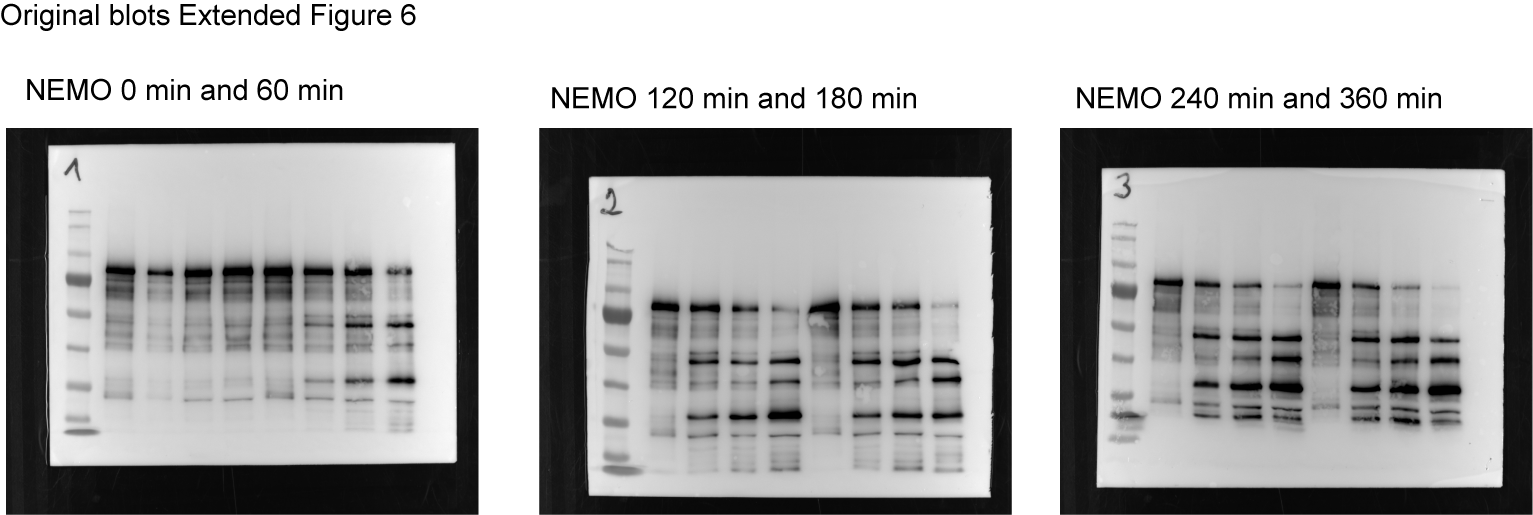

Supplement: Source Data Extended Data Fig. 6 — Unprocessed western blots. [file 41593_2021_926_MOESM7_ESM.tif]

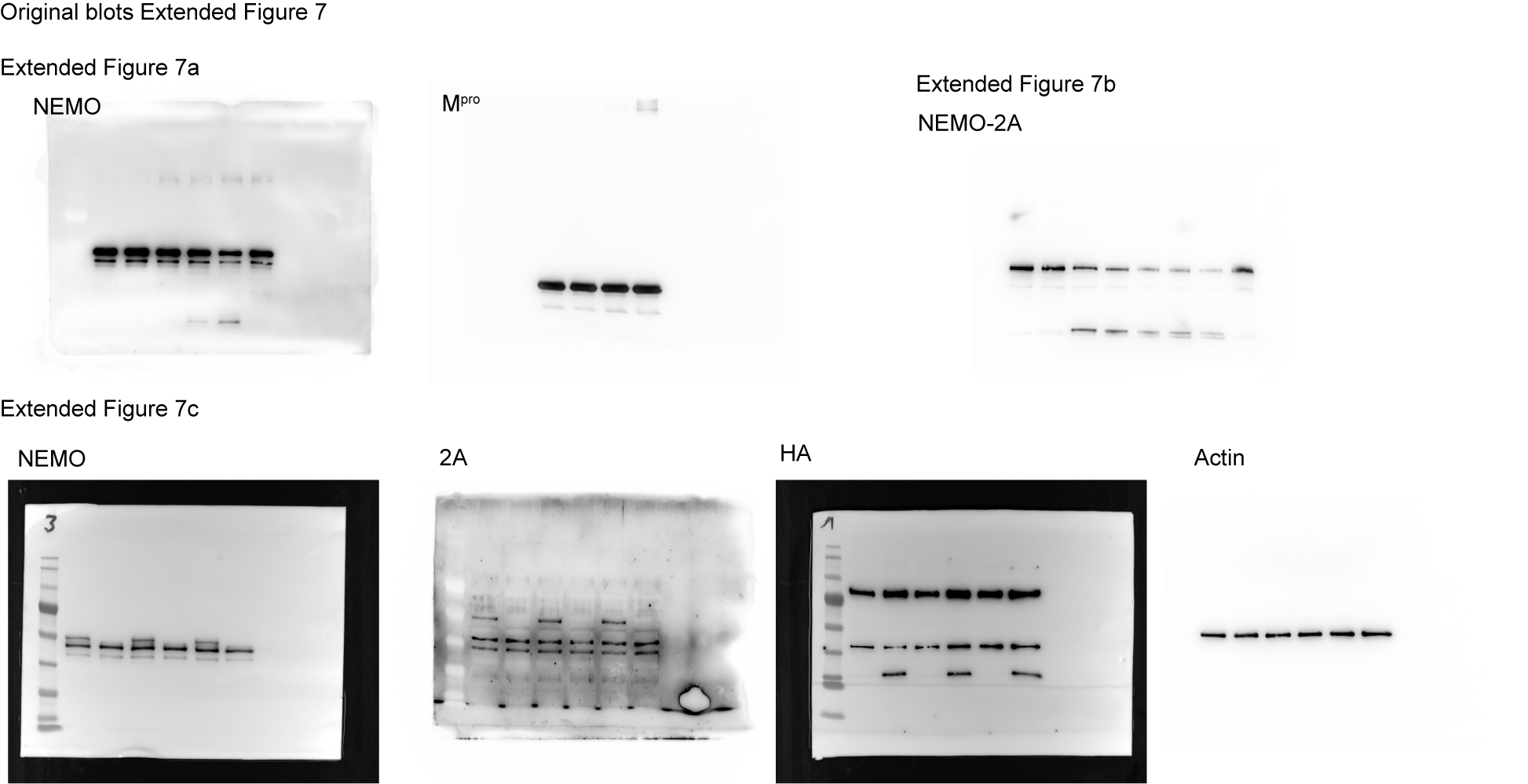

Supplement: Source Data Extended Data Fig. 7 — Unprocessed western blots. [file 41593_2021_926_MOESM8_ESM.tif]

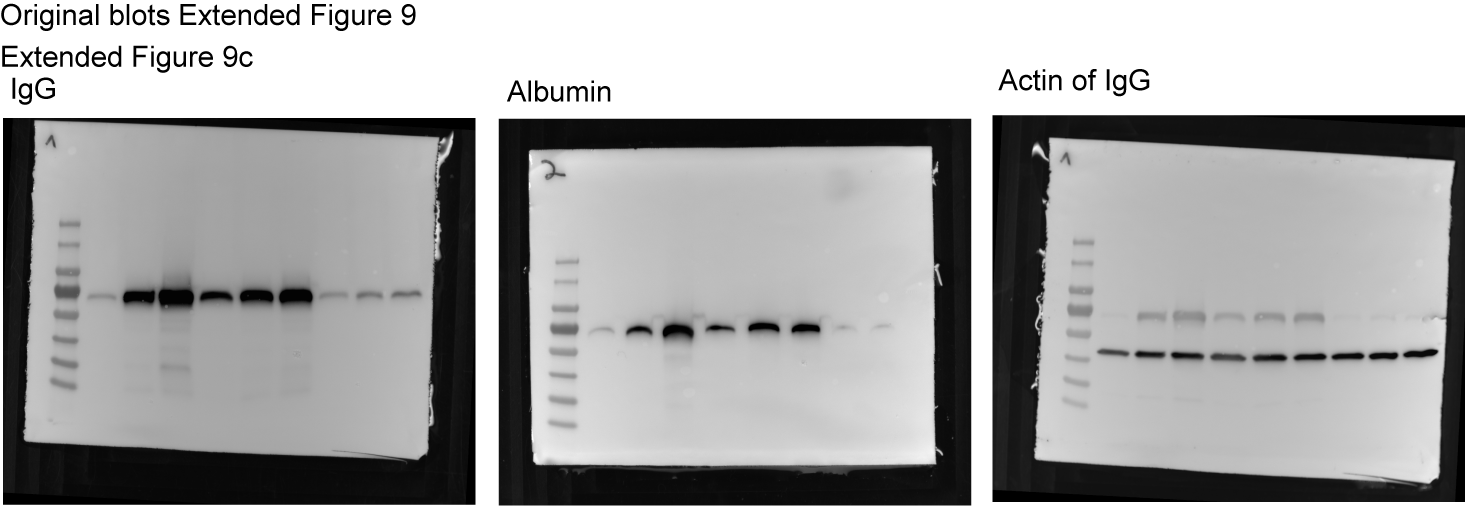

Supplement: Source Data Extended Data Fig. 9 — Unprocessed western blots. [file 41593_2021_926_MOESM9_ESM.tif]
